# Very early invasive strategy in higher risk non-ST-elevation acute coronary syndrome: the RAPID NSTEMI trial

**DOI:** 10.1136/heartjnl-2023-323513

**Published:** 2023-12-16

**Authors:** Thomas A Kite, Andrew Ladwiniec, John P Greenwood, Chris P Gale, Brijesh Anantharam, Ranjit More, Simon Lee Hetherington, Sohail Q Khan, Peter O'Kane, Roby Rakhit, Alexander Chase, Shaun Barber, Ghazala Waheed, Colin Berry, Marcus Flather, Gerry P McCann, Nick Curzen, Adrian P Banning, Anthony H Gershlick

**Affiliations:** 1 Department of Cardiovascular Sciences and the NIHR Leicester Biomedical Research Centre, Glenfield Hospital, University of Leicester and University Hospitals of Leicester NHS Trust, Leicester, UK; 2 Leeds Institute of Cardiovascular and Metabolic Medicine, University of Leeds and the Department of Cardiology Leeds Teaching Hospitals NHS Trust, Leeds, UK; 3 Portsmouth Hospitals University NHS Trust, Portsmouth, UK; 4 Blackpool Teaching Hospitals NHS Foundation Trust, Blackpool, UK; 5 Department of Cardiology, Kettering General Hospital NHS Foundation Trust, Kettering, UK; 6 Department of Cardiology, Queen Elizabeth Hospital, University Hospitals Birmingham NHS Foundation Trust, Birmingham, UK; 7 The Royal Bournemouth Hospital, University Hospitals Dorset NHS Foundation Trust, Bournemouth, UK; 8 Department of Cardiology, Royal Free Hospital and Institute of Cardiovascular Sciences, University College London, London, UK; 9 Department of Cardiology, Morriston Hospital, Swansea, UK; 10 Leicester Clinical Trials Unit, University of Leicester, Leicester, UK; 11 School of Cardiovascular and Metabolic Health, British Heart Foundation Glasgow Cardiovascular Research Centre, Golden Jubilee National Hospital and University of Glasgow, Glasgow, UK; 12 Norwich Medical School, University of East Anglia, Norwich, UK; 13 Faculty of Medicine, University of Southampton and University Hospital Southampton NHS Foundation Trust, Southampton, UK; 14 Department of Cardiology, John Radcliffe Hospital, Oxford University Hospitals NHS Trust, Oxford, UK

**Keywords:** acute coronary syndrome, percutaneous coronary intervention

## Abstract

**Objective:**

To investigate whether a very early invasive strategy (IS)±revascularisation improves clinical outcomes compared with standard care IS in higher risk patients with non-ST-elevation acute coronary syndrome (NSTE-ACS).

**Methods:**

Multicentre, randomised, controlled, pragmatic strategy trial of higher risk patients with NSTE-ACS, defined by Global Registry of Acute Coronary Events 2.0 score of ≥118, or ≥90 with at least one additional high-risk feature. Participants were randomly assigned to very early IS±revascularisation (<90 min from randomisation) or standard care IS±revascularisation (<72 hours). The primary outcome was a composite of all-cause mortality, new myocardial infarction or hospitalisation for heart failure at 12 months.

**Results:**

The trial was discontinued early by the funder due to slow recruitment during the COVID-19 pandemic. 425 patients were randomised, of whom 413 underwent an IS: 204 to very early IS (median time from randomisation: 1.5 hours (IQR: 0.9–2.0)) and 209 to standard care IS (median: 44.0 hours (IQR: 22.9–72.6)). At 12 months, there was no significant difference in the primary outcome between the early IS (5.9%) and standard IS (6.7%) groups (OR 0.93, 95% CI 0.42 to 2.09; p=0.86). The incidence of stroke and major bleeding was similar. The length of hospital stay was reduced with a very early IS (3.9 days (SD 6.5) vs 6.3 days (SD 7.6), p<0.01).

**Conclusions:**

A strategy of very early IS did not improve clinical outcomes compared with a standard care IS in higher risk patients with NSTE-ACS. However, the primary outcome rate was low and the trial was underpowered to detect such a difference.

**Trial registration number:**

NCT03707314.

**WHAT IS ALREADY KNOWN ON THIS TOPIC**

- No significant difference in hard clinical outcomes has been demonstrated when an early invasive strategy is compared with a delayed invasive strategy in patients with high-risk non-ST-elevation acute coronary syndrome. There remains uncertainty regarding whether an early invasive strategy is of benefit in patients at higher baseline risk.

**WHAT THIS STUDY ADDS**

- RAPID NSTEMI is the largest randomised controlled trial to prospectively enrol higher risk patients as defined by Global Registry of Acute Coronary Events score criteria. The study was underpowered to detect a significant difference in the primary outcome. Event rates in this population were significantly lower than expected.

**HOW THIS STUDY MIGHT AFFECT RESEARCH, PRACTICE OR POLICY**

- The low event rates suggest that any potential treatment effect between early and delayed strategies may be so small that it is of questionable clinical significance. Future studies to investigate this area may be prohibited by trial design and cost.

## Introduction

The optimal timing of an invasive strategy (IS) in non-ST-elevation acute coronary syndrome (NSTE-ACS) remains uncertain. When randomised data are evaluated in totality, no difference in hard clinical outcomes between an early IS (defined as <24 hours) and delayed IS in NSTE-ACS all-comers has been demonstrated.^1 2^ However, uncertainty persists regarding those at highest baseline risk for future events.^2^ Subgroup analyses of patients from the Timing of Intervention in Acute Coronary Syndromes (TIMACS) and Very Early Versus Deferred Invasive Evaluation Using Computerised Tomography (VERDICT) trials with a Global Registry of Acute Coronary Events (GRACE) score of >140 suggest a reduction in composite ischaemic outcomes following an early IS.^3 4^

The proposed benefits of an early IS are that rapid identification and stabilisation of plaque rupture with percutaneous coronary intervention (PCI) will mitigate the risk of acute vessel occlusion, recurrent ischaemic events and extension of myocardial infarction (MI). Given the uncertainty regarding optimal timing of IS in higher risk NSTE-ACS, we designed the RAPID NSTEMI trial, a multicentre randomised controlled trial to determine if a very early IS was superior to standard care timing IS.

## Methods

### Trial design

The RAPID NSTEMI trial was an investigator-initiated, multicentre, randomised, controlled, pragmatic strategy trial undertaken at 30 PCI-capable hospitals in the UK. Full details regarding the study design have been published previously and are provided in the protocol and online supplemental material.^5^ RAPID NSTEMI was funded by the British Heart Foundation (grant number: CS/17/1/32445) and is registered at ClinicalTrials.gov (NCT03707314).

10.1136/heartjnl-2023-323513.supp1 Supplementary data

10.1136/heartjnl-2023-323513.supp2 Supplementary data

Eligible patients were required to have a clinical diagnosis of NSTE-ACS and symptoms of myocardial ischaemia within the prior 12 hours. Elevation of high-sensitivity troponin (hs-Tn) and GRACE 2.0 score of ≥118, or ≥90 with at least one high-risk feature (anterior ECG changes, ST-segment depression, diabetes mellitus on medication, hs-Tn elevation three times the upper limit of normal), were mandatory for trial inclusion.^6^ Major exclusion criteria were type 2 MI and need for urgent angiography according to European Society of Cardiology (ESC) guidelines (haemodynamic instability, recurrent or refractory chest pain, cardiogenic shock).^7^ Patients who met such criteria were then randomly assigned in a 1:1 ratio to undergo either a very early IS or a standard care timing IS via a secure centralised internet-based system. A complete list of inclusion and exclusion criteria is provided in the online supplemental material.

### GRACE 2.0 score

The GRACE 2.0 score has demonstrated superior discrimination to predict death and MI following acute coronary syndrome (ACS) as compared with the original GRACE model and has been externally validated in large observational cohorts.^6 8^ Rather than converting model estimates to a score, and using intervals for continuous variables such as age, the GRACE 2.0 score directly uses model estimates themselves to compute cumulative risk.^6^ In addition, a single score for risk of mortality at 6 months is created. A GRACE 2.0 risk score of ≥118 is essentially equivalent to a GRACE 1.0 score of >140, because both predict a 6-month mortality risk of greater than 6%. Patients at intermediate risk (GRACE 2.0 scores of ≥90 and <118) with higher risk features were included to attenuate the age bias of the GRACE score, thereby allowing enrolment of younger patients recognised to be at elevated risk of future major adverse cardiovascular events (MACE).^9–11^

### Trial procedures

Participants assigned to a very early IS were transferred to the catheter laboratory as soon as possible. Research teams were encouraged to achieve a randomisation to vascular sheath insertion time of <90 min. Enrolment at sites typically occurred during normal working catheter laboratory hours (08:00–18:00). Timing of standard care IS was according to typical practice at individual centres but encouraged to be <72 hours of admission to hospital, as per UK national guidelines.^12^ Clinical care, including PCI and coronary artery bypass grafting (CABG) surgery, was performed according to current international guidelines.^7 13^ Telephone follow-up was performed at 12 months.

### Endpoints

The primary endpoint was a composite of all-cause mortality, new MI or hospitalisation for heart failure (HHF) at 12 months. Key secondary outcomes included the individual components of the primary outcome, cardiovascular mortality, ischaemia-driven revascularisation, stroke, Bleeding Academic Research Consortium (BARC) 3–5 major bleeding^14^ and length of hospital stay. A complete list of secondary outcomes and definitions used are detailed in the online supplemental material. An independent clinical events committee, blinded to group allocation, adjudicated events that occurred during the trial.

### Statistical analysis

The primary hypothesis of RAPID NSTEMI was that a very early IS would result in a >25% relative risk reduction in the primary endpoint. Sample size calculations were based primarily on the subgroup analysis of GRACE >140 high-risk patients in the TIMACS trial, in which the composite primary endpoint of death, new MI and stroke at 6 months occurred in 21.0% of patients in the standard care arm.^3^ We included HHF since there is evidence of this being an important outcome following NSTE-ACS, with studies at the time of trial design reporting rates of up to 14% for HHF at 12 months after NSTE-ACS.^15^ Based on these data and use of the GRACE 2.0 score, the standard care timing IS arm composite event rate of all-cause mortality, new MI and HHF in RAPID NSTEMI was estimated to be 19% at 12 months. With a two-tailed type I error of 5%, power of 80% and the assumption of 5% withdrawal, 5% crossover and 8% requiring CABG, a recruitment target of 2314 was calculated. All patients were included in the final modified intention-to-treat analysis.

Descriptive statistics are presented for binary and categorical variables (numbers and percentages) and for continuous variables (mean and SD, or median and IQR, as appropriate). The analysis of primary and secondary outcomes compares treatment arms using a mixed effects logistic regression, adjusting for randomisation stratification factors of hospital site (as a random effect) and GRACE 2.0 score (as a fixed effect). Treatment comparison estimates are presented as adjusted ORs and 95% CIs. The primary outcome was also analysed in the prespecified subgroups of sex, age (<75 or ≥75 years), GRACE 2.0 score (≥90 and <118 or ≥118) and the presence or absence of ST-segment change on ECG. Time-to-first-event outcomes are measured from randomisation, and differences between treatment arms are compared using Cox proportional hazards models, with treatment comparisons presented as HRs and 95% CI, with models adjusted for the hospital site and GRACE 2.0 score.

### Patient and public involvement

The study was presented to the National Institute for Health Research University of Leicester Biomedical Research Centre patient and public involvement group. Development of the funding application, protocol, outcome measures and study conduct were discussed. Patient and public involvement representatives were members of the trial steering committee.

## Results

### Patients

**Figure 1:**
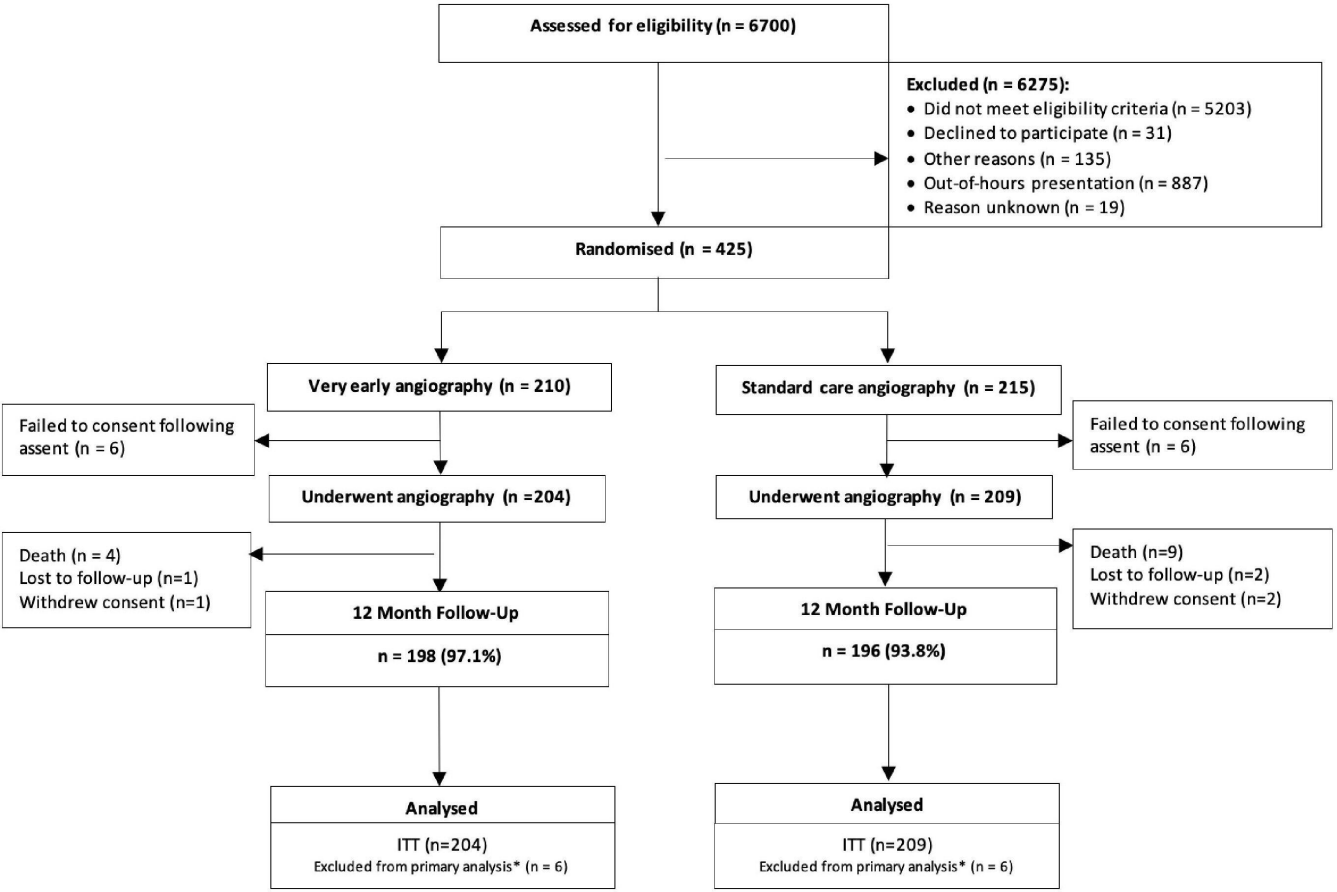
Study Consolidated Standards of Reporting Trials (CONSORT) diagram. *Intention-to-treat (ITT) population: all patients randomised into the trial except for patients who withdrew consent.

Of the 425 patients enrolled in the study from November 2018 to November 2020, two hundred and ten were randomly assigned to the early IS group and 215 to the standard care IS group (figure 1). In April 2021, the study was terminated by the funder because of slow recruitment due largely to the COVID-19 pandemic. Median follow-up was 12.0 months (IQR 11.3–12.3). Baseline characteristics were well matched across the groups, aside from a higher proportion of male patients in the standard care IS group (table 1). The mean age was 70.9 (SD 9.3) years, 26% had diabetes and the mean GRACE 2.0 score was 116.0 (SD 18.6).

**Table 1:** Baseline characteristics

	Very early IS (n=204)	Standard care IS (n=209)
Age, mean (SD)	70.7 (9.4)	71.1 (9.2)
Male sex, n (%)	126 (61.8)	167 (79.9)
Caucasian, n (%)	173 (84.8)	183 (87.6)
Current smoker, n (%)	40 (19.6)	34 (16.3)
Prior smoker, n (%)	69 (33.8)	87 (41.6)
Hypertension, n (%)	109 (53.4)	108 (51.7)
Diabetes mellitus, n (%)	50 (24.5)	51 (24.4)
Mean eGFR, mL/min/1.73m^2^ (SD)	75.1 (15.9)	73.5 (17.0)
Prior MI, n (%)	40 (19.6)	34 (16.3)
Prior PCI, n (%)	28 (13.7)	38 (18.18)
Ischaemia on ECG, n (%)	164 (81.6)	164 (79.2)
Elevated high-sensitivity troponin, n (%)	204 (100)	209 (100)
**Global Registry of Acute Coronary Events 2.0 score**		
Mean (SD)	115.0 (17.0)	117.0 (20.0)
≥118, n (%)	84 (41.2)	95 (45.4)
≥90 with at least one high-risk feature, n (%)	120 (58.8)	114 (54.6)
Anterior ECG changes, n (%)	25 (20.8)	17 (14.9)
ST-segment depression, n (%)	12 (10.0)	19 (16.7)
Diabetes mellitus on medication, n (%)	16 (13.3)	13 (11.4)
Elevated hs-Tn three times the ULN, n (%)	65 (54.2)	57 (50.0)

eGFR, estimated glomerular filtration rate; hs-Tn, high-sensitivity troponin; IS, invasive strategy; MI, myocardial infarction; PCI, percutaneous coronary intervention; ULN, upper limit of normal.

Overall, 97.1% of patients in the very early IS arm underwent invasive coronary angiography at median time from randomisation of 1.5 (IQR: 0.9–2.0) hours, as compared with 97.2% in the standard IS arm at 43.9 (IQR: 22.9–72.6) hours. Median time from admission to randomisation was 3.0 (IQR: 2.1–4.1) hours and 2.9 (IQR: 2.0–4.1) hours in the very early IS and standard IS groups, respectively (table 2). Unobstructed coronary arteries were identified in 21.8% of participants. Rates of PCI were slightly lower in the very early IS group (59.8%) as compared with the standard IS group (63.2%) (p=0.48). Complete revascularisation was higher in the very early IS (77.9% vs 68.9%).

**Table 2:** Procedural and angiographic characteristics

	Very early IS (n=204)	Standard care IS (n=209)
Median time from randomisation to angiography, hours (IQR)	1.5 (0.9–2.0)	43.9 (22.9–72.6)
Median time from admission to randomisation, hours (IQR)	3.0 (2.1–4.1)	2.9 (2.0–4.1)
Radial access, n (%)	189 (92.6)	189 (90.4)
**Angiographic characteristics**		
No coronary stenosis, n (%)	46 (22.6)	44 (21.0)
Left main coronary stenosis, n (%)	18 (8.8)	17 (8.1)
1-vessel disease, n (%)	68 (33.3)	67 (32.1)
2-vessel disease, n (%)	52 (25.5)	57 (27.3)
3-vessel disease, n (%)	38 (18.6)	41 (19.6)
≥1 occluded coronary artery, n (%)	43 (20.5)	39 (18.1)
Visible thrombus, n (%)	12 (5.9)	14 (6.7)
SYNTAX score, mean (SD)	14.80 (11.6)	16.36 (11.3)
**Coronary revascularisation after angiography**		
PCI, n (%)	122 (59.8)	132 (63.2)
CABG, n (%)	20 (9.8)	21 (10.0)
**PCI characteristics**		
≥1 drug-eluting stent, n (%)	115 (94.3)	126 (95.4)
Glycoprotein IIb/IIIa inhibitor used, n (%)	1 (0.8)	10 (7.6)
Complete revascularisation by PCI, n (%)	95 (77.9)	91 (68.9)
Number of stents, n (%)		
1	65 (53.3)	71 (53.8)
2	37 (30.3)	43 (32.6)
≥3	14 (11.5)	14 (10.6)
PCI success, n (%)	117 (95.9)	128 (97.0)

CABG, coronary artery bypass grafting; IS, invasive strategy; PCI, percutaneous coronary intervention; SYNTAX, Synergy between Percutaneous Coronary Intervention with Taxus and Cardiac Surgery.

### Primary and secondary outcomes

**Figure 2:**
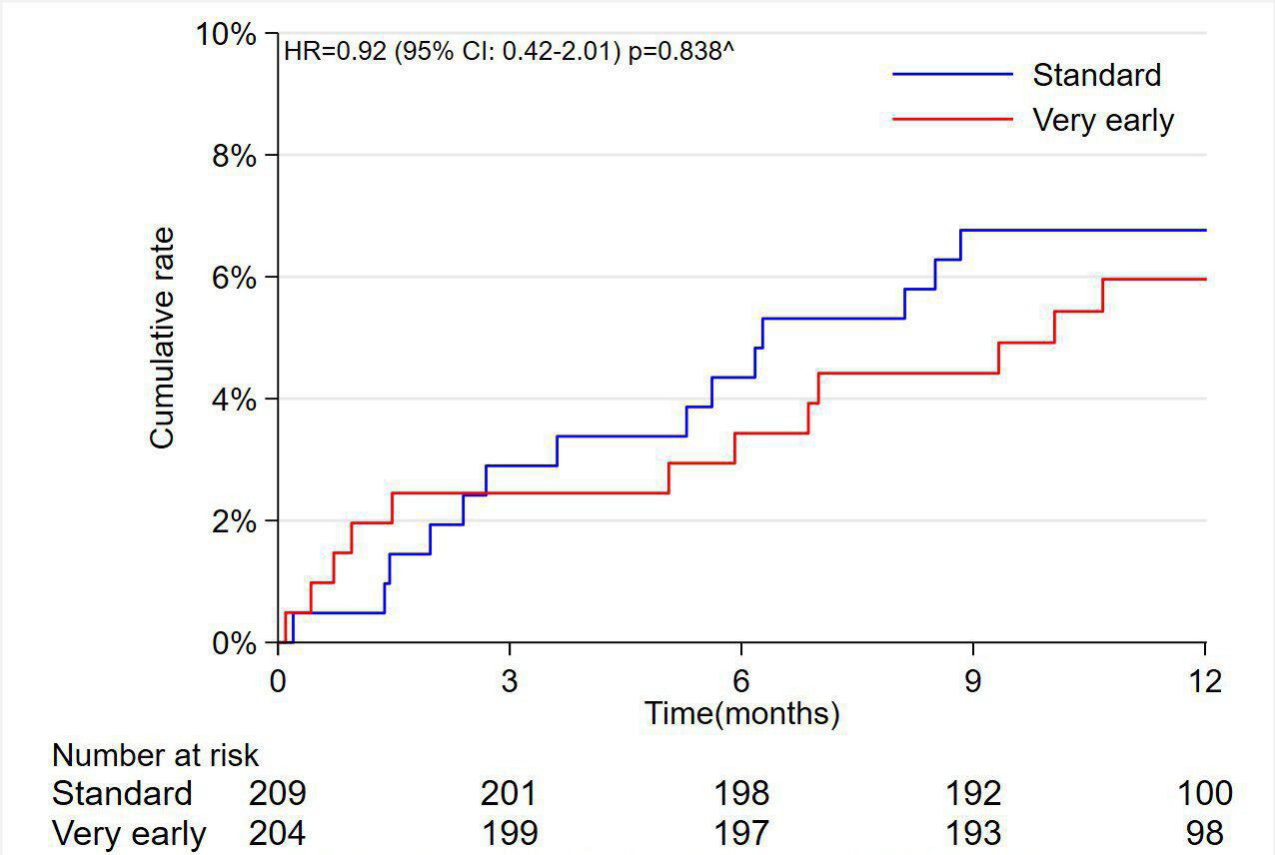
Kaplan-Meier cumulative event rates for the composite primary endpoint. ∧ Model adjusted for the hospital site and Global Registry of Acute Coronary Events (GRACE) 2.0 score.

At 12 months, the incidence of the primary outcome of all-cause mortality, new MI and HHF was 5.9% in the very early IS group as compared with 6.7% in the standard IS group (OR 0.93, 95% CI 0.42 to 2.09; p=0.86) (table 3, figure 2).

**Table 3:** Primary and secondary outcomes

	Very early IS (n=204)	Standard care IS (n=209)	OR (95% CI)	P value
**Primary outcome, n (%)**				
All-cause mortality, new MI and hospitalisation for heart failure	12 (5.9)	14 (6.7)	0.93 (0.42 to 2.09)	0.86
**Secondary outcomes, n (%)**				
All-cause mortality	4 (2.0)	9 (4.3)	0.50 (0.15 to 1.67)	0.26
Cardiovascular mortality	3 (1.5)	1 (0.5)	6.02 (0.47 to 77.85)	0.17
New MI	6 (2.9)	7 (3.4)	0.88 (0.29 to 2.68)	0.82
CV mortality or new MI	8 (3.9)	7 (3.4)	1.23 (0.43 to 3.47)	0.70
Hospitalisation for heart failure	5 (2.4)	3 (1.4)	1.99 (0.45 to 8.69)	0.36
Stroke	2 (1.0)	3 (1.4)	0.70 (0.12 to 4.28)	0.70
BARC 3–5 major bleeding	6 (2.9)	2 (1.0)	3.45 (0.67 to 17.61)	0.14
Major VARC-2 access site complications	3 (1.5)	0 (0.0)	–	–
Emergent angiography while awaiting procedure	0 (0.0)	15 (7.2)	–	–
**Length of hospital stay, mean (SD)**				
Length of hospital stay, days	3.9 (6.5)	6.3 (7.6)	−2.36 (−3.74 to −0.98)*	<0.01

Statistical models were adjusted for randomisation stratification factors of hospital site (as a random effect) and Global Registry of Acute Coronary Events (GRACE) 2.0 score (as a fixed effect).

*Adjusted mean difference (95% CIs).

BARC, Bleeding Academic Research Consortium; CV, cardiovascular; IS, invasive strategy; MI, myocardial infarction; VARC-2, Vascular Academic Research Consortium 2.

There was no significance difference between the very early IS and standard IS in the rate of all-cause mortality (2.0% vs 4.3%), cardiovascular death (1.5% vs 0.5%), new MI (2.9% vs 3.4%) and HHF (2.5% vs 1.4%) (table 3). Of note, 7.2% of patients assigned to the standard IS group required emergent angiography due to clinical deterioration while awaiting cardiac catheterisation.

### Safety outcomes

Stroke occurred in 1.0% of patients in the very early IS group, as compared with 1.4% in the standard IS group (OR 0.70, 95% CI 0.12 to 4.28; p=0.70) (table 3). There was no significant difference between the arms with respect to the rate of BARC 3–5 major bleeding (2.9% vs 1.0%; OR 3.45, 95% CI 0.67 to 17.61; p=0.14). Three Vascular Academic Research Consortium-2-defined major access site complications were observed patients who underwent a very early IS, as compared with zero in patients who underwent a standard timing IS.

### Length of hospital stay

The length of hospital stay was shorter in the very early IS group (mean 3.9 days; SD 6.5) as compared with the standard IS group (mean 6.3 days; SD 7.6) with an adjusted mean difference of −2.4 days (95% CI −3.74 to −0.98; p<0.01).

### Prespecified subgroups

**Figure 3:**
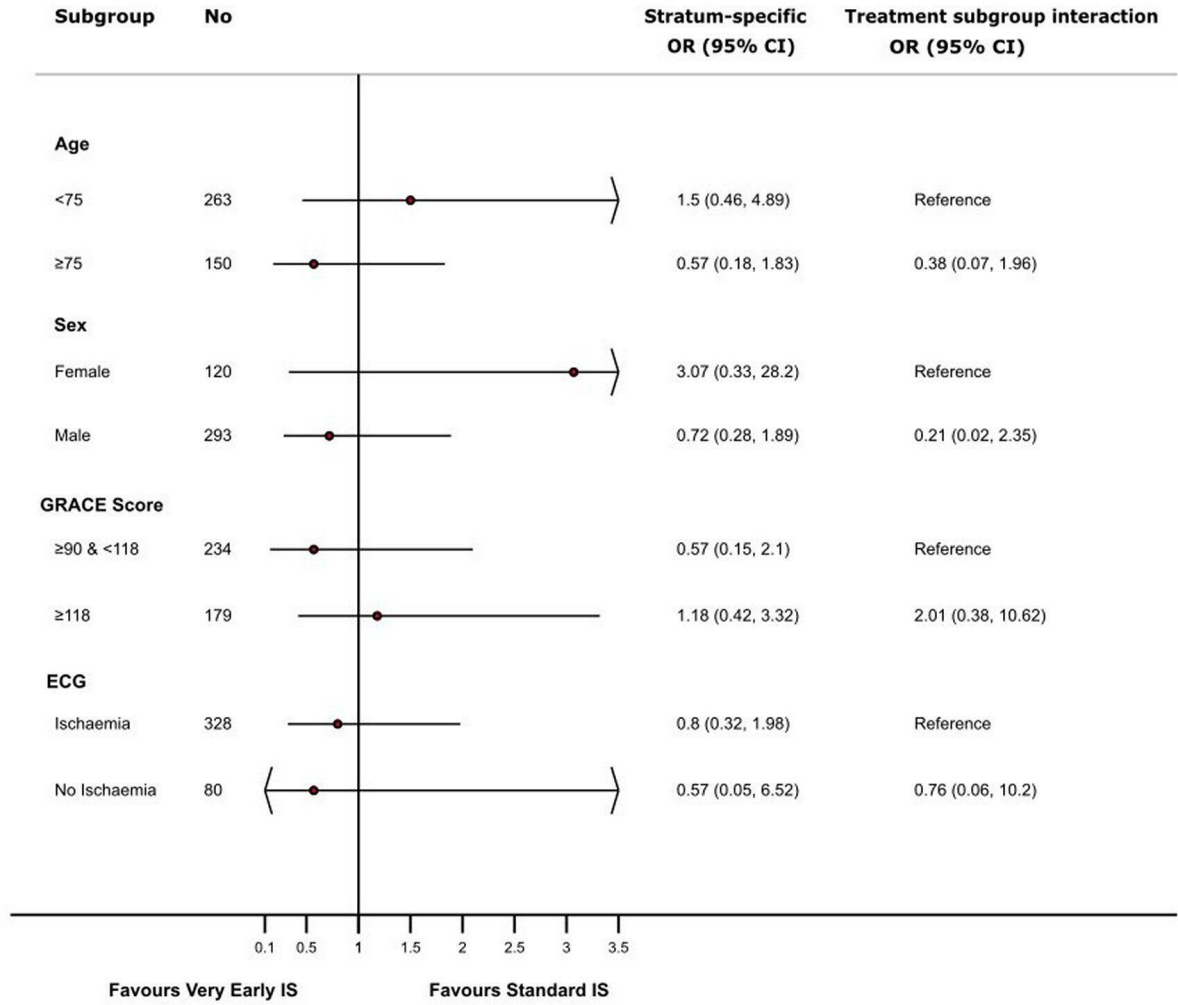
Prespecified subgroup analyses of the composite primary endpoint. Adjusted OR of composite of all-cause mortality, new myocardial infarction and admission for heart failure at 12 months. Models adjusted for the hospital site and Global Registry of Acute Coronary Events (GRACE) 2.0 score. IS, invasive strategy.

No significant interaction was observed between a very early IS and age (<75 or ≥75 years) (p=0.25), sex (p=0.21), GRACE 2.0 score (p=0.41) or new ischaemia on ECG (p=0.83) (figure 3).

## Discussion

In this multicentre, randomised clinical strategy trial of a very early IS compared with standard care IS among patients with NSTE-ACS at higher baseline risk, no significant difference was observed with respect to the primary composite endpoint of all-cause mortality, new MI or HHF. There was also no significant difference in the rates of stroke and major bleeding between the two study arms. Length of hospital stay was reduced by a mean of 2.4 days in the very early IS arm. However, due to the premature termination of the study and low rate of the primary outcome, the trial was underpowered to demonstrate a significant difference, with wide CIs that cannot exclude an effect size that encompasses a 58% reduction or a 109% increase in the primary endpoint. Thus, the results should not be considered definitive.

There remains uncertainty regarding the optimal timing of IS in patients with NSTE-ACS, particularly in those at higher risk for future ischaemic events. Prior ESC and current American Heart Association/American College of Cardiology guidelines advocate that an early IS (<24 hours) should be undertaken in those patients at highest baseline risk.^7 16^ However, the present iteration of the 2023 ESC ACS guidelines has downgraded this recommendation from IA to IIaA following the publication of a study-level meta-analysis from the RAPID NSTEMI investigators that demonstrated only a difference in rates of recurrent ischaemia between the two strategies in NSTE-ACS all-comers.^1 17^ Recommendations to consider an early IS are primarily based on the GRACE >140 subgroup analyses from the TIMACS and VERDICT trials, which demonstrated a reduction in composite ischaemic outcomes following an early IS.^3 4^ Both studies provide the majority of patients included in patient-level meta-analyses that suggest a reduction in all-cause mortality associated with an early IS in patients with NSTE-ACS at high baseline risk.^2 18^ Such findings should, however, be interpreted in the context of their limitations. Each trial predates the adoption of hs-Tn assays, potent antiplatelet agents and improved guideline-directed medical therapy. Moreover, these GRACE >140 subgroup analyses should be considered hypothesis generating, because the primary outcomes in both overall TIMACS and VERDICT trial populations were neutral. The more recent Early or Delayed Revascularisation for Intermediate and High-risk Non ST-elevation Acute Coronary Syndromes (EARLY) trial tested a very early IS (median 0.0 hour) versus a delayed IS (median 18.0 hours) in 741 patients with ESC-defined intermediate or high-risk NSTE-ACS. However, the mean GRACE score was 122 and hence confirmed this was a population at intermediate risk of future clinical events. Furthermore, the reduction in the composite endpoint of cardiovascular death and recurrent ischaemia at 30 days in the very early IS group was driven by a reduction in recurrent ischaemia (a single 10 min episode of chest pain was sufficient to meet this outcome). No significant difference in death or MI was apparent.^19^

The RAPID NSTEMI trial was established to test a strategy of very early IS in a higher risk population as defined by the updated GRACE 2.0 score, who were managed according to accepted contemporary practice. Herein, the median time to angiography (1.5 hours from randomisation) was accelerated when compared with the early arms in the TIMACS (median 14.0 hours) and VERDICT (median 4.7 hours) trials. Furthermore, RAPID NSTEMI participants were randomised very soon after presentation to hospital (median 3.0 hours). These data have not been reported in prior studies and delays to randomisation may be a limitation in the robust testing of an early IS. The mean age in RAPID NSTEMI was 71 years, whereas TIMACS, VERDICT and EARLY enrolled patients with a mean age of 65, 64 and 65, respectively.^3 4 19^ All patients in RAPID NSTEMI exhibited cardiac biomarker elevation, similar to EARLY, but increased as compared with TIMACS (77%) and VERDICT (80%) populations.

Although underpowered because of early termination due to slow enrolment, it should be acknowledged that despite the high baseline risk of the enrolled population, clinical event rates observed were very low. The 12-month incidence of death for the total cohort in RAPID NSTEMI was 3%, significantly less even when compared with all-comer NSTE-ACS populations in the TIMACS (5%) and Study of Platelet Inhibition and Patient Outcomes (PLATO) trials (5%), for instance.^3 20^ Continued improvement in clinical outcomes following NSTE-ACS has been observed over time,^21^ while the advent of improved guideline-directed medical therapy and contemporary clinical care has reduced HHF and provided a high bar to demonstrate significant reduction in MACE between differing therapeutic strategies.^22^ Given our observed event rate, with a point estimate of 6.7% in the standard care arm, over 7400 individuals would have been required for RAPID NSTEMI to have 80% power to detect a 25% reduction at the 5% significance level if other sample size assumptions remain unchanged. This is prohibitive in terms of trial design and cost, and such a number suggests that any potential treatment effect between strategies may be so small that it is of questionable clinical significance.

Many centres across Europe do not meet current ESC guideline recommendations with regard to timing of IS in higher risk patients with NSTE-ACS.^23 24^ The reasons for this are likely twofold. First, if using ESC guideline criteria, most patients admitted to hospitals are defined as ‘high-risk’ due to cardiac biomarker elevation. A retrospective analysis from the UK showed that 94% of patients with NSTE-ACS meet this definition, yet only 16% receive an IS within 24 hours.^23^ Considerable restructuring of pathways would be necessary in many countries to achieve an early IS because many healthcare systems do not have the requisite catheter laboratory capacity and/or staffing resource. Second, the data to support improved clinical outcomes following an early IS in high-risk patients are lacking. Until now, there has been an absence of a randomised clinical trial that prospectively and specifically investigated a high-risk NSTE-ACS population. RAPID NSTEMI was the first such trial that attempted to test a very early IS in a higher risk population as defined by the GRACE score.

Importantly, no hazard was observed when a very early IS was undertaken in this older, higher risk patient cohort. Specifically, rates of stroke and major bleeding were similar and are consistent with meta-analyses that have concluded an early IS does not carry excess risk.^1^ Given the absence of safety concerns, and low likelihood of significant difference in clinical outcomes between very early and delayed IS, attention should focus on the potential economic savings for healthcare systems associated with an early IS. In RAPID NSTEMI, we demonstrated a significant mean reduction in length of stay of 2.4 days when a very early IS was undertaken and compared with a delayed IS. Similar positive results associated with an early IS were reported in the TIMACS (−2.0 days) and EARLY (−0.6 days) trials.^3 19^ Moreover, the TIMACS investigators published health economic analyses that concluded that an early IS was likely to be less costly than a delayed approach, but this finding was limited by their inability to capture all costs.^25^ More robust healthcare cost-efficacy data in the setting of a contemporary study are therefore required to inform any potential change in conventional practice.

Limitations of RAPID NSTEMI should be considered. First, the trial was prematurely terminated due to slow enrolment having reached 18% of its original recruitment target. The trial is therefore markedly underpowered to detect a difference in the primary endpoint. Second, a mean GRACE 2.0 score of 116 suggests that a larger proportion of intermediate-risk patients with higher risk features were enrolled and, in part, explains the low event rate. Third, a high ratio of screened to randomised patients was observed. This was due to restrictive trial inclusion criteria and may impact external validity of RAPID NSTEMI; however, our aim was to enrich the trial population with higher risk patients, a group that would be expected to benefit most from a very early IS and subsequent revascularisation. Use of the selected GRACE 2.0 risk score thresholds for trial inclusion excludes between 50% and 70% of lower risk patients with NSTE-ACS and largely explains this ratio.^3 4^ Fourth, a higher than expected proportion of patients with non-obstructive coronary arteries (22%) were enrolled in the trial. This is likely due to basing the diagnosis of NSTE-ACS on a single hs-Tn elevation. This cohort likely represents patients with type 2 MI and therefore dilutes any possible treatment effect associated with an early IS.

## Conclusions

In patients with NSTE-ACS at high baseline risk, there were no significant differences in clinical outcomes between a very early or standard care IS; however, the trial was underpowered to detect such a difference.

## Data Availability

Data are available upon reasonable request. Anonymised patient-level data are available on reasonable request from the corresponding author. The full trial protocol is available on request from the corresponding author. The protocol has been published previously.
