# Supplementary material for: Very early invasive strategy in higher risk non-ST-elevation acute coronary syndrome: the RAPID NSTEMI trial
Source: Heart. 2023 Dec 16;110(7):500–7. doi: 10.1136/heartjnl-2023-323513 (PMC10958296; doi:10.1136/heartjnl-2023-323513)
Supplement: Supplementary data [file heartjnl-2023-323513supp001.pdf]

Supplementary Appendix

Trial Sites and Investigators

Study Coordination

S1: Inclusion and exclusion criteria

S2: Pre-specified secondary endpoints

S3: Clinical outcome events definitions

S4: Cardiac-related medications at baseline and follow up

S5: Kaplan-Meier curves for the individual endpoints of all-cause mortality (A), new myocardial infarction (B), and hospitalisation for heart failure (C).

Trial Sites and Investigators:

Site Name	Site Investigators
**Glenfield Hospital, Leicester**	Anthony Gershlick<br>Andrew Ladwiniec<br>Thomas Kite
**Leeds General Infirmary**	John Greenwood
**University Hospital Southampton**	Nick Curzen
**Queen Alexandra Hospital, Portsmouth**	Brijesh Anantharam
**Blackpool Victoria Hospital**	Ranjit More
**Kettering General Hospital**	Simon Hetherington
**Queen Elizabeth Hospital, Birmingham**	Sohail Khan
**Royal Bournemouth Hospital**	Peter O'Kane
**John Radcliffe Hospital, Oxford**	Adrian Banning
**Royal Free Hospital, London**	Roby Rakhit
**New Cross Hospital, Wolverhampton**	James Cotton
**Morriston Hospital, Swansea**	Alexander Chase
**Royal Stoke University Hospital**	James Nolan
**Birmingham City Hospital**	Vinoda Sharma
**Northwick Park Hospital, London**	Ahmed Elghamaz
**Manchester Royal Infirmary**	Freidoon Keshvarzi
**University Hospital of Wales, Cardiff**	Andrew Sharp
**Royal Sussex County Hospital, Brighton**	David Hildick-Smith
**Kings College, London**	Jonathan Byrne
**Royal Cornwall Hospital, Truro**	Tamas Ungvari
**Worthing Hospital**	Sujay Chandran
**University Hospital Coventry & Warwickshire**	Martin Been

**St George's Hospital, London**	Sami Firoozi
**Lincoln County Hospital**	David O'Brien
**Nottingham City Hospital**	Akhlaque Uddin
**William Harvey Hospital, East Kent**	Paula Mota
**Torbay Hospital, Torquay**	Dirk Felmeden
**Craigavon Area Hospital**	Michael Moore
**Altnagelvin Area Hospital**	James Shand
**Royal Albert Edward Infirmary, Wigan**	Ayyaz Sultan

Study Coordination

Trial Steering Committee: Nicholas Boon (Chair)**,** Keith Oldroyd, Jennifer Adgey

Study Sponsor: University Hospitals of Leicester NHS Trust

Clinical Trials Unit: Leicester Clinical Trials Unit, University of Leicester

Data Safety Monitoring Board: Bernard Gersh (Chair), Stephan Windecker, Huon Gray, Stuart Pocock

Clinical Events Committee: Andreas Baumbach (Chair), Divaka Perera, Scott Garg

S1

Inclusion criteria

- Age ≥18 years
- Patients with a clinical diagnosis of non-ST elevation myocardial infarction comprising:
    - Symptoms of myocardial ischaemia
    - Elevated high sensitivity troponin T or I
- GRACE 2.0 score of either:
    - ≥118 OR
    - ≥90 but <118
  - If GRACE 2.0 score ≥90 or <118 must have at least <u>one</u> additional high-risk feature:
    - Anterior location of ECG changes (leads V2 – V5)
    - ST-segment depression in 2 contiguous leads (any territory) of 0.15mV/1.5mm
    - Diabetes mellitus on medication
    - High-sensitivity troponin I or T 3x upper limit of normal
- Ischaemic symptoms within 12 hours prior to hospital admission
- Intention to perform angiography and, if indicated, follow-on revascularisation
- Provision of assent or written consent

Exclusion criteria

- ST elevation myocardial infarction
- Evident type 2 myocardial infarction
- Evidence of previous known cardiomyopathy
- Cardiogenic shock
- Severe valvular heart disease
- Need for urgent PCI according to European Society of Cardiology guidelines (e.g., haemodynamic instability, recurrent or persistent pain)
- Any contraindication to PCI
- Current participation in another intervention trial

S2

Pre-specified secondary endpoints

- All-cause mortality at 12 months
- New myocardial infarction at 12 months
- Hospitalisation for heart failure at 12 months
- Cardiovascular mortality at 12 months
- Cardiovascular mortality or new MI at 12 months
- Length of inpatient hospital stay (defined as randomisation to discharge)
- Stroke at 12 months
- BARC 3-5 major bleeding as inpatient, and at 12 months
- Admission for ischaemia-driven revascularisation at 12 months
- Admission for any cause at 12 months
- VARC-2 access site complications as inpatient, and at 12 months
- Major VARC-2 access site complications as inpatient, and at 12 months
- Events prior to planned procedure:
    - All-cause mortality prior to coronary angiography
    - New myocardial infarction prior to coronary angiography
    - BARC 3-5 major bleeding prior to coronary angiography
- Quality of life measured using Seattle Angina Questionnaire at 24 hours post procedure, 1 month, 6 months and 12 months
- Quality of life measured using the EQ-5D-5L questionnaire at 24 hours post procedure, 1 month, 6 months and 12 months
- Cost effectiveness of early invasive strategy versus standard care timing invasive strategy
- Proportion of patients in standard care timing IS arm that require emergent/urgent angiography

S3

Clinical outcome events definitions

New myocardial infarction

Defined as according to the Fourth Universal Definition of Myocardial Infarction.[1]

Hospitalisation for heart failure

Defined as a hospital admission with any of the following symptoms and signs: worsening breathlessness, fatigue, fluid overload, pulmonary oedema, elevated venous pressure and elevated beta natriuretic peptide.

Stroke

The presence of a new focal neurologic deficit thought to be vascular in origin, with signs or symptoms lasting more than 24 hours.

Major bleeding

Defined according to the Bleeding Academic Research Consortium (BARC) consensus document. Major bleeding is defined as BARC type 3-5.[2]

Vascular access complications

Defined according to the Vascular Access Research Consortium 2 (VARC-2) consensus document.[3]

Ischaemia-driven revascularisation

Defined as repeat intervention with either percutaneous coronary intervention (PCI) or coronary artery bypass grafting (CABG) for new symptoms or signs of myocardial ischaemia.

Complete revascularisation

Defined as intervention on all vessels >2.25mm with at least one stenosis >50%.

S4

Cardiac-related medications at baseline

**Medication, n (%)**	**Very early angiography (n=204)**	**Standard care angiography (n=209)**
Aspirin	106 (52.0%)	107 (51.0%)
P2Y12 inhibitor	78 (38.2%)	77 (36.8%)
Clopidogrel	28 (13.7%)	29 (13.9%)
Ticagrelor	48 (23.5%)	46 (22.0%)
Prasugrel	2 (1.0%)	2 (1.0%)
Warfarin	3 (1.5%)	5 (2.4%)
Direct oral anticoagulants	10 (4.9%)	9 (4.3%)
Beta blocker	57 (27.9%)	60 (28.7%)
Calcium channel blocker	44 (21.6%)	46 (22.0%)
Nitrate	35 (17.2%)	34 (16.3%)
ACE inhibitor	55 (27.0%)	61 (29.2%)
Angiotensin-II blocker	30 (14.7%)	31 (14.8%)
Diuretic	26 (12.8%)	31 (14.8%)
Statin	95 (46.6%)	100 (47.8%)

S5

Kaplan-Meier curves for the individual endpoints of all-cause mortality (A), new myocardial infarction (B), and hospitalisation for heart failure (C).

A

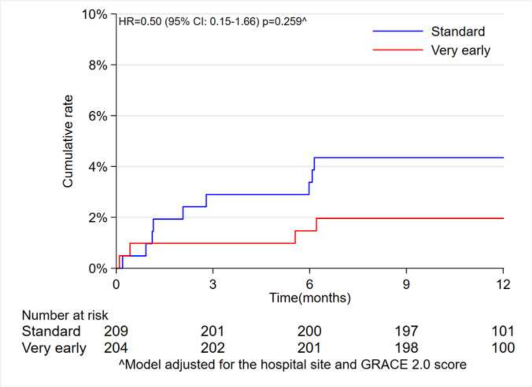

B

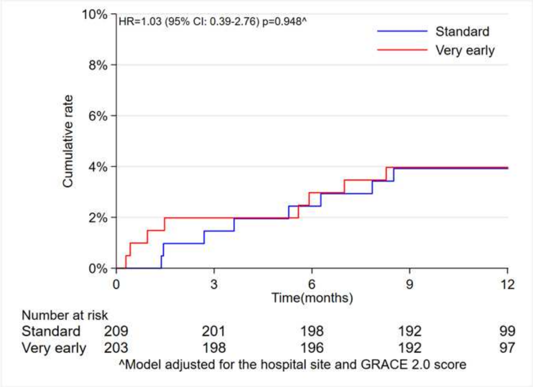

C

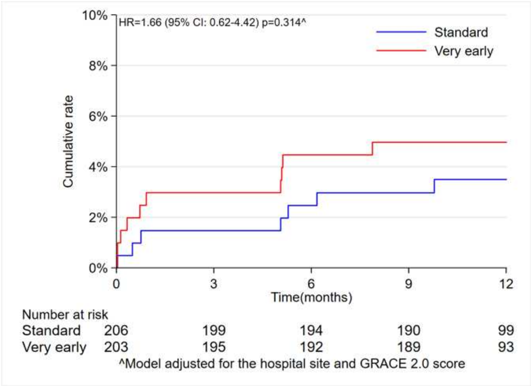

References

1. Thygesen K, Alpert JS, Jaffe AS*, et al.* Fourth universal definition of myocardial infarction (2018). *Eur Heart J* 2018. doi: 10.1093/eurheartj/ehy462

2. Mehran R, Rao SV, Bhatt DL*, et al.* Standardized bleeding definitions for cardiovascular clinical trials: a consensus report from the Bleeding Academic Research Consortium. *Circulation* 2011;**123**:2736-2747. doi: 10.1161/CIRCULATIONAHA.110.009449

3. Kappetein AP, Head SJ, Genereux P*, et al.* Updated standardized endpoint definitions for transcatheter aortic valve implantation: the Valve Academic Research Consortium-2 consensus document. *J Am Coll Cardiol* 2012;**60**:1438-1454. doi: 10.1016/j.jacc.2012.09.001
