# Supplementary material for: Very early invasive strategy in higher risk non-ST-elevation acute coronary syndrome: the RAPID NSTEMI trial
Source: Heart. 2023 Dec 16;110(7):500–7. doi: 10.1136/heartjnl-2023-323513 (PMC10958296; doi:10.1136/heartjnl-2023-323513)
Supplement: Supplementary data [file heartjnl-2023-323513supp002.pdf]

Contents

1.0 ABBREVIATIONS 6
2.0 TRIAL SUMMARY 7
3.0 FLOW DIAGRAM 9
4.0 SCIENTIFIC ABSTRACT 10
5.0 LAY ABSTRACT 11
6.0 INTRODUCTION 11
6.1 THE IMPACT OF THE GRACE SCORE 12
6.2 SCIENTIFIC PRINCIPLES UNDERLYING THE PROPOSED RESEARCH 13
6.3 JUSTIFICATION FOR THE STUDY 13
6.4 EXPECTED VALUE OF RESULTS 14
6.5 HYPOTHESIS TO BE TESTED 14
7.0 STUDY DESIGN 16
7.1 AIMS OF THE STUDY 16
7.2 OUTCOME MEASURES (for definitions see APPENDIX II) 16
7.2.1 Primary clinical outcome 16
7.2.2 Secondary Outcome Measures 16
7.3 ELIGIBILITY 17
7.3.1 Inclusion Criteria 17
7.3.2 Exclusion Criteria 17
7.4 TRIAL INTERVENTIONS 19
7.5 CALCULATION OF THE GRACE RISK SCORE 20
7.6 RECRUITMENT 20
7.7 RANDOMISATION 20
7.8 INTERVENTION 21
Table 1: Troponin & EQ5D-5L 22
7.9 LOGISTICS 22
TABLE 2: SUMMARY OF BASELINE, RANDOMISATION AND FOLLOW-UP PROCEDURES 24
7.10 IN-PATIENT MANAGEMENT GUIDELINES 25
7.11 PROCEDURES AT DISCHARGE 26
7.12 CARDIAC MAGNETIC RESONANCE (APPENDIX V) 26
7.13 BIOMARKERS SUBSTUDY (APPENDIX VI) 26
7.14 FOLLOW-UP 26
7.15 CLINICAL EVENT REPORTING 27
7.15.1 General Potential Risks and Hazards to patients 27
7.15.2 ADVERSE EVENTS 28
7.15.3 Definitions of Adverse Events 28
7.15.4 Definitions of Serious Adverse Events (SAE) 28
7.15.5 Expected serious adverse events/clinical outcomes 28
7.15.6 Classifying SAEs 29
7.16 SAE REPORTING 29
7.17 END OF TRIAL 29

Kite TA, *et al. Heart* 2024; 110:500–507. doi: 10.1136/heartjnl-2023-323513

7.18 WITHDRAWAL 29
8.0 STATISTICAL ASPECTS 30
8.1 Power calculations 30
8.2 STATISTICAL ANALYSIS PLAN 31
8.3 HEALTH ECONOMIC ANALYSIS 31
9.0 DATA MANAGEMENT 33
9.1 DATA ENTRY 33
9.2 DATA PROTECTION 33
9.3 CONFIDENTIALITY 33
9.4 DATA ACCESS 33
9.5 ARCHIVING 33
10.0 TRIAL ORGANISATION, REGULATION AND OVERSIGHT 34
10.1 FUNDING SOURCES 34
10.2 ETHICAL CONSIDERATIONS 34
10.3 SPONSOR 34
10.4 HEALTH RESEARCH AUTHORITY (HRA) APPROVAL 34
10.5 TRIAL REGISTRATION 34
10.6 INSURANCE AND INDEMNITY 34
10.7 TRIAL MANAGEMENT GROUP (TMG) 34
10.8 TRIAL STEERING COMMITTEE (TSC) 35
10.9 DATA SAFETY MONITORING COMMITTEE (DSMC) 35
10.10 CLINICAL EVENTS COMMITTEE (CEC) 35
10.11 TRIAL CO-ORDINATION 35
10.12 HEALTH ECONOMICS ANALYSIS 35
10.13 CONSORT 35
10.14 DISSEMINATION & PUBLICATION POLICY 36
10.15 COMPETING INTERESTS 36
11.0 TRIAL TIMETABLE 36
APPENDICES 37
APPENDIX I: GRACE SCORE 37
APPENDIX II: CLINICAL EVENT DEFINITIONS 39
APPENDIX III: Symptoms of ischaemia 42
APPENDIX IV: Recommended Secondary Prevention Therapy 43
APPENDIX V: CMR SUBSTUDY 45
APPENDIX VI: BIOMARKERS SUBSTUDY 50
APPENDIX VII Risks to recruitment 64
APPENDIX VIII Tables of recent protocol changes 65
SA_02, 01 August 2019 65
REFERENCES 68

1.0 ABBREVIATIONS

ACS	Acute coronary syndrome
BARC	Bleeding Academic Research Consortium
CCS	Canadian Cardiovascular Society
CMR (I)	Cardiac Magnetic Resonance (Imaging)
CEC	Clinical Events Committee
CKMB	Creatine kinase myocardial brain
CABG	Coronary artery bypass grafting
DAPT	Dual Antiplatelet Therapy
DSMC	Data Safety Monitoring Committee
ECG	Electrocardiogram
eCRF	Electronic case report form
FBC	Full blood count
FFR	Fractional Flow Reserve
GCP	Good Clinical Practice
HRA	Health Research Authority
Hs-TnI	High sensitivity Troponin I
Hs-TnT	High sensitivity Troponin T
IDMC	Independent Data Monitoring Committee
IVRS	Interactive Voice Response System
LBBB	Left Bundle Branch Block
LCTU	Leicester Clinical Trials Unit
LGE	Late Gadolinium Enhancement
LMWH	Low-molecular-weight heparin
MRIS	Medical Research Information System
MVT	Multi-vessel treatment
MI	Myocardial infarction
MPS	Myocardial Perfusion Scan
MVD	Multi-vessel disease
N- STEMI ACS	Non-ST elevation myocardial infarction acute coronary syndrome
N-IRA	Non-infarct related (coronary) artery
PPCI	Primary percutaneous coronary intervention
PCI	Percutaneous coronary intervention
RCT	Randomised Controlled Trial
SAQ	Seattle Angina Questionnaire
STEMI	ST elevation myocardial infarction
TSC	Trial Steering Committee
U&Es	Urea and Electrolytes
UoL	University of Leicester
UHL	University Hospitals of Leicester NHS Trust

Kite TA, *et al. Heart* 2024; 110:500–507. doi: 10.1136/heartjnl-2023-323513

2.0 TRIAL SUMMARY

**Title**	A randomised controlled trial of very early angiography +/- intervention versus standard of care on outcomes in patients with non ST-elevation myocardial infarction.
**Acronym**	RapidNSTEMI
**Study Design**	Prospective open multicentre randomised controlled trial in patients with higher risk non ST elevation myocardial infarction acute coronary syndrome
**Randomised controlled trial (RCT) Eligibility**	**Inclusion Criteria**<br>● 18 years of age and over<br>● Patients presenting to hospitals with a clinical diagnosis of non-ST elevation myocardial infarction comprising:<br>○ Ischaemic symptoms (as defined in Appendix III)<br>○ Elevated high sensitivity Troponin T or I (above the normal range for individual hospitals)<br>● GRACE-2.0 score (www.gracescore.org) of either:<br>○ ≥118 (corresponding to 6-month death >6%) OR<br>○ ≥90 but <118 (corresponding to 6-month death >3% but <6%)<br>● If GRACE 2.0 score ≥90 or <118 must have at least <u>one</u> additional high risk feature:<br>○ Anterior location of ECG changes (leads V2 – V5)<br>○ ST-segment depression in 2 contiguous leads (any territory) of 0.15mV/ 1.5mm.<br>○ Diabetes Mellitus on medication<br>○ High-sensitivity Troponin I or T 3 x ULN<br>● Onset of ischaemic symptoms at any time prior to admission <u>but most recent episode within 12hrs prior to admission</u><br>● Intention to perform angiography and, if indicated, follow-on revascularisation<br>● Provision of assent or written consent<br>● Randomisation must be performed within 6 hours of admission<br><br>**Exclusion Criteria**<br>● ST elevation myocardial infarction<br>● Evident type 2 myocardial infarction (e.g. anaemia)<br>● Evidence of previous known cardiomyopathy<br>● Cardiogenic Shock<br>● Known severe valvular heart disease<br>● Need for urgent PCI according to ESC Guidelines (haemodynamic instability, VT, VF, recurrent or persistent pain)<br>● Any contraindication to PCI<br>● Current participation in another intervention trial
**Randomisation**	*Group A:* Immediate angiography with follow-on revascularisation if required<br>*Group B:* Standard care – pharmacological treatment until angiography with follow-on revascularisation if required (preferably within 72hrs).
**Recruitment sample size Sub-studies and Follow up**	● >25 UK sites to recruit 2314 patients over 2 years<br>● Clinical follow-up at 30 days (telephone), 6 months (telephone) and 12 month (telephone or clinic visit)<br>● Health Economic analysis<br>● Cardiac Magnetic Resonance Imaging (CMRI) sub-study (chosen sites)

Kite TA, *et al. Heart* 2024; 110:500–507. doi: 10.1136/heartjnl-2023-323513

	● Biomarkers sub-study (chosen sites)<br>● 10 year data capture via NHS digital to assess long term outcomes*<br>● Screening log of all patients<br><br>* This will not be included in the main study database as it is routinely collected data. The main study database will be locked and the rest of the outcomes will be analysed before this outcome's data collected has been completed.
**Primary Outcome Measure**	Incidence of the composite of **all-cause mortality, new myocardial infarction and admission for heart failure** within **12 months** following randomisation.

Kite TA, *et al. Heart* 2024; 110:500–507. doi: 10.1136/heartjnl-2023-323513

3.0 FLOW DIAGRAM

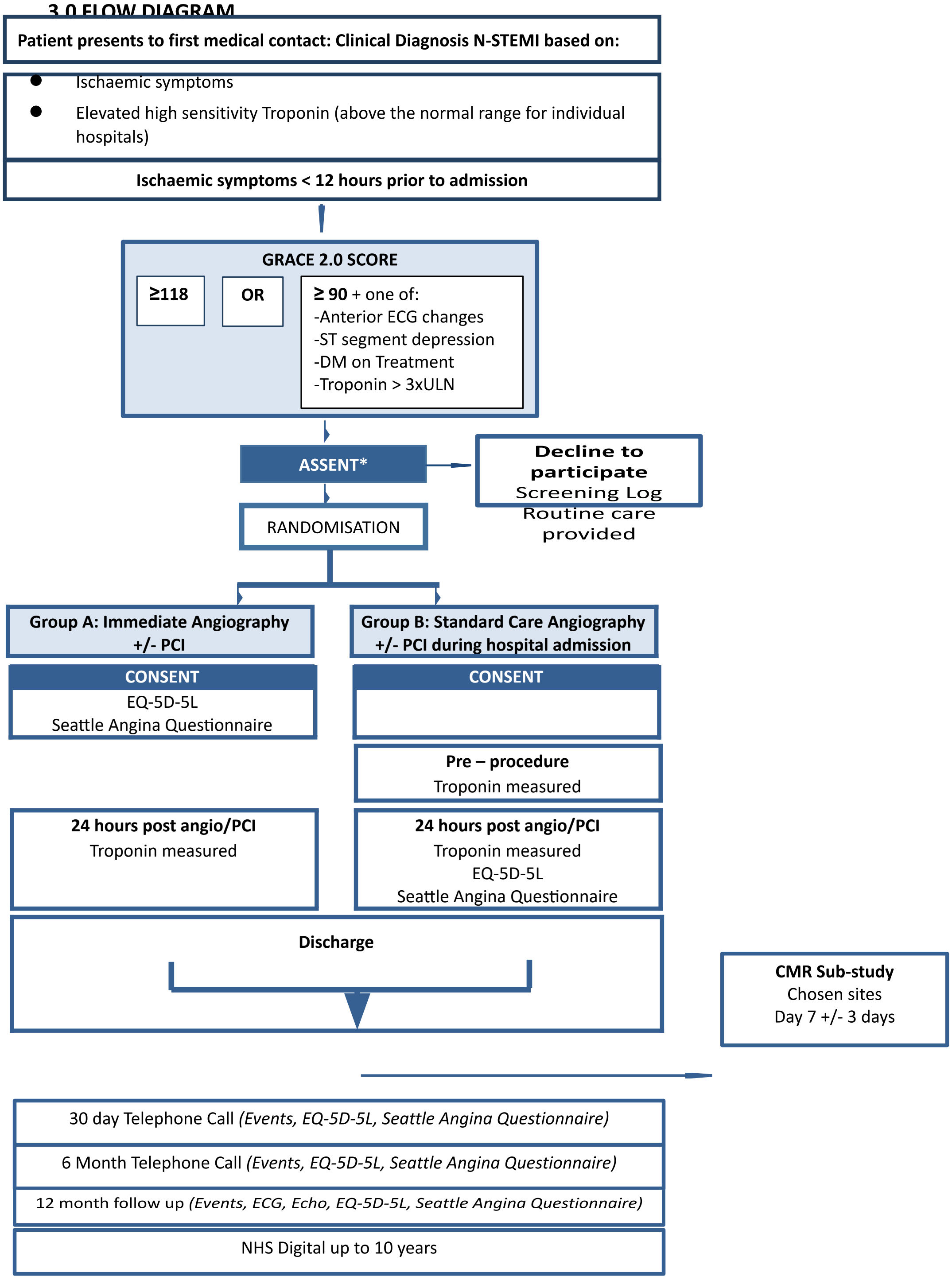

Primary Outcome at 12 months: All-cause mortality, new myocardial infarction and heart failure requiring hospital admission

*or written consent, depending on local regulatory guidelines (applicable to sites in Northern Ireland/Scotland)

4.0 SCIENTIFIC ABSTRACT

Background: Clinical event rates in non-ST elevation myocardial infarction acute coronary (N-STEMI ACS) patients remain high, with one year MACE rate as high as 20% (Stefan James personal communication Swedish SCARR registry), and while there may be early mortality differences between N-STEMI and STEMI, outcomes beyond one year become very similar. N-STEMI ACS patients therefore, rightly remain the focus of a number of research directives. The objective of the RAPID-NSTEMI trial is to determine if clinical outcomes can be improved by very early intervention in a pre-determined higher risk N-STEMI ACS population. Published data has shown that in-patient Percutaneous Coronary Intervention (PCI) in N-STEMI ACS patients reduces subsequent clinical events, and this had led to Guidelines support for its use. However, there is much less certainty regarding the timing of the PCI and, in particular, whether this should be a strategy used early to optimize outcomes. Thus, while evidence based Guidelines (NICE and European) provide general time parameters for PCI, immediate angiography with a view to intervention in higher risk patients has never been robustly tested in any adequately powered, prospective randomised trial with clinical end points. The RAPID-NSTEMI trial sets out to test the benefits, or otherwise of a strategy of immediate angiography with follow-on revascularisation in higher risk N-STEMI ACS patients.

Hypothesis: higher risk N-STEMI ACS patients do better if treated earlier than current standard of care. Currently such patients are not specifically looked for.

Methods: In order to identify higher risk patients as soon as possible after presentation we will measure troponin release on admission, allowing us to calculate a GRACE score (GS) early after admission. By measuring High-sensitivity Troponin (Hs-Troponin-T or Hs-Troponin-I) we will determine the GS in sufficient time to be able to test an early intervention strategy arm. Patients with GS 2.0 of ≥118 alone, or ≥90 with additional high risk features will be randomised in a 1:1 fashion to one of two groups:

Group A: immediate angiography with follow-on revascularisation if required

Group B: standard care – pharmacological treatment until angiography with follow on revascularisation if required (preferably within 72 hours as per current Guidelines).

The primary outcome for the main study will be a 12-month composite of Major Adverse Cardiovascular Events (MACE) consisting: overall mortality/new MI/admission with heart failure.

Power calculations indicate that 2314 patients are required to show MACE superiority for early intervention in such higher risk N-STEMI ACS patients.

Analyses will be primarily according to “intention to treat”, with a secondary analysis according to trial treatment received (comparing those who actually received follow-on revascularisation at the two different trial time points). There will be a cost effectiveness analysis.

We will undertake a mechanistic CMR sub-study and biomarkers sub-study in the two groups. Expected value of results: We have designed a superiority trial to anticipate that the outcomes will be improved in higher risk patients revascularised very early after presentation. Irrespective of outcome, this trial should determine whether there is a need for a change in current patient management of a common condition and in particular if all N-STEMI patients should be admitted to a PCI-capable hospital to allow for very early intervention. The results will inform National/International Guidelines, and guide healthcare

service provision. The planned cost effectiveness analysis will become particularly important if clinical outcomes are no different between groups since length of stay should be different.

5.0 LAY ABSTRACT

Heart attacks are common. They may be major or minor. Smaller heart attacks occur when a blood clot forms on narrowing's in the coronary arteries and then cause partial blockage of the artery supplying blood to the heart. If they are not treated appropriately then the artery may block completely which can result in poorer outcomes (such as major heart attack or death). Published Guidelines tell us that these small heart attacks need to be treated. Such patients are therefore admitted to hospital, given drugs to prevent more clot forming, and within 3-4 days, are taken to the catheter laboratory (a special X ray room) where any narrowing's in the coronary arteries can be seen on the X ray screen and treated, where necessary, with a balloon and stent (angioplasty).

We are able to detect that there has been a heart attack by the release of small amounts of protein from injured heart muscle – these proteins are known as "troponins" and they can be measured using a blood test. Recently, it has been possible to detect small amounts of troponins in the blood very early after admission in patients with small heart attacks. Hence, we are now able to determine how severe the heart attack is according to the blood levels of early measured troponins. If we put these measured levels into an app programme together with some patient details such as age and kidney function, we come up with a score that tells us what the future risk to the patient is. If the calculated score is high, this suggests it may be better to treat this type of patient much earlier with balloon and stent than is currently undertaken, although this has never been proven. Alternatively, patients may be better left for longer on the medical treatment until the balloon and stent treatment. In this study we are therefore comparing the strategy of going to the cath lab early and treating if necessary as compared with going to the cath lab according to current standard timings.

The outcomes that we will measure to test whether very early balloon and stent in higher risk patients is actually beneficial, will be: death, further heart attack or heart failure occurring over the subsequent 12 months of follow up. We will also undertake a special cardiac heart scan called an MRI to see if the heart attack is smaller with earlier balloon and stent, and measure levels of circulating proteins in the blood called biomarkers.

6.0 INTRODUCTION

N-STEMI ACS is a condition that results from atheromatous plaque erosion/rupture and the subsequent formation of peri-plaque embolic thrombus that is not occlusive, but may become so (and so lead to STEMI).The management of such patients involves administration of potent anti-platelet agents (P_2Y_{12} blockers) and anti-thrombins such as low molecule weight heparin (LMWH) (1–5) in order to attenuate the formation of any evolving platelet-centric thrombus formation. The TACTICS -TIMI 18 (6), FRISC II (7) and RITA-3 (8) trials together with meta-analyses (9–11) strongly indicate that N-STEMI ACS patients benefit from in-patient coronary intervention. PCI has thus become a Guideline recommendation as part of N-STEMI ACS management (12). However, the actual timing of the PCI to provide best clinical outcomes is less clear. Whether there is a benefit from a period of several days of medical therapy that includes antiplatelet agents and high dose statins and which could theoretically result in plaque passivation and reduced clotting tendency, is a possibility that has never been prospectively and robustly tested. Although a number of studies have attempted to provide evidence for the best timing of in-hospital PCI intervention and especially whether there is any benefit in very early intervention, all are

somewhat flawed. For example so-called “early” PCI in some trials was not actually early at all: in the ICTUS (13,14) and TACTICS (6) trials, "early" PCI intervention was undertaken at a median time of 23 and 24 hours respectively, and in FRISC-II and RITA-3 trials (7,8) ”early” PCI was actually very late indeed (median 96hrs). Furthermore, interpretation of trial outcomes is confounded by differing trial logistics, including trial inclusion criteria. Only 3 trials (see below) assessed really early intervention – of these, one was driven by non-clinical end points and another small study showed adverse outcomes in early intervention, contrary to all other trials. Specifically, in the ABOARD study (15) patients were randomised to immediate PCI or intervention the following day, but the primary end point was enzyme-determined infarct size, not clinical outcomes. Additionally, the lack of risk stratification could account for the demonstrated lack of difference even in the non-clinical endpoint. The OPTIMA study (16) compared immediate PCI versus PCI delayed for 24-48 hours and the incidence of 6 month primary end point (death, non-fatal MI, unplanned revascularisation) was significantly higher in the early group (intervention at median 25 minutes from randomisation) compared to those in the delayed (25 hour) group (RR=1.5, 95% CI=1.10-2.07, p=0.008). This small study (n=140) suggests a case could be made for so-called “pharmacological passivation” prior to PCI. However, nearly all of the end-point difference occurred in the acute stages, suggesting perhaps it was acute events (i.e. PCI related events) that drove any differences. In the LIPSIA-NSTEMI-ACS study there was no significant difference between immediate (within 2hr) and early (within 24hrs) PCI in higher-risk N-STEMI ACS patients with GRACE score >140. However, again this study was not powered to detect differences in clinical events; only differences in CK-MB (17). The best study, the RIDDLE N-STEMI trial, randomised 323 N-STEMI ACS patients to receive either immediate (<2h from randomisation) or delayed (2-72hr, median time in trial 61hr) PCI (18). This relatively small trial showed 30-day primary endpoint of death or new MI was lower in the immediate PCI group (4.3% vs. 13%, HR=0.32, 95% CI=0.17-0.67, p=0.002). This difference was driven by re-infarction in the delayed PCI group while awaiting angiography. There was no difference in the composite endpoint in a landmark analysis from 31days-1yr. Again, this trial was underpowered to detect new MI post-discharge and also to detect any impact on death up to 1 year post N-STEMI ACS. As with all of the studies quoted above, GRACE score was not used as a criteria for enrolment and only 36% of the study population retrospectively had a GRACE score >140.

In a meta-analysis by Kastrati (19) no benefit in hard end points (death/AMI) as a result of early intervention was demonstrated, although there was a reduction in recurrent ischaemia within the early revascularisation group (RR=0.57, 95% CI=0.44 – 0.74, p<0.001). A more recent meta-analysis (20) of 7 RCTs (5370 patients) and 4 observational studies, showed, in the total 77,499 patients, that there was no conclusive evidence of a survival benefit from very early intervention (in the randomised studies). However, the range of "early" intervention was 0.5-14hrs, and for delayed intervention was 20–86hrs post-randomisation. A third meta-analysis suggests early routine invasive strategy appears to reduce the 5-year risk of the combined endpoint of cardiovascular death and myocardial infarction, with separate reductions in the risk of myocardial infarction and cardiovascular mortality (21).

In conclusion, the currently available data are discrepant and not of sufficient scientific quality to accurately inform the best management of this very large group of patients.

6.1 THE IMPACT OF THE GRACE SCORE

In a post hoc subgroup analysis of the TIMACS study, those patients who had a GRACE score of >140 were shown to benefit from early intervention with significant improvement in

outcomes (HR=0.65, 95% CI=0.48-0.88, p=0.005) (22). Hence, a larger prospective and appropriately powered trial in N-STEMI ACS patient is required to detect any real differences from very early intervention. In many studies the early intervention was not early and the delayed intervention occurred beyond the guideline recommended 72hrs. Patients have not been stratified as higher risk by GRACE score and trial numbers have not allowed for assessment of any strategy on clinical end points. Currently no consensus on whether immediate PCI leads improves outcomes can been formed, due to conflicting, and contrary reporting data. The TIMACS post hoc GRACE score analysis suggests the cut off should be >140 but this was GRACE score version 1.0. As such it is likely that to use this cut off will only identify those patients determined by GRACE score to be highest risk according mostly to demographics such as age. We have taken a more subtle approach using GRACE score 2.0 (APPENDIX I) to identify a higher risk population according to % event rates at 6 and 12 months.

6.2 SCIENTIFIC PRINCIPLES UNDERLYING THE PROPOSED RESEARCH

Clinical presentation and outcomes of N-STEMI ACS reflect a process of incomplete occlusion of the coronary artery by platelet aggregation and thrombus formation at the site of an eroded/ruptured atherosclerotic plaque. While intuitively the case could be made for immediate PCI to prevent complete occlusion by establishing optimal flow (in accordance with Virchow's principle) it can be argued that the use of anti-platelet and anti-coagulant agents, as well as high dose statins, for a period of time could be beneficial. This should theoretically allow any disrupted plaque to become stabilised, so promoting autolysis, and hence attenuate the presence of clot and so reduce risk of PCI induced embolisation at early PCI (Type 4a MI). Delay to allow for so-called passivation may also reduce the risk of true "no reflow" phenomenon, and decrease risk of microvascular obstruction. On the other hand, the recent introduction of more potent P_2Y_{12} inhibitors (Ticagrelor or Prasugrel) will lead to earlier and more effective platelet inhibition over a shorter time period prior to early PCI, perhaps negating the need for a period of time to passivate the vessel wall.

Earlier identification of higher risk N-STEMI ACS patients is now possible through the introduction of Hs-Troponins, which allow early assessment of degree of myocardial injury and so patient risk, and thereby allow for earlier decisions around use of an interventional strategy.

The RAPID-NSTEMI trial, which utilizes these principles, has been designed to determine whether early risk assessment and early intervention improves outcome. This study would aim to provide a robust definitive answer to the question whether immediate revascularisation in higher risk N-STEMI patients confers benefit.

6.3 JUSTIFICATION FOR THE STUDY

All trials on timing of intervention in N-STEMI ACS to date have varied in their statistical design, choice of end points and patient inclusion criteria, and some also appear methodologically flawed, for e.g. in ISAR-COOL a different definition of MI was applied to the two trial groups. All trials testing so-called true very early intervention have been under-powered for clinical outcomes. In addition, randomisation in most trials was undertaken following measurement of standard troponins, introducing an inevitable delay before randomisation could take place, limiting their ability to robustly test true "early" intervention. The question therefore remains open, making RAPID-NSTEMI a novel study. Contemporary Hs-Troponins allow detection of N-STEMI within 1-2 hrs of admission as opposed to the 8-12hrs for standard Troponins, thus allowing earlier identification of the

higher risk N-STEMI ACS patients (23,24). The diagnostic sensitivity of a two hour rule-in protocol for ACS using Hs-Troponin-I was demonstrated to be in the range 95%-97% with specificity ranging between 78%-82% (25,26) used Hs-Troponin-T and demonstrated sensitivity of 96%-99% with a positive predictive values of 78%-85%.

If the immediate admission Hs-Troponin is raised above the normal range for that hospital, then the GRACE score 2.0 will be calculated at that point. We will know within 2-3 hours post-admission (accounting for 1hr for the result to become available) which of the patients has a GRACE score that allows them to be deemed "higher risk" and so included in the study. We believe use of the GRACE score is justified (>300 publications, >20,000 patients), and this is consistent with current NICE guidelines for risk stratification of ACS patients. We will use the adapted GRACE 2.0 score (where there is no need for knowledge of the creatinine or Killip grade). We have debated at length the value of the score cut-off and believe the following to be correct for the potential beneficial impact of very early intervention in N-STEMI patients **– Inclusion criteria = GRACE-2 score ≥118, or ≥90 with additional high risk features.** These levels are based on in depth review of the GRACE 2.0 score outcomes and the desire to impact sufficient numbers of N-STEMI ACS patients.

We will randomise these higher risk patients at an early time point to immediate angiography with a view to follow-on revascularisation (i.e. go to the cath lab ASAP with no additional avoidable delay – so-called “STEMI-like treatment”) or to revascularisation according to standard care – intervention preferably within 72 hours as per ESC Guidelines. https://www.escardio.org/Guidelines/Clinical-Practice-Guidelines
Irrespective of result RAPID-NSTEMI will focus clinicians on need for early evaluation and assessment of patients presenting with ACS to determine which patients should be managed earlier.

6.4 EXPECTED VALUE OF RESULTS

RAPID-NSTEMI will answer an important clinical question: can outcomes in higher risk N-STEMI ACS patients be improved with immediate PCI in higher risk populations, compared to current standard care? This trial will inform clinical practice and International Guideline Committees, regardless of the result. We may be able to rationalise the ACS service and determine the impact of any results on service provision and so redirect clinician’s focus on risk assessment. For example, whilst cardiac networks exist for primary-PCI for STEMI, given the incidence of N-STEMI ACS, such networks may need to be enhanced if clinical benefit were demonstrated with this study. This balance between determining what is best for patients, and potentially over burdening already stretched ACS networks will be a vital consideration but can only be resolved if studies such as RAPID-NSTEMI suggest significant clinical benefit, or not. This question as to best timing of intervention, be it immediate or as current, somewhat delayed (see BCIS data below) is unanswered. Relative to their numbers and important clinical outcomes, N-STEMI ACS patients are an under-studied group.

6.5 HYPOTHESIS TO BE TESTED

RAPID-NSTEMI will test the following hypothesis:
Immediate angiography with a view to follow on revascularisation in patients presenting with N-STEMI ACS and at higher risk (GRACE score ≥118 or ≥90 with additional high risk features), will result in a significant reduction in rate of major adverse clinical outcomes

(death/MI/hospitalisation for heart failure) within 12 months compared with angiography with follow on revascularisation undertaken according to current standard of care.

Randomisation will take place prior to angiography, following GRACE 2.0 score assessment.
At angiography it will become clear that a percentage of patients will require CABG (~8%), some will have normal coronary arteries and some will be treated medically but the analysis will be on an intention to treat (ITT) basis, since randomisation needs for logistical reasons to be undertaken prior to angiography. While the final analysis will be ITT, outcomes according to actual treatment received will also be reported separately as a “per treatment received” analysis.
This will be very important since it will test the hypothesis as to whether very early received intervention is better than current standard of care. Thus, a secondary analysis will be those who receive revascularisation at the time of very early angiography versus those who receive revascularisation at the timing of current standard of care.

We expect this to be a Guideline-informing, clinical practice-directing trial that will help direct clinicians as to the timing of PCI in N-STEMI ACS patients. The trial is didactic, simple, robust, and will be managed by experienced interventional trialists and a registered Clinical Trials Unit, all with a track record for trial delivery.

Kite TA, *et al. Heart* 2024; 110:500–507. doi: 10.1136/heartjnl-2023-323513

7.0 STUDY DESIGN

This will be a prospective open multicentre randomised controlled trial in patients with higher risk non ST elevation myocardial infarction acute coronary syndrome.

7.1 AIMS OF THE STUDY

This study will aim to answer the following questions:

- In N-STEMI ACS patients who are shown to be at higher-risk (GRACE score ≥118 or ≥90 with additional high risk features), does immediate angiography with a view to follow on revascularisation significantly improve outcomes (efficacy and safety) compared to angiography with follow on revascularisation at routinely used timing?
- Will any benefits correlate with degree of estimated risk (i.e. will the highest GRACE 2.0 score patient benefit most)?
- Is an early intervention strategy cost effective?
- Can mechanistic understanding be gained from CMR sub-study?
- Can novel biomarkers predict need for PCI and future clinical outcomes?
- Will longer term "passive" follow up through NHS Digital show an improvement in outcomes?

7.2 OUTCOME MEASURES (for definitions see APPENDIX II)

7.2.1 Primary clinical outcome

Incidence of the composite of **all-cause mortality, new myocardial infarction and admission for heart failure** within **12 months** following randomisation.

7.2.2 Secondary Outcome Measures

- Individual components of the primary end-point within 12 months:
  - Incidence of all-cause mortality within 12 months
  - Incidence of new myocardial infarction within 12 months
  - Incidence of admission for heart failure within 12 months
- Incidence of cardiovascular mortality within 12 months
- Length of in-patient stay (defined as randomisation to first discharge) in days
- Events prior to planned procedure:
  - Incidence of all-cause mortality prior to planned coronary angiography following index admission with NSTEMI
  - Incidence of new myocardial infarction prior to planned coronary angiography following index admission with NSTEMI
  - Incidence of major bleeding (classified as BARC 3-5) prior to planned coronary angiography following index admission with NSTEMI
- Incidence of admission for ischaemia-driven revascularisation within 12 months
- Incidence of admission for any cause within 12 months
- Quality of life measured using Seattle angina score at 24 hours post procedure, 1 month, 6 months and 12 months
- Quality of life as assessed using the EQ-5D-5L questionnaire at 24 hours post procedure, 1 month, 6 months and 12 months
- Incidence of BARC 3-5 classified bleeding as in-patient, and up to 12 months
- Incidence of stroke within 12 months

- Cost effectiveness of immediate PCI versus standard care
- Left ventricular ejection fraction (LVEF) and infarct size on cardiac MRI scan*
- Sensitivity and specificity of novel biomarkers in predicting which patients do or do not require PCI following diagnostic angiography**
- Proportion of patients needing emergency/urgent revascularisation (in group B) Incidence of total VARC-2 classified access site complications as in-patient, and up to 12 months
- Incidence of major VARC-2 classified access site complications as in-patient, and up to 12 months

*Additional CMR outcomes are specified in the CMR substudy protocol (appendix V)
** Additional biomarker outcomes are specified in the Biomarkers substudy protocol (appendix VI)

7.3 ELIGIBILITY

7.3.1 Inclusion Criteria

- 18 years of age and over
- Patients presenting to hospitals with a clinical diagnosis of non-ST elevation myocardial infarction comprising:
  - Ischaemic symptoms (as defined in Appendix III)
  - Elevated high sensitivity Troponin T or I (above the normal range for individual hospitals)
- GRACE-2.0 score (www.gracescore.org) of either:
  - ≥118 (corresponding to 6-month death >6%) OR
  - ≥90 but <118 (corresponding to 6-month death >3% but <6%)
- If GRACE 2.0 score ≥90 or <118 must have at least one additional high risk feature:
  - Anterior location of ECG changes (leads V2 – V5)
  - ST-segment depression in 2 contiguous leads (any territory) of 0.15mV/ 1.5mm
  - Diabetes Mellitus on medication
  - High-sensitivity Troponin I or T 3 x ULN
- Onset of ischaemic symptoms at any time prior to admission but most recent episode within 12 hours prior to admission
- Intention to perform angiography and, if indicated, follow-on revascularisation
- Provision of assent or written consent
- Randomisation must be performed within 6 hours of admission

7.3.2 Exclusion Criteria

- ST elevation myocardial infarction
- Evident type 2 myocardial infarction (e.g. anaemia)
- Evidence of previous known cardiomyopathy
- Cardiogenic Shock
- Known severe valvular heart disease
- Need for urgent PCI according to ESC Guidelines (haemodynamic instability, VT,VF, recurrent or persistent pain)
- Any contraindication to PCI
- Current participation in another intervention trial

Kite TA, *et al. Heart* 2024; 110:500–507. doi: 10.1136/heartjnl-2023-323513

RAPID-NSTEMI

Figure 2: Patient Flow to Randomisation

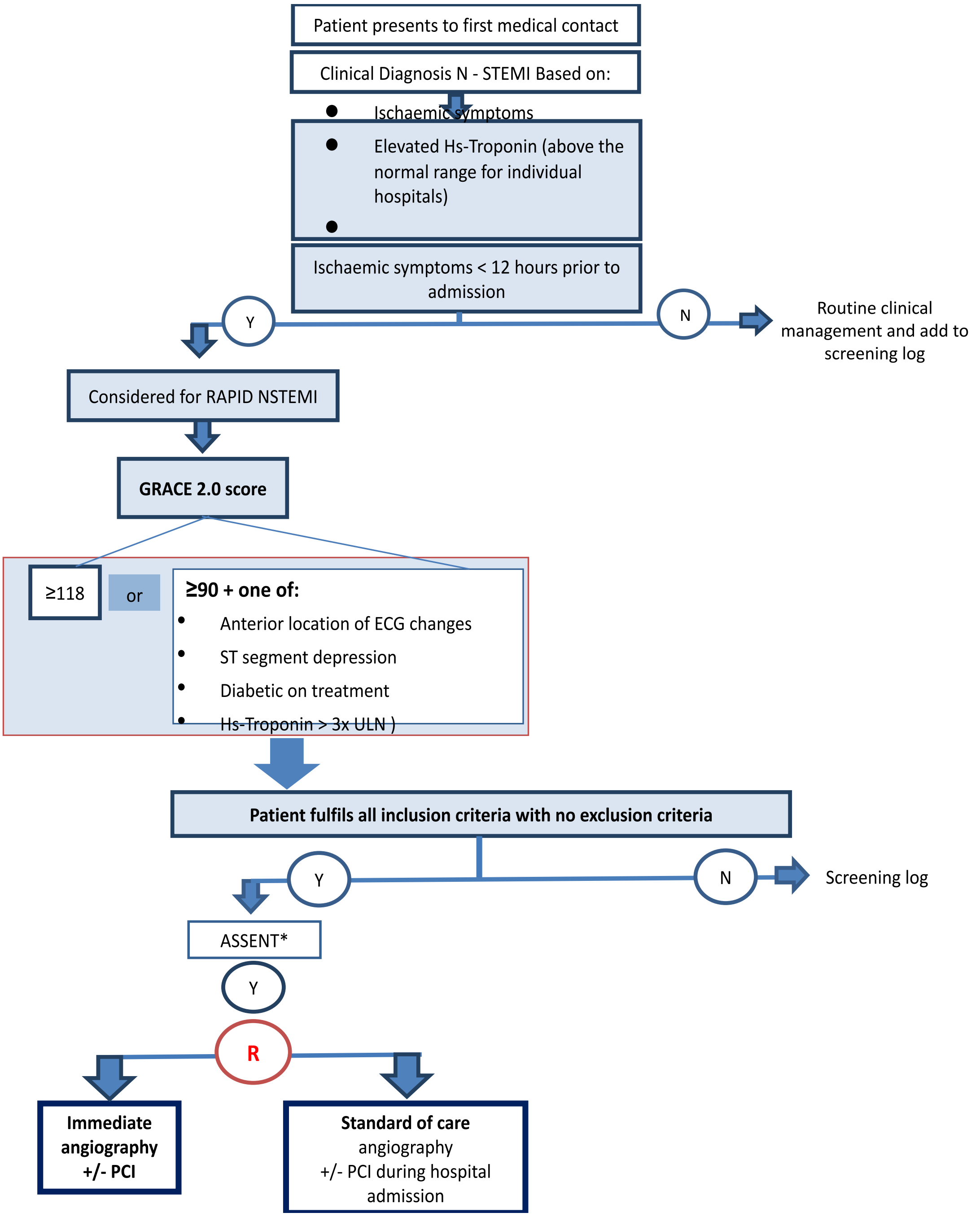

*or written consent, depending on local regulatory guidelines (applicable to sites in Northern Ireland/Scotland only)

Kite TA, et al. Heart 2024; 110:500–507. doi: 10.1136/heartjnl-2023-323513

7.4 TRIAL INTERVENTIONS

If the initial clinical eligibility for N-STEMI criteria are suspected, blood is drawn for Hs-Troponin immediately (i.e. as close to admission as possible). If this returns raised according to individual hospitals criteria then a GRACE 2.0 score is calculated at that time. A GRACE 2.0 score of ≥118, or ≥90 with additional high risk features - see inclusion criteria, will lead to patient ASSENT (or written informed consent, depending on local regulatory guidelines. Further details are provided in section 7.6) and if confirmed then randomisation providing there are no exclusion criteria. If the initial Hs-Troponin is reported as normal, a second sample may be taken at 3 hours in line with current European guidelines. If elevated, the participant may be randomised if all other criteria met. Randomisation will be performed using an independent Interactive Voice Response System randomisation system (Sealed Envelope Ltd), the patient will be randomised to either Group A or B. The participant trial number will be documented.

GROUP A: Immediate angiography with follow-on revascularisation if indicated - transfer to the catheter laboratory should be performed as soon as possible, and is recommended but not mandated within 90 minutes of randomisation

GROUP B: Standard angiography with follow on revascularisation if indicated, - within Guideline recommended times.
Standard interim therapy such as LMWH/DAPT will be mandated in this group until angiography.

All interventional procedures will be undertaken according to current best practice (e.g. peri-procedural intravenous heparin, radial approach as preferred access site, and use of drug-eluting stents). Patients in Group B who suffer the following:-

- on-going chest pain,
- recurrent dynamic ECG changes
- haemodynamic instability during the wait till angiography

will be allowed to cross to earlier angiography +/- revascularisation as per best clinical practice. Reasons for cross-over will be carefully documented. Crossover rates will be carefully monitored for accuracy but if crossover occurs, this will be measured as a secondary end-point.

We have considered carefully whether this design will allow sufficient temporal separation between the two groups enabling robust interpretation of the results. According to the 2015 BCIS audit returns, the median time to angiography for patients (directly) admitted to a PCI-capable centre with N-STEMI ACS = 60.8 hours, and for inter-hospital transfer = 82.1 hrs. Apart for one outlier the earliest time for the direct admission patients appears to be in the order of 26 hours. The risk of time contamination between groups A and B will be minimised by strict trial discipline; without this discipline the study will be difficult to interpret and will not be robust. The "times to intervention" will be important trial audit measures. All recruiting sites will be asked to treat the standard care arm participants according to their

NHS Trust's current practice. To reduce time delay in Group A only trial sites with on-site cath labs will be included.

All patients will be included in a within-trial cost effectiveness analysis.

A mechanistic CMR sub-study of 200 patients (100 from each group) will evaluate LV function, and differences in infarct size (especially the impact of Type 4a MIs) between groups (APPENDIX V).

A biomarkers substudy will investigate if novel biomarkers can predict the need for PCI and future clinical outcomes (APPENDIX VI)

7.5 CALCULATION OF THE GRACE RISK SCORE

The GRACE score will be calculated in accordance with current practice. The calculator is freely available electronically and may be downloaded as an app on most hospitals computers or smartphones. We will provide appropriate apps for smart phones and devices if needed. Since GRACE 1.0 inadequately captures all higher risk features it has been decided to include all patients with a GRACE 2.0 score of ≥118 but also to include those with a GRACE 2.0 score between 90 and 118 if they have other high risk features, such as significant ST-depression, anterior ECG changes, diabetes on treatment and if the initial Troponin rise if > than 3 x ULN for that hospital's criteria.

Kite TA, *et al. Heart* 2024; 110:500–507. doi: 10.1136/heartjnl-2023-323513

7.6 RECRUITMENT

Potentially eligible patients will be screened for enrolment as soon as possible at hospital admission, with all recruiting sites undertaking a screening process for all patients with chest pain admitted via A&E or Medical Admissions Units (MAU) or CDU. We would anticipate that the senior clinical nurse on the Unit would inform the team as soon as suitable patients arrive. A pageable research nurse/enrolling physician will review all patients with presenting symptoms as they present, assess any ECG changes, and order immediate Hs-Troponin, calculate the GRACE score once the result is available and if all inclusion criteria are met and there are no exclusion criteria, then the authorised person will gain ASSENT (English and Welsh sites) OR WRITTEN CONSENT, depending on local regulatory guidelines (Sites in Northern Ireland/Scotland may be required to obtain full written consent prior to randomisation). Eligibility can be confirmed by clinical staff who have appropriate training to assess recent onset cardiovascular symptoms indicative of acute coronary syndrome (this may be a physician or a specialist cardiac nurse/Advanced Nurse Practitioner). Posters will be displayed in all MAU, CCUs and cath labs, and we will arrange training days for the study. ASSENT will be in the form of a standard short information sheet that will be read out to the patient. The ASSENT process will be formally documented with date and time and signature of the person obtaining the assent. The Verbal Assent Information Sheet must be counter-signed by an independent witness, this should be a clinical staff member unrelated to the Rapid-NSTEMI trial. All site staff involved in the assent process will be required to sign the assent log provided by the LCTU. Once this has been obtained, the cath lab staff will be informed and the patient will be randomised.

7.7 RANDOMISATION

Randomisation will be performed by the research nurse/enrolling physician using a validated Interactive Voice Response (IVR) or Interactive Web Response (IWR) System ("Sealed Envelope Ltd") provided through the LCTU. Eligible participants will be randomly assigned in a 1:1 ratio to one of two arms:

GROUP A: Immediate angiography with follow-on revascularisation if indicated

or

GROUP B: Standard angiography with follow on revascularisation if indicated, - within Guideline recommended times.
Standard interim therapy such as LMWH/DAPT will be mandated in this group until angiography.

Randomisation will be stratified by hospital site, with minimisation within each hospital site strata for the Grace score category. Due to the nature of the intervention, blinding of the participants or the study team to the randomisation arm is not possible.

7.8 INTERVENTION

If randomised to immediate angiography (+/- intervention), the patient will be transferred to the cardiac catheterisation lab with no further avoidable delay similar to those on a STEMI pathway. At angiography the interventionist will decide, in accordance with usual criteria whether the patient should receive revascularisation (PCI or CABG) or whether they need no further management due to absence of flow limiting coronary obstruction, or whether the disease should be treated with medication alone. Routine practice including use of FFR to inform the clinician will be expected. All decisions will be documented in the eCRF. If the

Kite TA, et al. Heart 2024; 110:500–507. doi: 10.1136/heartjnl-2023-323513

appropriate intervention is PCI, then all things being equal the procedure will be completed at that sitting.
Planned management of any non-infarct related artery (N-IRA) disease will be allowed and documented as being planned at the time of the first intervention.

If randomised to Group B the patient will be managed on an appropriate acute ward and receive subcutaneous LMWH, DAPT (Aspirin plus Ticagrelor/clopidogrel), and secondary prevention medication as per standard practice, including high dose statins. These patients will be listed for coronary angiography as per current standard care for N-STEMI patients for that hospital.
Planned management of any N-IRA disease will be allowed and documented as being planned at the time of the first intervention.
A screening log of potentially eligible but non-included patients with reasons, will be kept and reviewed regularly by the trial management group, screening log data will also be included in the reports to both the TSC and DSMC.

If the immediate Troponin is negative, patients will be considered to be suffering unstable angina and are NOT eligible for randomisation and will be managed according to the responsible physician standard of care for such patients. A second sample may be taken at 3 hours in line with current European guidelines, and if elevated, the participant may be randomised.

All patients will be approached as soon as possible following randomisation for formal consent to remain in the study: a patient information sheet and a consent form for signature will be given to the participant. Once written consent has been obtained participants allocated to group A will complete a quality of life baseline form (EQ-5D-5L).

A repeat Troponin will be undertaken within 24 hours after PCI.

An additional Troponin will be taken from the standard of care group prior to their planned angiography.

Table 1: Troponin & EQ5D-5L

	ADMISSION	FOLLOWING RANDOMISATION	PRE ANGIO ?PROCEED	Within24 HOURS POST PCI
GROUP A<br>EARLY ANGIO?PROCEED	ASSENT*<br>TROPONIN	CONSENT		TROPONIN<br>EQ-5D-5L<br>SAQ
GROUP B<br>STANDARD OF CARE ANGIO?PROCEED	ASSENT*<br>TROPONIN	CONSENT	TROPONIN	TROPONIN<br>EQ-5D-5L<br>SAQ

*or written consent, depending on local regulatory guidelines (applicable to sites in Northern Ireland/Scotland only).

7.9 LOGISTICS

Recognising that it is critical to ensure collaboration across teams and departments from the start, we will ensure all PIs, cath lab nurse managers, and any staff to whom patients present (e.g. A&E), are invited to attend initial local/regional meetings. The senior applicants have been involved in studies where engagement of all stake-holders has been essential (e.g. STREAM trial paramedics). We expect all eligible patients to be approached in each of the sites. The inclusion criteria are broad and the exclusion criteria limited, and mostly to those patients that would require emergent treatment based on haemodynamic instability. Taking account of patient's willingness to be involved in any study and available resources, we would expect to include >75% of patients seen. This strategy will help to ensure that all eligible patients are considered for the trial. An important measure will be the time between randomisation to immediate angiography with proposed follow-on revascularisation (Group A) and the actual time of intervention. Our focus will be on supporting staff (PIs, research nurses, cath lab teams) and engaging them in the trial ethos. We will include large sites with very active ACS programmes and with the cath lab and the A&E/CDU/MAU on the same site to allow for the early group (A) to suffer little delay. Depending on recruitment we expect that extension of the trial to later in the working day and to beyond the working week to be considered as the trial progresses.

Review of ongoing studies that might compete for the same population or whose results might affect recruitment has been undertaken: A search on clinicaltrials.gov has shown no ongoing studies that would compete for recruitment of N-STEMI ACS patients to RAPID-NSTEMI. The BHF study (Senior RITA) may pose competition for sites and patients but we have recruited 25 confirmed sites participating in RAPID-NSTEMI.
The DANCE pilot study aimed to recruit 200 patients from 4 sites in London to assess the efficacy of transporting N-STEMI ACS patients to regional PCI-capable sites for invasive management. ICON-1 study is recruiting ACS patients over 65yrs only within a single centre (n=300).

GLOBAL-LEADERS is an "all comers" study evaluating use of 1 vs 2 antiplatelet agents following PCI; it is not exclusively recruiting N-STEMI patients. None of these studies address the primary question of timing of intervention in the acute phase of N-STEMI and hence the results of these studies would not affect recruitment into RAPID-NSTEMI. Given the UK-wide PCI rates for N-STEMI ACS (approx. 36,000 in 2014) and our broad inclusion criteria, we consider recruitment to RAPID-NSTEMI will be completed within time. Based on UK-wide data from Myocardial Ischaemia National Audit Project (MINAP) and British Cardiovascular Intervention Society BCIS we feel that the required recruitment rate of 4 patients per month per centre will be the minimal requirement needed and should be achievable.

Estimated Consent Rate: With 25 sites recruiting over a period of 24 months, the minimum consent rate from would be 4 patients per month per centre. We have polled and have data to suggest UK Interventionist support for this study. At a minimum of 4 patients per month, 25 sites over 24 months will recruit 2400 patients.

Kite TA, *et al. Heart* 2024; 110:500–507. doi: 10.1136/heartjnl-2023-323513

TABLE 2: SUMMARY OF BASELINE, RANDOMISATION AND FOLLOW-UP PROCEDURES

Procedure / Visit window	On admission	24 hours post admission	Pre-angiography (STANDARD arm Group B only)	Within 24hrs post angiography	Pre-discharge	Day 7 +/- 3 days	30-days (Telephone) +/- 1 week	6months (Telephone) +/- 2weeks	12months Clinic visit/ telephone +/- 4weeks
Eligibility assessment	●								
Demographics	●								
ECG	●	●	●	●					●
Hs-Troponin[1]	●		●	●					
FBC	●	●	●	●					
U&E	●	●	●	●					
Blood sugar	●								
Cholesterol	●								
Medical History	●								
GRACE 2.0 score	●								
Assent[2]	●								
Randomisation	●								
Informed written Consent[3]					●				
Frailty score		●							
CMR[4]						●			
Biomarkers[5]	●			●					
Echocardiogram					●				●
EQ-5D-5L				●			●	●	●
Seattle angina score				●			●	●	●
Clinical Status: MACE events					●		●	●	●
Concurrent medication recording	●		●		●		●	●	●
Adverse Events (SAEs up to 12 month FU)	●	●		●	●	●	●	●	●

[1]Group A = 2 Troponins: on admission and within 24 hours post angiography
Group B = 3 Troponins: on admission, pre angiography and within 24 hours post angiography (patients will have a Troponin measured prior to their procedure taken from the arterial sheath)
[2] Or written consent, depending on local regulatory guidelines (applicable to sites in Northern Ireland/Scotland only)
[3]Written informed consent to be taken as soon as possible following randomisation for English and Welsh sites. Sites in Northern Ireland/Scotland may be required to take written informed consent prior to randomisation
[4]CMRI (Cardiac Magnetic Resonance Imaging) Limited sites participating
[5]Selected sites participating, samples taken at specified time points
FBC = Full blood count
U&E = Urea, electrolytes, creatinine, eGFR

EQ-5D-5L = EuroQol quality of life assessment

7.10 IN-PATIENT MANAGEMENT GUIDELINES

Further details of study related procedures including guidance for consent, clinical care and completing eCRFs will be provided in the study Manual of Operations which will be issued to all sites and will form the basis of site training. PCI procedures will be carried out according to current national and international guidelines and locally accepted practice. Stent implantation is according to local standards of care but drug eluting stents (excluding bioabsorable devices) will be used in all cases unless there are clear contra-indications. Requirement for multi-vessel revascularisation to the N-IRA, will be at the operator's discretion. Prior to PCI, blood will be drawn for FBC, U&Es, according to routine clinical practice and results made available for trial purposes. All patients (except for those in 1 centre) will receive oral Ticagrelor loading dose 180mg on arrival and after diagnosis of N-STEMI ACS as per local practice, a maintenance dose of 90mg twice daily will be prescribed. The site that uses clopidogrel will load the patient with 600 mg of the drug and thereafter prescribe 75 mg per day. The use of Abciximab/Bivalirudin will be discouraged and used **only** where appropriate but this group of patients should form a minority. Such agent use will be recorded in the eCRF. Specific procedural details will be recorded. The use of oral anti-coagulant as per the patient with Atrial Fibrillation will be noted and OAC type recorded.

At 24 hours post PCI routine blood tests are repeated for Hs-Troponin-T/I, FBC and U&E as per usual clinical practice. A further Troponin will be taken in Group B patients prior to their angiography?proceed i.e. after the interim period to the to the procedure (taken from the procedural sheath).

Repeated Hs-Troponin-T/I should be taken again if there any recurrent symptoms of ischaemia. Prior to discharge a transthoracic echocardiogram will be undertaken for LV ejection fraction and regional wall motion abnormality as is standard practice. If the patient is in the CMR sub-study then Cardiac Magnetic Resonance imaging scan will also be undertaken 7+/-3 days to evaluate LV function and infarct size. Additional CMR outcomes are highlighted in the CMR substudy protocol (appendix V).

Adverse events (efficacy and safety end-points) and secondary end-points are collected pre-discharge (Appendix II). Optimal secondary prevention therapy is mandated (Appendix iV).

All medication that is prescribed should be deemed clinically appropriate for the individual patient by the local investigator. All patients will be discharged and will take dual anti-platelet therapy 12 months according to NICE guidelines for ACS. All patients will remain on aspirin (75mg) for the continuation of the study period according to local practice. Patients who require additional anticoagulation can be treated additionally with an anticoagulant (Coumadin, warfarin, novel oral anti-coagulants NOAC) according to the clinical requirements. The CHADS-2-VASC and HAS-BLED scores should be calculated to decide the necessity and risks of anticoagulation. Current ESC Guidelines should be followed.

Patients should receive baseline secondary-prevention medication, including a long-acting beta-blocker along (or heart rate-limiting calcium antagonist) with an ACE-inhibitor (or angiotensin II receptor antagonist [A2RA] if ACE-I intolerant) at maximum tolerated dose. Patients should receive high dose statins unless there is documented prior hypersensitivity

or unacceptable side effects. Lipid profile will be optimized as fully as possible to reach LDL levels <2mmol/L. Patients with diabetes mellitus should be managed aggressively as per local guidelines with a period of insulin therapy to achieve good glycaemic control. Medications should be up-titrated to maximum tolerated dose within secondary prevention recommendation to meet the treatment targets, with the use of additional anti-hypertensive medications permitted. Additional anti-anginal medications, e.g. non-dihydropyridine calcium antagonists or long-acting nitrates/Nicorandil are permitted but their use is not considered a pre-requisite prior to symptom-driven invasive investigation if adequate baseline secondary prevention medication has been established. Cardiac rehabilitation should be offered to all patients including dietary advice and referral for smoking cessation therapies. Within either group, continuous clinical monitoring should be maintained along with usual clinical care. Thus, the onset of new symptoms consistent with ischaemia in haemodynamically stable patients should wherever possible be confirmed by non-invasive imaging test before considering coronary angiography.

Suggested treatment targets include:

Random total cholesterol ≤4mmol/l, LDL≤2mmol/l
BP ≤130/80 mmHg in all patients
HbA1C ≤ 7% in diabetic patients

7.11 PROCEDURES AT DISCHARGE

At discharge patients will have an ECG, the study case report form will be completed. The EQ-5D-5L and Seattle Angina Questionnaire will be completed within 24-hour post PCI in both groups.

7.12 CARDIAC MAGNETIC RESONANCE (APPENDIX V)

Cardiac magnetic resonance scanning will be performed in accordance with the CMR sub-study criteria at day 7+/- 3 days. It is anticipated that the CMR sub-study will performed in a limited number of the participating sites that have easy access to scanning facilities to avoid recruitment bias. Key outcome measures for the CMR sub-study are infarct size, the number of discrete MI (Type 4a), myocardial salvage, the presence and extent of microvascular obstruction, LV volumes and ejection fraction criteria.

Participants will be paid reasonable travel expenses to the sum of £15 for attending the CMR scan visit.

7.13 BIOMARKERS SUBSTUDY (APPENDIX VI)

A substudy testing selected novel biomarkers will be performed in accordance with the Biomarkers substudy protocol attached in Appendix VI. It is anticipated that the Biomarkers substudy will be performed in selected sites. Key outcome measures for the substudy include the predictive ability of the selected biomarkers for obstructive coronary artery disease and requirement of PCI, and main trial clinical outcome measures.

7.14 FOLLOW-UP

Patients will be informed that they should contact their general practitioner if they develop any problems during the course of the study. In general a second anti-anginal agent (e.g. long-acting nitrate/Nicorandil/calcium channel blocker) should be considered before

non-invasive imaging with or without angiography are considered. Patients with major or persistent symptoms will be referred back to the clinical team for review. In this study it is recommended that symptoms are investigated by non-invasive imaging to confirm the likelihood that they are due to myocardial ischaemia, prior to consideration of angiography (+/-). Revascularisation is allowed with ongoing CCS class III symptoms with a negative ischaemia test, if symptoms persist despite 2 anti-anginal medications at maximum tolerated doses. In the event of a further acute coronary syndrome it is anticipated most will undergo repeat angiography.

Follow up at 30 days +/- 1 week

Patients will be contacted by telephone at 30 days +/- 1week. Information on clinical progress and especially on readmission, further investigations, EQ-5D-5L and Seattle Angina Questionnaire and changes to medication will be documented. A complete list of medication being taken will be recorded.

Follow up at 6 months +/- 2 weeks

Patients will be contacted by telephone at 6 months +/- 2 weeks. Information on clinical progress and especially on readmission, further investigations, EQ-5D-5L and Seattle Angina Questionnaire and changes to medication will be documented. A complete list of medication being taken will be recorded.

Follow up at 12 months +/- 4 weeks

Patients will be reviewed in a research clinic at 12 months +/- 4 weeks, and the following tests and outcomes will be recorded for all patients in the randomised study. ECG, echocardiogram, EQ-5D-5L form, Seattle Angina Questionnaire document clinical status and especially any re-admission, the reasons for re-admissions, further investigations and further procedures, any changes in medication. Where sites are not undertaking an echocardiogram or ECG at 12 months or a patient is unable to attend the 12 month clinic visit, outcome data will be collected by telephone.

Participants will be paid reasonable travel expenses to the sum of £15 for attending the 12 month clinic visit.

7.15 CLINICAL EVENT REPORTING

7.15.1 General Potential Risks and Hazards to patients

Fundamental patient risks are standard as both arms receive standard treatment. There are no trial drugs nor devices being tested. This is a strategy trial. The potential risks to all patients are related to those from the PCI procedure: risk of 0.5% Death/MI/CVA/need for emergency CABG, 1% risk of vascular injury. The risk/benefit associated with delay to standard of care time compared to immediate intervention has never been compared, especially in the era of contemporary agents and indeed is the primary conceptual objective of this trial. Any bleeding or vascular complications will however be immediately identified by clinical teams and appropriate clinical management strategies employed as per all routine N-STEMI post-PCI patients. All events will be reported timely as adverse events (AE) or serious adverse events (SAE) as per routine trial governance. There will be TMC, DSMC and CEC oversight. For the CMR sub-study, patients with advanced impaired renal function will not be approached due to risk of Nephrogenic Systemic Fibrosis.

Early stopping: The DSMC will meet on a regular basis (determined by the Independent chair) and they will review data provided by an independent statistician and decisions will be made as to whether the trial should be discontinued, early for safety concerns. We will positively discourage stopping for futility/interim +ve study result. In the event the trial is stopped early, all patients recruited to that point will continue to be followed for the duration of the follow-up period.

7.15.2 ADVERSE EVENTS

All adverse events will be collected from randomisation until discharge from hospital. Thereafter only adverse events deemed serious or those listed as an expected SAE's or a pre-specified clinical outcome measure (see section 7.1.5) will be collected until the end of the trial.

7.15.3 Definitions of Adverse Events

Defined as any untoward medical occurrence

7.15.4 Definitions of Serious Adverse Events (SAE)

A Serious Adverse Event (SAE) is defined as any untoward medical occurrence that:
• Results in death
• Is life-threatening
NB: The term "life-threatening" in the definition of "serious adverse event" refers to an event in which the patient was at risk of death at the time of the event; it does not refer to an event which hypothetically might have caused death if it were more severe.
• Requires hospitalisation or prolongation of existing inpatient's hospitalisation
• Results in persistent or significant disability or incapacity
• Results in a congenital anomaly or birth defect

7.15.5 Expected serious adverse events/clinical outcomes

Some serious adverse events occurring in this trial will be expected as a consequence of the underlying disease, routine treatments or diagnostic tests or study related investigational procedures. In addition, the pre-specified clinical outcome measures in the study will not be considered SAEs. The eCRF will be designed to capture expected clinical outcomes.

Expected serious adverse events that do not require expedited reporting are:-

- Death
- Myocardial infarction
- Recurrent ischemia
- Stroke
- Heart failure
- Cardiogenic shock
- Cardiac rupture or tamponade
- Ventricular septal defect
- Valve regurgitation
- Vascular trauma related to PCI procedure
- Arterial or venous embolic events
- Minor and major bleeding

- Revascularisation procedure (PCI or CABG)
- Hospital admission for a cardiovascular cause
- Renal impairment due to pre-existing renal disease and/ or contrast load during angiogram
- Haematoma at angiogram/PCI access site
- Arrhythmias (supraventricular or ventricular)
- Contrast induced confusion post angiogram
- Admission or procedure for a pre-existing condition

All of the expected SAEs listed (not requiring expedited reporting) should be reported via the eCRF within one week of knowledge of the event.

7.15.6 Classifying SAEs

All SAEs will be assessed for causality and relationship to trial.

- Related events are those that are considered to have resulted from the administration of any research procedures. These include PCI, CMR, and myocardial perfusion scanning.
- Unrelated events are those that are not listed in this protocol or the participating sites clinical patient information sheets for procedures.

7.16 SAE REPORTING

Sites will be required to report all unexpected SAEs to the Coordinating Centre via email within 24 hours of identification of the event; a corresponding eCRF should be submitted to MACRO EDC at the earliest possibility. The Coordinating Centre will provide a monthly SAE line listing to the Sponsor, and escalate any priority issues to the Sponsor as required. Investigators will be required to identify if the event is related to the trial. A summary of safety will be included in the annual progress report to the Ethics committee.

Expected SAEs (as listed in 7.15.5) do not require reporting directly to the Coordinating Centre, but should be submitted via MACRO EDC at the earliest opportunity, within one week of awareness.

7.17 END OF TRIAL

The trial will end when all patients have completed the observation period (i.e. when the last patient recruited has completed the 12 month follow-up assessment). Longer term “passive” follow up will continue through NHS Digital. Consent for this will be obtained from patients for a period for up to 10 years.

7.18 WITHDRAWAL

Participants may leave the trial by withdrawing their consent to participate at any time for any reason, without compromising the medical care they will receive in relation to the trial. Participants do not have to give a reason for withdrawal but if they provide a reason for leaving the trial this will be documented in the CRF. Any data collected prior to the withdrawal may be used in the final analysis.

8.0 STATISTICAL ASPECTS

8.1 Power calculations

The power calculations are based on the results of several studies including the retrospective TIMACS study (11,22). Subsequently a post-hoc analysis using GRACE score was applied - patients with a GRACE risk score of >140 (equating to the third with the highest risk) in the early-intervention group had a primary outcome 13.9%, and in the delayed group of 21.0%, a reduction of 35% at 6 months. (HR=0.65; 95% CI, 0.48-0.89; P=0.006). We have decided to include admission for heart failure since there is clear evidence that this is an important outcome following admission with N-STEMI ACS (27) with rates between 5.6% (28) and 15% (29). The best data on new heart failure comes from Kaul (30) who reported a 12.8% incidence.
We have also calculated outcomes based on imputing data into the GRACE score 2.0 and on review of all data, concluded that a GRACE 2.0 score of ≥118 or ≥90 with further high risk clinical factors provides similar outcomes to the proposed event rates. Those with the intermediate GRACE 2.0 risk score (i.e. ≥90) are included providing they have one other high risk features not captured by GRACE 2.0 namely anterior “infarction”, ST segment depression, diabetes (on medication) and a Hs Troponin rise > 3x ULN.
Both the GRACE 2.0 of ≥118 or the GRACE 2.0 ≥90 with one of the risk factors, together with the heart failure data, will provide patient with an event rate ~19%.
Since outcomes have improved somewhat over recent times we have powered RAPID-NSTEMI conservatively to detect a 25% reduction in the composite primary end point.

Standard intervention arm (Group B), predicted primary outcome events = 19%.
Immediate Intervention arm (Group A) predicted primary outcome events = 14.25% (odds ratio of 0.71, relative risk reduction of 25%).

Effect size (relative risk reduction)	25%
MACE rate standard PCI group	19%
MACE rate Immediate PCI group	14.25%
Number of patients per group	964
Total number of patients in study	1928
Total number including 5% drop out 5% crossover and 8% CABG treatment.	2314

To achieve a power (1-β) of 80% with a (two sided) α=0.05, 964 patients are required in each arm. Assuming up to 5% drop-outs, 5% crossover, 8% needing CABG 1157 patients will be recruited to each of the treatment arms A and B, leading to a total of 2314 patients. (Risks to complete recruitment **APPENDIX VI**).

Based on the above, we consider the power calculations to be conservatively robust and the proposed sample size of sufficient power to detect a significant (and clinically important) reduction in primary endpoint. The calculations have been performed in STATA v14 using the χ^2 test of equal proportions with the support of the proposed trial statistician.
The trial will follow an adaptive design with closed DSMC review of standard of care event rates.

8.2 STATISTICAL ANALYSIS PLAN

Statistical analyses will be undertaken according to a pre-trial Statistical Analysis Plan (SAP) which will be prepared by the statistician employed on the study. Descriptive characteristics at baseline will be presented by treatment arm.

The Primary endpoint is composite outcome of major cardiovascular events (MACE–Death/MI/admission with heart failure) within 12 months, and secondary analyses will be performed for MACE at 30 days following identical methodology. All analyses will be performed following the intention-to-treat principle, with subsequent “immediate follow-on revascularisation” secondary analyses. The primary analysis will investigate the proportion of participants having a primary outcome in each arm, with OR and 95% confidence interval calculation. Additional analyses will compare events in Group A and B with a “time-to-event” endpoint of the primary outcome occurring up until 12 months. Two-sided 95% confidence intervals will be presented for the primary endpoint and all secondary end-point comparisons between groups. The detailed statistical analysis plan will be produced and agreed with the Trial Steering Committee, Sponsors and trial funders prior to trial initiation. The trial will be registered with clinicaltrials.gov.

A methods paper outlining the trial will be sent to an appropriate recognised journal for publication.

8.3 HEALTH ECONOMIC ANALYSIS

Objective: To determine the cost-effectiveness of immediate PCI compared to standard management of patients presenting with N-STEMI ACS.

Methods: Costs will be estimated from the perspective of the UK National Health Service (NHS). The following data will be extracted from study case report forms (CRFs): the costs associated with the PCI procedure and any staged procedures, procedure time and consumables/equipment e.g. use of GP IIb/IIIa inhibitors and stents), index admission hospital length of stay, including time in coronary care unit/cardiac intensive care. We anticipate the timing of procedures to be an important cost-driver, if more participants in the immediate procedure group received their treatment out of hours. To monitor the cost implications of this, we will conduct a survey to establish on-call scheduling at each site. Cardiac hospital out-patient appointments and hospital readmissions will be monitored up to 12 months follow-up. The in-hospital site of in-patient management will be documented. Unit costs will be assigned to each item of resource use for a standard price year. This will enable the mean cost associated with each treatment option to be estimated.

Clinical (a composite of all-cause mortality, new myocardial infarction and re-admission with heart failure) and quality of life outcome data will be collected. The latter will be based on the EQ-5D-5L (1) and estimated within 24-hours of procedure (alongside formal consent) and at 6 month and 12 month follow-up, enabling QALY (quality-adjusted life year) scores to be derived.

Multiple imputation (dependent on the level of missing data) and regression analysis will be undertaken to estimate the mean difference in cost between the two groups (mean incremental cost) and the mean incremental effect (the mean difference in both the total number of composite events / QALYs). Assuming dominance does not occur (where one option is estimated to be more effective and less costly than the other option), the

incremental cost-effectiveness ratio for both the composite clinical outcome and QALYs will be estimated and assessed in relation to a range of cost-effectiveness thresholds e.g. £20,000-£30,000 per QALY is recommended by NICE (31). The associated level of uncertainty will also be characterised by estimating the probability of each treatment option being cost-effective according to the cost-effectiveness acceptability curve (CEAC) (32). Sensitivity analysis will also be undertaken to assess the robustness of conclusions to changes in key assumptions. All analysis will initially be conducted on an intention-to-treat basis, with sensitivity analysis subsequently comparing trial treatment received per protocol (very early intervention with delayed/standard intervention).

9.0 DATA MANAGEMENT

9.1 DATA ENTRY

Data entry will be conducted by the study delivery team at site. Case report forms (CRFs) will be maintained in electronic form on a validated web based Remote Data Capture (RDC) system called InferMed MACRO provided by the LCTU. Access to the electronic CRF will be for authorised study personnel using their individual access codes. The investigator and designated personnel must ensure accuracy, completeness and timeliness of data reported in the eCRF and all required reports. Data reported on the eCRF that are derived from source documents should be consistent with the source documents or the discrepancies should be explained.

9.2 DATA PROTECTION

Participant's personal data included in study related databases, shall be treated in confidence and in compliance with all applicable laws and regulations. When processing or archiving personal data, the Sponsor or its representative shall take all appropriate measures to safeguard and prevent access to this data by any unauthorised third party. Consent for NHS Number, DOB and gender sex to be used in order to obtain data linkage for NHS Digital will be specifically requested.

9.3 CONFIDENTIALITY

Each participant will be assigned a unique identification number upon recruitment. The database will be password protected and only researchers collecting data will have access.
All personalised information for participants will be kept confidentially at the recruiting site unless there is specific consent and HRA approval for transfer of this to another site for study related purposes.

Paper copies of case report forms (signed eligibility criteria and randomisation CRFs) and questionnaires will be stored in a locked filing cabinet in the relevant research office. Neither hard copies nor electronic files containing personal information will be removed from the research office or stored in a non-secure manner electronically. The study research team will comply with the Data Protection Policy of the UHL, UoL and local NHS Trusts.

9.4 DATA ACCESS

All source data, study documents, participant notes will be made available for monitoring, audits and inspections by the appropriate regulatory bodies, Ethics Committee, the Sponsor, funders, LCTU and members of the Research Governance Team.

All electronic data will be stored on secure network systems, to which only the relevant study staff have access, which is granted by the IT services or the research team. These systems are backed up daily by the UoL IM&T.

9.5 ARCHIVING

On termination of the trial, the study documents are to be archived until at least 15 years after the last reporting of data in an official manuscript.

Kite TA, *et al. Heart* 2024; 110:500–507. doi: 10.1136/heartjnl-2023-323513

10.0 TRIAL ORGANISATION, REGULATION AND OVERSIGHT

10.1 FUNDING SOURCES

The study has been funded by grants from the British Heart Foundation (BHF Clinical Study CS/17/1/32445). Additional support and resources for the trial will be provided by the participating Trusts and their corresponding Comprehensive Local Research Networks (CLRN).

10.2 ETHICAL CONSIDERATIONS

The trial will be conducted according to ICH GCP, relevant regulations and the SOPs and quality management procedures of Sponsor, host organisations and LCTU. Ensuring adherence to the principles of the Medical Research Council Good Clinical Practice guidelines, relevant Data Protection legislation, UK Policy Framework for Health and Social Care Research, Health Research Authority (HRA) regulations and relevant local and national laws. Ethical aspects will be conducted in accordance with the Declaration of Helsinki (Revised 48th General Assembly, Somerset West, Republic of South Africa, October 1996, http://www.wma.net/).

10.3 SPONSOR

The Sponsor is the University Hospitals of Leicester NHS Trust (UHL). The trial sponsor provides ultimate approval of all new versions of the protocol before they become live. UHL will be undertaking the monitoring of the trial, including site monitoring visits and source data verification.

10.4 HEALTH RESEARCH AUTHORITY (HRA) APPROVAL

This protocol and associated documents e.g. assent forms, patient information sheets, consent forms, study posters and any amendments to these will be submitted to the Health Research Authority (HRA) and Research Ethics Committee (REC) for ethical approval.

10.5 TRIAL REGISTRATION

The RAPID-NSTEMI trial will be registered on a recognised clinical trials database (clinicaltrials.gov) prior to recruitment commencing.

10.6 INSURANCE AND INDEMNITY

If the patient is harmed by taking part in this research project there are no specific indemnity and /or compensation arrangements. If a patient is harmed due to negligence, then the patient may have grounds for legal action, but they may have to pay for this. Regardless of this, if they wish to complain about any aspects of the way they have been treated or approached during the research project, the standard National Health Service complaint system will be available to them.

10.7 TRIAL MANAGEMENT GROUP (TMG)

Consisting of the Chief Investigator, Co-applicants, representatives of CTU and additional members as appropriate. The TMG will hold monthly meetings to ensure that the study runs smoothly and according to the pre-agreed timetable.

10.8 TRIAL STEERING COMMITTEE (TSC)

Consisting of at least three independent members, Chief Investigator, a representative of LCTU, representative of the Sponsor, representative of the funder and lay members will be convened. The TSC will be responsible for the scientific and ethical conduct of the study and will supervise progress of the trial. The independent Chair of the TSC will be Dr Nicholas Boon. The trial protocol and subsequent amendments will be approved by the TSC. The TSC members will be required to attend TSC meetings which will be held prior to the start of the trial and as required throughout the trial.

10.9 DATA SAFETY MONITORING COMMITTEE (DSMC)

An independent DSMC will be convened under the auspices of Prof Bernard Gersh and its own independent statistician and members appointed by Prof Gersh. The DSMC will convene annually to provide independent advice on study conduct and safety issues. Meetings will also be held as necessary should any urgent issues occur. The DSMC will develop a charter which describes the framework within which it will operate. It will meet to review data (blinded or un-blinded) provided by the LCTU and advise on whether the trial should be discontinued early for safety concerns.

10.10 CLINICAL EVENTS COMMITTEE (CEC)

A Clinical Events Review Committee (Chair Prof Andreas Baumbach) will be established to determine whether a clinical event indicated by the site team is an appropriate clinical event. It will consist of a total of 3 experienced clinicians. Their reports will be used in the assessment of endpoints.

10.11 TRIAL CO-ORDINATION

Trial coordination will be provided by the Leicester Clinical Trials Unit (LCTU) in collaboration with the Chief Investigator and the Trial Management Group. LCTU is a dedicated, nationally registered trials unit (UKCRC No.43).

LCTU will be responsible for day to day management of the trial including the following: development of the protocol, ethical submissions, development of the specification for the data collection system, data management, meeting arrangements, quality assurance and preparation of trial documentation. LCTU will undertake site initiation visit, provide database training and provide essential documentation in the form of an Investigator Site File (ISF) which will also contain the Manual of Operations. The LCTU will ensure that the trial runs according to the pre-agreed timetable, ethical requirements are complied with, and that all aspects of the study are performed to the highest quality.
The LCTU will provide formal statistical support.

10.12 HEALTH ECONOMICS ANALYSIS

The cost-effectiveness analysis will be undertaken by researchers from the University of East Anglia, based on data collected as part of the study.

10.13 CONSORT

The trial will be reported in line with the CONSORT (Consolidated Standards of Reporting Trials) statement (Lancet 2001, 357: 1191-1194).

10.14 DISSEMINATION & PUBLICATION POLICY

Input from the trial's lay members will inform the dissemination plan for patients and public. Participants will be given a study newsletter when they attend their 12 month clinic visit providing information about the study timelines and when the study results will be known. Access to the findings of the study should be made available in a contemporary and user friendly way and full details of the results provided if the patient requests them.

The Chief Investigator will be responsible for ensuring that the results of the study are disseminated through peer review journals, conference presentations and local mechanisms at all participating centres irrespective of the outcome within 6 months after the database is locked. Authorship on the manuscript will be determined by the Chief Investigator according to contribution to the study after discussion with the Trial Steering Committee and according to the guidelines of leading medical journals. A full list of investigators shall be included in the original publication as an appendix. The TSC will be responsible for approval of all manuscripts arising from the study prior to submission for publication. All publications will quote the clinical trials registration number and will acknowledge the participating investigators, TSC and DSMC, LCTU, the Sponsor and the Funder.

No publication can be considered without the approval of the TSC.

10.15 COMPETING INTERESTS

None of the authors declare any competing interests.

11.0 TRIAL TIMETABLE

Anticipated project timeline:
Start up: August 2017 to May 2018: Protocol preparation, ethics submission documents prepared, study approvals, centre identification, centre invitations
Finalise protocol: May 2018 HRA & Ethical Committee application database set up, practical procedures, trial master files:
Site initiation visits and site training: August 2018- January 2019
First patient enrolment: September 2018.
Register trial on clinicaltrials.gov.org: June 2018
Principal Investigators Meeting at ACI: January 2018
Methods paper to be submitted: October 2019
Last patient enrolment: September 2020
Last follow-up: September 2021
Data Analysis: February 2022
Publication and Dissemination: September 2022

APPENDICES

APPENDIX I: GRACE SCORE

(Grace 2.0: Calculator https://www.outcomes-umassmed.org/grace/acs_risk2/index.html)

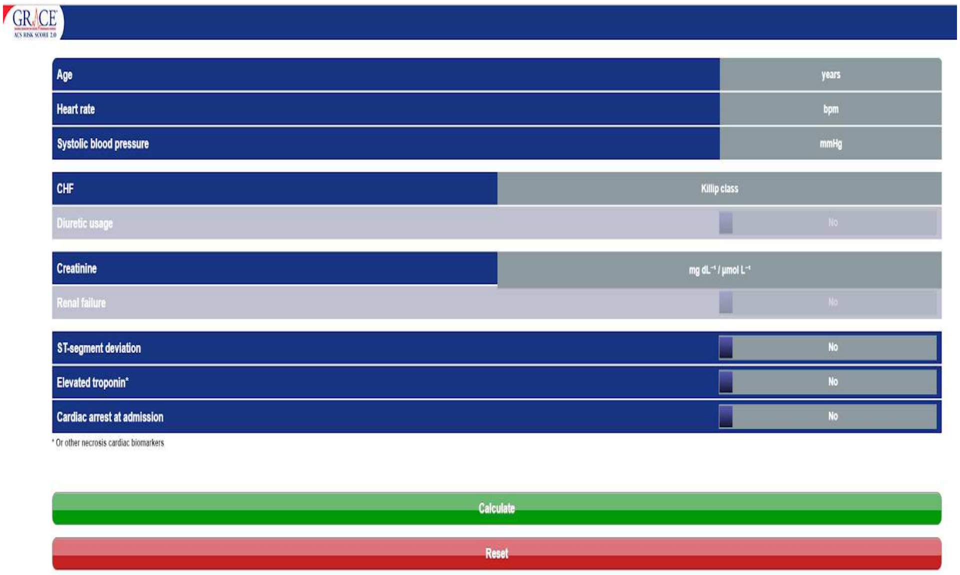

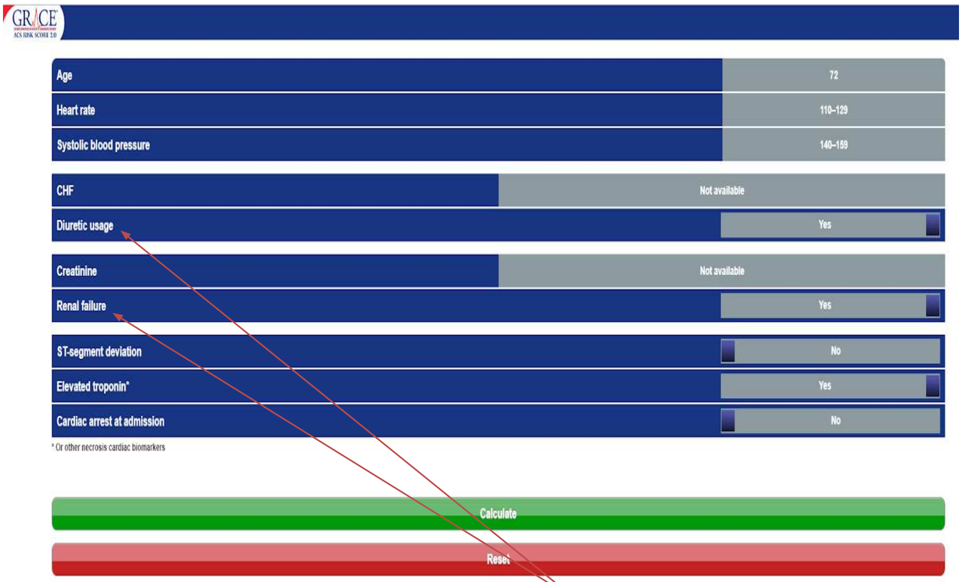

If Killip Class and serum creatinine are not available, please ensure diuretic usage (current or historical) and renal failure fields are completed, as this impacts the final calculation.

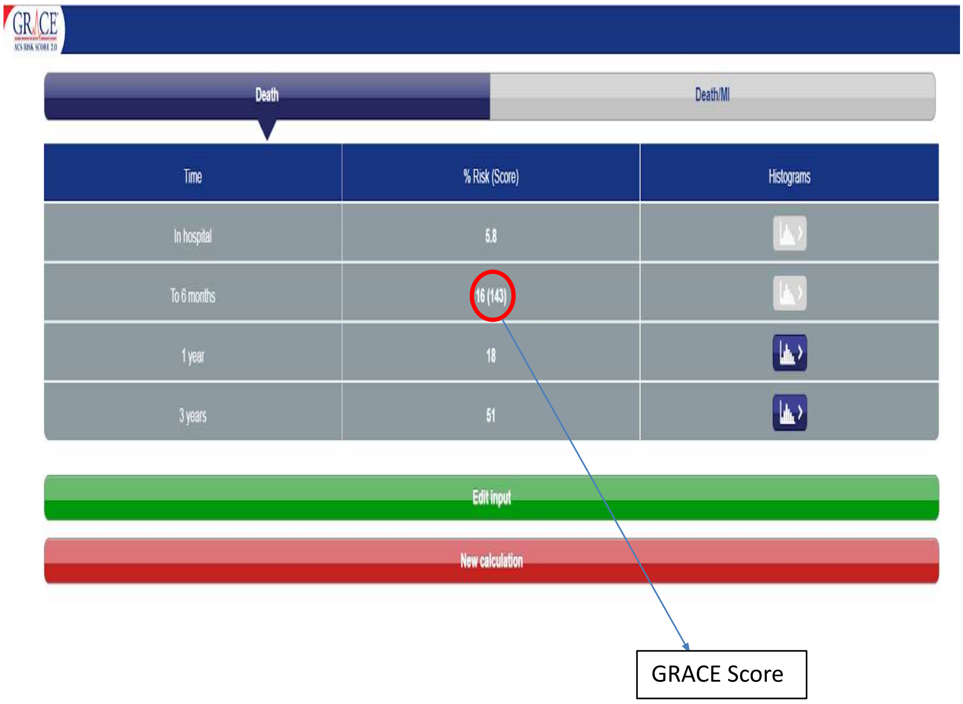
Death
Death/MI
Time
% Risk (Score)
Histograms
In hospital
5.8
To 6 months
16 (143)
1 year
18
3 years
51
Edit input
New calculation
GRACE Score

APPENDIX II: CLINICAL EVENT DEFINITIONS

Full definitions will be provided after review by the Clinical Events Committee (CEC) in the CEC Charter, appropriate Case Report Forms and in the Manual of Operations.

Death

Death from any cause classified as cardiovascular or non-cardiovascular. Cardiovascular death includes any cardiac causes, or other vascular causes (e.g. pulmonary embolism, aortic dissection).

Myocardial infarction (MI):

Myocardial infarction will require a hospital admission with one or more of the following:

Type 1: Spontaneous re-MI: Recurrent angina symptoms or new ECG changes occurring before PCI or <48 hours from PCI that is compatible with re-MI associated with an elevation of CK-MB, Hs-Troponin-I/T, or total CK beyond ULN and 20% or more above the previous value.

Type 4a: CK-MB or total CK >3 times the ULN within 48 hours following PCI. If the pre-PCI CK-MB or total CK level is higher than the ULN, there also needs to be:

Either the demonstration of a falling CK-MB or total CK level prior to the onset of the suspected event, or a subsequent peak of the cardiac biomarker of at least 20% above the previous value obtained prior to the onset of the suspected event.

Heart failure

Heart failure will be defined as a hospital admission with any of the following symptoms and signs: worsening breathlessness, fatigue, fluid overload, pulmonary oedema, elevated venous pressure and elevated BNP. Confirmation of heart failure according to local expert judgement and evidence of impaired LV function will be required for the event to be classified as heart failure.

Repeat Revascularisation

Repeat revascularizations will be symptom/ ischaemic burden driven and classified as:

target lesion re-interventions (TLR) inside the implanted stent or within 5 mm proximally or distally or repeated interventions in the same vessel (TVR) by percutaneous coronary interventions (PCI) or by coronary artery bypass graft surgery.

PCI to lesions not identified previously

CABG for new symptoms or complications of PCI

Stent thrombosis

Stent thrombosis (ST) will be classified as "acute"- within 24 hours from the procedure, "sub-acute" up to 30 days, "late" from 30 days till 1 year and "very late" after 1 year after index procedure. Thrombosis will be classified as definite, probable and possible according to the definition of Academic Research Consortium [25]. ST will be defined as the occurrence of one of the following events:

1. Angiographic documentation of complete or partial stent occlusion and target vessel related acute clinical ischemic event.
2. Autopsy documentation of complete or partial thrombotic stent occlusion
3. Myocardial infarction in the distribution of the stented vessel.

We will separately evaluate the incidence of possible ST by including all unexplained death after 30 days.

Kite TA, *et al. Heart* 2024; 110:500–507. doi: 10.1136/heartjnl-2023-323513

Emergency CABG
This will be defined as CABG occurring within 48 hours of an ischaemic event in a patient who was not previously scheduled to have CABG.

Stroke
Defined as the presence of a new focal neurologic deficit thought to be vascular in origin, with signs or symptoms lasting more than 24 hours. It is strongly recommended (but not required) that an imaging procedure such as CT scan or MRI be performed. Stroke will be further classified as ischaemic, haemorrhagic or type uncertain.

Major bleeding
Major bleeding defined as the cumulative occurrence of intracranial or intraocular bleeding, haemorrhage at the vascular access site requiring intervention, a reduction in haemoglobin levels of at least 5 grams per decilitre, reoperation for bleeding or transfusion of a blood product (at least 2 units), bleeding causing substantial hypotension requiring the use of inotropic agents. All other bleeding events were considered as minor (i.e. epistaxis, blood traces in the stool etc).
Bleeding Academic Research Consortium definitions:
BARC
Type 0: no bleeding
Type 1: bleeding that is not actionable and does not cause the patient to seek unscheduled performance of studies, hospitalization, or treatment by a healthcare professional; may include episodes leading to self-discontinuation of medical therapy by the patient without consulting a healthcare professional
Type 2: any overt, actionable sign of haemorrhage (eg, more bleeding than would be expected for a clinical circumstance, including bleeding found by imaging alone) that does not fit the criteria for type 3, 4, or 5 but does meet at least one of the following criteria: (1) requiring nonsurgical, medical intervention by a healthcare professional, (2) leading to hospitalization or increased level of care, or (3) prompting evaluation
Type 3
Type 3a:
Overt bleeding plus haemoglobin drop of 3 to <5 g/dL* (provided haemoglobin drop is related to bleed)
Any transfusion with overt bleeding
Type 3b:
Overt bleeding plus haemoglobin drop ≥5 g/dL* (provided haemoglobin drop is related to bleed)
Cardiac tamponade
Bleeding requiring surgical intervention for control (excluding dental/nasal/skin/haemorrhoid)
Bleeding requiring intravenous vasoactive agents
Type 3c:
Intracranial haemorrhage (does not include micro-bleeds or haemorrhagic transformation, does include intra-spinal)
Subcategories confirmed by autopsy or imaging or lumbar puncture
Intraocular bleed compromising vision
Type 4: CABG-related bleeding
Perioperative intracranial bleeding within 48 h

Reoperation after closure of sternotomy for the purpose of controlling bleeding
Transfusion of ≥5 U whole blood or packed red blood cells within a 48-h period†
Chest tube output ≥2L within a 24-h period
Type 5: fatal bleeding
Type 5a: Probable fatal bleeding; no autopsy or imaging confirmation but clinically suspicious
Type 5b: Definite fatal bleeding; overt bleeding or autopsy or imaging confirmation

CABG indicates coronary artery bypass graft. Platelet transfusions should be recorded and reported but are not included in these definitions until further information is obtained about the relationship to outcomes. If a CABG-related bleed is not adjudicated as at least a type 3 severity event, it will be classified as not a bleeding event. If a bleeding event occurs with a clear temporal relationship to CABG (i.e. within a 48-h time frame) but does not meet type 4 severity criteria, it will be classified as not a bleeding event.
↵* Corrected for transfusion (1 U packed red blood cells or 1 U whole blood=1 g/dL haemoglobin).
↵† Cell saver products are not counted.

Surgical repair of a vascular complication
In general, this will refer to surgical repair to the femoral or radial arteries following PPCI but could refer to venous complications or in unusual circumstances repairs to the aorta or carotid arteries.
The outcomes will be according to the VARC-2 classification

VARC-2 Definitions Major Vascular Complications
1. Any aortic dissection, rupture, left ventricular perforation, or new apical aneurysm/pseudoaneurysm
2. Access site or access-site related vascular injury (dissection, perforation, stenosis, hematoma, etc.) leading to death, life threatening or major bleeding, visceral ischemia, or neurological impairment
3. Distal embolization requiring vascular surgery, amputation, or irreversible end organ damage
4. Unplanned endovascular or surgical intervention associated with death, major bleeding, visceral ischemia, or neurological impairment
5. Any new ipsilateral lower extremity ischemia
6. Surgery for access site-related nerve injury
7. Permanent access site-related nerve injury

Minor Vascular Complications
1. Access site or access-site related vascular injury (dissection, perforation, stenosis, hematoma, etc.) NOT leading to death, life threatening or major bleeding, visceral ischemia, or neurological impairment
2. Distal embolization treated with embolectomy and/or thrombectomy and NOT resulting in amputation or end organ damage
3. Unplanned endovascular stenting or unplanned surgical intervention not meeting the criteria for a major vascular complication
4. Vascular repair or the need for vascular repair (via surgery, ultrasound-guided compression, transcatheter embolization.

APPENDIX III: Symptoms of ischaemia

Definitions of N-STEMI
*Ischaemic symptoms
From the ESC 2014 Guideline on Management of Acute coronary Syndromes without ST-segment elevation3:
Anginal pain in NSTE-ACS patients may have the following presentations:

† Prolonged (>20 min) anginal pain at rest;

† New onset (de novo) angina (class II or III of the Canadian Cardiovascular Society classification)

† Recent destabilization of previously stable angina with at least Canadian Cardiovascular Society Class III angina characteristics (crescendo angina).

Prolonged and de novo/crescendo angina are observed in 80% and 20% of patients, respectively. Typical chest pain is characterized by a retrosternal sensation of pressure or heaviness ('angina') radiating to the left arm (less frequently to both arms or to the right arm), neck or jaw, which may be intermittent (usually lasting several minutes) or persistent.
Additional symptoms such as sweating, nausea, abdominal pain, dyspnoea and syncope may be present.
Atypical presentations include epigastric pain, indigestion-like symptoms and isolated dyspnoea. Atypical complaints are more often observed in the elderly, in women and in patients with diabetes, chronic renal disease or dementia.

The exacerbation of symptoms by physical exertion and their relief at rest increase the probability of myocardial ischaemia. The relief of symptoms after nitrates administration is not specific for anginal pain as it is reported also in other causes of acute chest pain.
In patients presenting with suspected MI to the emergency department, overall, the diagnostic performance of chest pain characteristics for MI is limited. Older age, male gender, family history of CAD, diabetes, hyperlipidaemia, hypertension, renal insufficiency, previous manifestation of CAD as well as peripheral or carotid artery disease increase the likelihood of NSTE-ACS.

University Hospitals of Leicester NHS Trust

APPENDIX IV: Recommended Secondary Prevention Therapy

For both the Immediate and Standard care groups, the NSTEMI patients will be commenced on secondary prevention therapy as outline in the ESC guidelines for the management of patients presenting with Non-ST-segment elevation Myocardial Infarction.

For the RAPID NSTEMI trial, these are summarized in the table below:

Category of Secondary Prevention	Summary of Guideline-recommended targets
Lipid Lowering Therapy	Initiation of high-intensity as early as possible following admission with NSTEMI-ACS.<br>● Statin therapy that lowers LDL cholesterol level by approx. 50%
ACE-inhibition	Commencement of suitable ACE-inhibitor or Angiotensin-Receptor blocker in patients with LV systolic dysfunction or heart failure, diabetes or hypertension.
Beta-blockers	Beta-blockers are recommended in patients with reduced LV systolic dysfunction (LVEF≤40%)
Mineralocorticoid Receptor antagonist	Aldosterone antagonist therapy (Spironolactone or Eplerenone) is recommended in patients with LV systolic dysfunction (LVEF≤40%) **and** heart failure or diabetes following NSTEMI.
Antihypertensive therapy	Antihypertensive therapy (BP goal <140/90 mmHg) is recommended, including use of antihypertensives as outlined in the ESC guidelines on hypertension management.

Adapted from Roffi et al. 2015 ESC guidelines for the management of acute coronary syndromes in patients presenting without persistent ST-segment elevation. Section 5.9.1: Medical Therapy for Secondary Prevention. European Heart Journal 2016; 37: 267-315.

For RAPID NSTEMI, patients should be commenced on appropriate secondary prevention medication providing there is no contra-indication, with dose up-titration according to tolerability and attaining targets as outlined in the table above.
Examples of secondary prevention medication are as below, specific agents used will be determined by local management protocols for patients with NSTEMI:

Category of Secondary Prevention	Examples of agents that can be used
Lipid Lowering Therapy	Atorvastatin<br>Simvastatin
ACE-inhibition	ACE-inhibitors: Ramipril, Enalapril, Lisinopril<br>ARBs: Candesartan, Losartan.
Beta-blockers	Bisoprolol<br>Metoprolol
Mineralocorticoid Receptor antagonist	Spironolactone<br>Eplerenone
Antihypertensive therapy	ACE-inhibitors: Ramipril, Enalapril, Lisinopril<br>ARBs: Candesartan, Losartan.<br>Calcium-channel antagonists: Amlodipine, Felodipine<br>α-blockers: Doxasozin<br>Beta-blockers: Bisporolol, Atenolol

The ORBITA investigators randomised patients with stable angina and a single Type-A lesion to PCI or continued optimized medical therapy. All patients included within this trial underwent an intensive 6-week period of medical therapy optimization prior to randomization.

The medical therapy protocol for the ORBITA study is as below:

**Risk Factor Modification**	**Dose**
Aspirin	75mg OD
Atorvastatin	≥40mg OD
Clopidogrel (or equivalent antiplatelet therapy)	75mg OD
Perindopril (if known hypertension)<br>(or equivanent ACEi or ARB)	≥4mg OD
**Anti-anginal therapy**<br>At least 2 anti-anginals from the following:	**Dose**
Bisporolol<br>(or equivalent β-blocker)	≥5mg OD
Amlodipine<br>(or equivalent calcium-channel antagonist)	≥5mg OD
Isosorbide Mononitrate SR<br>(or equivalent long-acting oral nitrate)	25mg OD
Nicorandil	10mg BD
Ivabradine	7.5mg BD
Ranolazine	500mg BD

From Al-Lamee et al, Lancet 2018; 391(10115): 31-40. Supplementary material Table A3.

The Risk factor modification group of medical therapy essentially includes those within the ESC guidelines with the exception of statins. The table above also indicates types of anti-anginal agents that can be used in stable angina; while these are not necessarily required as secondary prevention medication in NSTEMI patients their use will be monitored especially at follow-up visits to indicate any ongoing angina.

For each patient the following will be documented with regards to secondary prevention medication:

- Whether the patient is on each of the above 5 class of agents outlined in the secondary prevention for ESC guidelines.
- The name and dose of the agent at discharge, and at each follow-up visit
- Any additional anti-anginal medication, including name of agent and dose on discharge and at follow-up visits.
- Heart rate measurement at discharge and at each of the follow-up visits
- Blood pressure measurement on discharge and at each of the follow-up visits
- Baseline lipid profile, including total and LDL cholesterol levels, and measurement of these at subsequent follow-up visits
- Reasons for discontinuation of therapy will also be documented (side effects, allergy, intolerance)

APPENDIX V: CMR SUBSTUDY

Following STEMI the extent of LV dysfunction is strongly related to prognosis. Echocardiographic and radionuclide studies have shown that left ventricular ejection fraction (EF), end-systolic volume index (ESVI) and infarct size (IS) are key predictors of mortality and major adverse cardiovascular events (33,34). More recently, microvascular obstruction [and IS], as assessed by contrast-enhanced CMR has been shown to provide incremental prognostic information over clinical variables and LVEF (35,36). Owing to pathophysiological differences, extrapolation of STEMI data to N-STEMI may not necessarily be valid.

In N-STEMI ACS, (excluding clinical, angiographic and ECG predictors) there are currently limited published data on predictors of outcome using cardiac imaging. Imaging studies confined to N-STEMI are few in number, and are primarily descriptive, documenting the occurrence of smaller infarct size and area at risk (AAR) than in STEACS, and a lower incidence of MVO and adverse remodelling. Prognostic imaging data are limited to small-scale studies documenting the predictive abilities of echocardiographic assessment of diastolic/systolic function, dyssynchrony and post-infarct mitral regurgitation (37–39), with CMR, the predictive abilities of oedema assessment (n=88, discussed below) and MVO (n=61) (37–41) have been assessed.

A single-centre study involving 100 N-STEMI patients showed that those with detectable oedema were 3.4 times more likely to undergo revascularisation than those without and had a higher hazard of a cardiovascular event or death within 6 months (hazard ratio: 4.47, 95% CI=1.00 to 20.03; p=0.05). Oedema imaging may also provide important pathophysiological insight into the mechanisms of injury and recovery in the AAR. Whereas native T1 mapping reflects cellular and extracellular oedema, post-contrast T1 mapping enables assessment of the extracellular compartment in isolation. Interestingly, expansion of the extracellular compartment has been observed not just in the territory of the culprit artery but also remote, apparently ‘normal’ territories (42). This may reflect altered mechanical and electrical function in these territories, and extracellular volume has been shown strongly to predict clinical outcome (43).

Thus, the application of multi-parametric CMR in patients with N-STEMI, incorporating combined volumetric, IS, MVO and oedema assessment will provide a wealth of mechanistic and prognostic data, with the potential to improve risk prediction in NSTE-ACS.

Rationale for including CMR in RAPIDNSTEMI

This trial focuses on high risk patients with N-STEMI who have ongoing symptoms and elevated Hs-Tn. These patients are therefore behaving similarly to STEMI presentations but without ECG ST elevation which may reflect a smaller area at risk or potentially plaque rupture in the circumflex territory which frequently does not cause ST elevation on the standard chest leads. The assumption is that patients included in the trial will have a very high probability of acute plaque rupture with reduced myocardial blood flow and ongoing myocardial necrosis unless reperfusion is achieved.

Hypotheses

Primary Hypothesis

Immediate angiography with follow-on revascularisation will reduce average myocardial infarction size compared to standard care.

Secondary hypotheses

Immediate angiography with follow-on revascularisation will result in:

Increased myocardial salvage (area at risk-infarct size x 100/area at risk)

Less microvascular obstruction

Higher ejection fraction and lower LV volumes

Infarct size and myocardial salvage index will be linked to prognosis

Residual ischaemia will be linked to prognosis

CMR in these patients will confirm:

- The proportion who have detectable myocardial oedema, which essentially confirms plaque rupture and the territory affected.
- The proportion who have evidence of infarction on Late Gandolinium Enhancement (LGE) and how extensive this is
- The presence and extent of microvascular obstruction, which is generally associated with larger infarcts
- LV ejection fraction and volumes
- Myocardial salvage in those with detectable oedema.
- The frequency and extent of residual ischaemia following revascularisation, which may not be complete

CMR outcomes

Primary: Infarct size on LGE

Secondary: LV ejection fraction; LV end diastolic volume; LV end-systolic volume; LV mass index; Presence of myocardial oedema; Myocardial salvage index (in those with oedema); Presence and extent of microvascular obstruction; presence and extent of ischaemia; native T1 (peak and infarct core) and infarct and remote extracellular volume; global longitudinal and circumferential strain;

CMR procedures

Only sites (6-8) that have ready access to CMR for research purposes will be invited to participate in the sub-study to minimise bias in those recruited. Sites must have experience in the assessment of acute patients and be able to provide test data of high quality. All patients recruited at these sites will be asked to participate in the sub-study which is embedded within the main trial protocol. The protocol will include area at risk assessment, cine imaging in long axes and short axis to calculate LV volumes mass and function. Contrast will be administered to allow detection of myocardial necrosis and microvascular obstruction and where available T1 mapping will be undertaken which gives a measure of area at risk, microvascular obstruction and allows calculation of myocardial extracellular volume that may indicate more subtle changes in oedema and fibrosis.

As LGE has a tendency to overestimate infarct size very early after STEMI and decreases with time following presentation (44,45), it is essential that the timing of CMR is standardized in both arms of the study. In addition as PCI itself may be associated with further myocardial

injury (46), CMR should be performed after angiography+/- PCI in both groups. For these reasons it is intended that the CMR will be performed at day 7 +/- 3 days after presentation. A standard operating procedure for the CMR including additional exclusion criteria, acquisition sequences, and methods for data transfer will be produced. Sub-studies may be undertaken at individual sites. CMR analysis will be undertaken at the University of Leicester core lab, blind to all clinical details as previously described (46).

Power calculation.
This is an exploratory sub-study and formal power calculations have not been performed. As can be seen from the table in the Appendix, the data to date have highly variable inclusion criteria which markedly varying results that makes accurate estimation of infarct size in the patients to be included in RAPID virtually impossible.
The BHF have agreed to fund 200 patients for inclusion to this important mechanistic sub-study**.**

Image analysis
All CMR images will be sent to the core laboratory at the National Institute for Health Research Biomedical Research Centre in Leicester for quality control and central analysis, with the interpreting clinicians blinded to patient information and allocated group.

Analysis will be qualitative and quantitative to include:
1. Image quality (assessed on a grade of 0 to 4 – unanalysable to excellent)
2. Presence of late gadolinium indicating infarction and location based on the AHA/ACC 17 segment model. This will be graded semi-quantitatively as previously described(2, 3) and quantified using the full-width half technique(4)
3. Presence and location of regional wall motion abnormalities and overall regional wall motion score(5)
4. Oedema when present will be quantified (for area at risk) on contrast enhanced cine short axis images(6-8) T1 mapping, thereby allowing quantification of myocardial salvage index
5. Microvascular obstruction from LGE and intramyocardial haemorrhage from quantification of T1 mapping(9)
6. Perfusion analysis at stress and rest scored based on the AHA/ACC 16 segment model(2, 3)
7. Quantitative analysis including end-diastolic volume, end-systolic volume, and ejection fraction

Quantification of global and regional longitudinal and circumferential strain using cine based tracking(10)

Author	Year	Journal	No.	Inclusion	CMR timing	IS	AAR	MVO	Comments
Lemkes	2018	EHJ	142 (124 CMR)	Transient STEMI (acute STEMI with resolution of ECG changes and symptoms)	4.3	1.3% vs 1.5%	N/A	2% vs 3%	Timing of PCI does not have significant effect on infarct size
Layland	2017	JAHA	73	NSTEMI + 1 RF	6.5±3.5d	5.9±8.0%	16%	10 pts	T1m, T2m better than STIR
Loufti	2016	Clin Med Ins Card	30	NSTEMI, stable	5.3±1.0d	10.9 ± 8.5%	N/A	N/A	Echo GLS vs IS
Layland	2015	Circ Im	106	Early invasive Mx	6.1±3.1d 21 before angio	5.4±7.1%	N/A	N/A	Perfusion im vs FFR
Guerra	2014	Int J CVI	190	NSTEMI + PCI in 24h	4.1d	2.1%	16-24%	14%	More MVO in LCx and larger IS
Jensen	2015	Int J Med Sci	69	CP+Tn+TWI/STD	3.8±2.7	10.0±8.5%	N/A	26%	QTd-R_{rel} detects small MI
Thiele	2014	JACC	440	>20min pain, within 72h, Tn↑, culprit with thrombus	1d [1-3]	7.4-8.6%	20-21%	30-32%, 1.4-1.9% of LV	Aspiraton not reduce MVO
Newby L	2014	Lancet	535 [68 CMR]	>45yo, within 24h, Tn↑	3-5d	5.9-8.3%	NAD	NAD	RCT p38 MAPK inhibn – no diff in CMR EPs
Zahid	2014	EHJ CVI	150 [61 CMR]	NSTEACS ref for cor angio	9 ± 3 months	5.4% [1.7-11.4]	NAD	NAD	Echo early systolic lengthening may identify MI
Altiok*	2014	JASE	93 (38 NSTEMI)	Pts treated with PCI					

Buckert	2013	Int J CVI	64	Angio within 72h	59 ± 23 h	NAD	27.6 ± 12.7 %	NAD	CMR vs APPROACH r=0.65
Dall'Armellina	2012	JCMR	41 (9 NSTEMI)	First acute MI	12-48h + 6m	25±12%	34±10%	11%	T1m vs T2w CMR
Xu*	2013	IJCVI	168 (55 NSTEMI)	Early invasive Ix	6d [4-13]	10.7% [5.6-18.1]	20.6 [15.2–29.5]	0%	Lower IS, AAR with NSTEMI
Raman	2010	JACC	100	Age>30, CP+Tn	N/A	N/A	65% of pts	N/A	Oedema – higher risk of MACE at 6m
Grenne	2010	Heart	110	CP>10min, <3d, ref for angio	8±4 months	3-13%	NAD	NAD	Occlusion associated with greater IS
Plein	2008	JCMR	25	CP+ECG+Tn	2–6d	4%	NAD	NAD	Ischaemia in 20%
Cochet	2010	Invest Radiol	61	1st NSTEMI	1 week			28%	MVO predicts MACE 1y

APPENDIX VI: BIOMARKERS SUBSTUDY

Biomarkers substudy

Date and version number: 18.06.2019| Version 1.0

Authors: Dr Tom Kite
Dr Pankaj Gupta
Professor Anthony Gershlick

Biomarkers Substudy Principal Investigator: Dr Pankaj Gupta
Consultant Metabolic Medicine and Chemical Pathology, University Hospitals of Leicester NHS Trust and Honorary Senior Lecturer, Department of Cardiovascular Sciences, University of Leicester
Telephone: 0116 258 6558
Email: pankaj.gupta@uhl-tr.nhs.uk

Co-Investigators

Dr Tom Kite
BHF Clinical Research Fellow
Department of Cardiovascular Sciences
Glenfield Hospital
Groby Road
Leicester
LE3 9QP
Email: tom.kite@leicester.ac.uk

Professor Leon Ng
Professor of Medicine and Therapeutics, Honorary Consultant Physician
Department of Cardiovascular Sciences, University of Leicester
Email: lln1@leicester.ac.uk

Professor Mike Marber

Professor of Cardiology, Honorary Consultant
Cardiologist
King's College London
Guy's & St Thomas' Hospitals
The Rayne Institute, St Thomas' Hospital
Lambeth Palace Road, London, SE1 7EH
Email: mike.marber@kcl.ac.uk

Professor Nick Curzen
Professor of Interventional Cardiology
Wessex Cardiothoracic Unit
Southampton University Hospitals NHS Trust
Southampton, SO16 6YD
Email: nick.curzen@uhs.nhs.uk

NHS University Hospitals of Leicester NHS Trust

1. Introduction

Cardiac troponin (cTn) is now well established as a biomarker of myocardial injury and therefore a critical component in the diagnosis of acute coronary syndrome (ACS), as outlined in the Fourth Universal Definition of Myocardial Infarction (1). Although contemporary high-sensitivity troponins have demonstrated increasing sensitivity for myocardial injury (2), they are widely recognised to be organ-specific rather than disease-specific (3).
Hence, in the acute setting clinicians are repeatedly required to distinguish whether ischaemic or non-ischaemic injury has occurred from the clinical history alongside electrocardiogram (ECG) interpretation. Since ECG changes are found in only 40% of patients diagnosed with non-ST elevation myocardial infarction (NSTEMI) or unstable angina (4), major inconsistencies in the diagnosis of acute myocardial infarction remain a significant problem (5). Furthermore, a recent observational study that measured high-sensitivity troponin (hs-Tn) in 20,000 consecutive patients (in-hospital or outpatient) has raised important questions about the use of the manufacturer-supplied 99th centile as an upper limit of normal threshold with which to diagnose myocardial infarction, as the 99th centile was found to be 6 times the manufacturer derived value (6). Therefore, biomarkers sensitive and specific to degrees of myocardial injury with a defined upper limit of normal appropriate to a hospital population would be a valuable contribution to the clinical arena.

Although elevated cTn levels indicate injury to myocardial cells, they do not reflect the underlying pathophysiological mechanisms and can arise due to a variety of non-ischaemic cardiac and non-cardiac disease processes (7). Improvements in assay sensitivity have lowered diagnostic thresholds but at the expense of reduced specificity, thus leading to diagnostic uncertainty, particularly in the context of uncertainty with regard to assay-specific thresholds for abnormality.
There is therefore a need for novel cardiac biomarkers with superior specificity to provide greater diagnostic capability in patients presenting with recent onset cardiovascular symptoms. These biomarkers may also have a significant role to play in risk stratification and long term prognosis of patients diagnosed with ACS (8).
Desired characteristics of an ideal cardiac biomarker are listed in Table 1.

Table 1

**Characteristics of an ideal cardiac biomarker**
1. High sensitivity
2. High specificity
3. High myocardial concentration and absence in non-myocardial tissues
4. Rapid release into blood and bodily fluids following myocardial injury
5. Rapid clearance from blood and bodily fluids
6. Detection by simple, rapid, and cost-effective assays

7. Well validated threshold differentiating between normal (healthy) and abnormal (disease state positive)

The RapidNSTEMI main study is a prospective randomised controlled trial designed to test the hypothesis that very early angiography with, where appropriate, revascularisation in higher-risk NSTEMI patients will improve clinical outcomes compared with standard care.

The Biomarkers substudy is designed to assess the levels of novel biomarkers in this higher risk NSTEMI population, thereby allowing direct comparison with measured contemporary hs-Tn. Subsequent correlation of these biomarkers with clinical outcomes, and investigation into their potential as predictors of coronary artery disease requiring PCI, will also be assessed to determine whether they are candidates to improve future clinical stratification and triage. Additionally, the unique aspect of participant randomisation to differing treatment strategies will allow direct evaluation of novel biomarker release according to timing of PCI and the impact of procedural related injury on outcomes. Such analyses may allow the identification of these new biomarkers as plausible candidates to refine care pathways in the future.

2. Biomarkers

The RapidNSTEMI Biomarkers substudy will investigate three novel candidate biomarkers that have potential to improve the sensitive and specific detection of ACS, and aid in its risk stratification.

1. **Cardiac myosin-binding protein C (cMyC)** is a cardiac-specific protein showing early promise as a novel biomarker of myocardial injury. Released more rapidly than hs-Tn T or I, cMyC has been shown to provide similar diagnostic accuracy for acute myocardial infarction. However, it may be superior in patients presenting early after symptom onset (9). Use of cMyC as a predictor of long-term survival has suggested comparability to hs-Tn T, and superiority to hs-Tn I (9).

2. **Growth differentiation factor-15 (GDF-15)** is a member of the transforming growth factor beta superfamily (TGF-β) that have been shown to become upregulated in response to oxidative stress, inflammation and tissue injury; all of which occur in myocardial infarction. (10). Previous studies have demonstrated a good correlation with the level of Global Registry of Acute Coronary Events (GRACE) score (11), and superior prediction of major bleeding and cardiovascular events, even when a single measurement of GDF-15 is taken (12).

3. **Suppression of tumorigenicity-2 (ST-2)** is a member of the interleukin-1 (IL-1) receptor family upregulated in myocardial strain, and is suggested to be part of the acute inflammatory response to myocardial injury and infarction (13). Data have demonstrated that levels are increased in NSTEMI and associated with a greater than two-fold increased risk of mortality at 12 months (OR 2.3 [95% CI 1.1-4.6], P =0.03) (14).

3. Aims of substudy

The RapidNSTEMI Biomarkers substudy aims to explore the association between hs-Tn, the tested novel biomarkers, and clinical outcomes in higher-risk NSTEMI patients recruited to the main RapidNSTEMI trial.

Primary aim:

- To investigate in patients with a clinical diagnosis of NSTEMI, the sensitivity and specificity of novel biomarkers (cMyC, GDF-15 and ST-2) in predicting which patients do or do not require PCI following diagnostic angiography.

Secondary aims:

- Correlate levels of each biomarker with cTn taken in the same patient
- Comparative predictive value of each biomarker for primary and secondary main trial outcome measures, and to include in-hospital, 30-day and 6-month rates of major adverse cardiovascular events
- Comparison between biomarkers for predictive values
- Impact of PCI on each biomarker release and subsequent future clinical outcomes
- Positive and negative prediction for type of myocardial infarction (Type 1, Type 2, Type 4a)
- Development of a multivariate model combining candidate biomarkers with risk stratification scoring criteria (e.g. GRACE score) to produce an optimal predictor of clinical outcome

4. Statistics and power calculations

Sample size

With the available resources we will be able to collect and analyse two samples (pre and post-angiography) from 550 patients. It is expected that approximately two-thirds of patients in our cohort will require PCI. If we recruit 550 patients, we expect approximately 367 to require PCI and 183 to not. Based on these 550 patients, 80% power and a 5% significance level, we can calculate the following for the three novel biomarkers.

cMyC

Based on data by Kaier et al (9), and estimating the standard deviation using Wan et al (15) to be 430, we will be able to detect a mean difference in cMyC of 132ng/L between the groups. For cMyC, the 99th percentile cut-off point determined previously in stable patients without obstructive coronary artery disease referred for invasive angiography is 87ng/L (16). Patients diagnosed with acute myocardial infarction were found to have median levels of 237ng/L (9).

GDF-15

Based on data by Wollert et al (17), and estimating the standard deviation using Wan et al (15) to be 780, we will be able to detect a mean difference in GDF-15 of 240ng/L between

the groups. For GDF-15, the 99th percentile cut-off point determined previously in a healthy reference population is 1200ng/L (17). Published data has demonstrated median GDF-15 levels in N-STEMI to be 1499ng/L (17).

ST-2

Based on data by Eggers et al (14), and estimating the standard deviation using Wan et al (15) to be 20, we will be able to detect a mean difference in ST-2 of 6U/ml between the groups. For ST-2, the 99th percentile cut-off point determined previously in a healthy reference population is 31U/ml in men and 21U/ml in women (18). Median levels in patients diagnosed with N-STEMI are 28U/ml with 45% of patients noted to have elevated levels at the point of diagnosis when taking into account gender-specific upper reference levels (14). Again, there are no available data in patients with non-obstructive coronary disease.

The above statistical calculations aim to provide power for the selected biomarkers to predict a clinically meaningful difference between the two groups in whom we are interested; those with obstructive coronary artery disease that require PCI following diagnostic angiography, and those that do not. This is a novel study, and consequently there is limited literature supporting the differences we are likely to see between these two groups. Any results are likely to be hypothesis generating. Despite this, we feel these differences are of clinical importance and hence our proposed sample size of 550 patients is sufficient for our aims.

Statistical analysis

Summary statistics including counts and percentages will be provided for all categorical variables and means and standard deviations for normally distributed continuous variables and medians and interquartile ranges for non-normally distributed continuous variables. The differences in the levels of the biomarkers between the two groups (PCI vs no PCI) will be initially analysed using a two-sample t-test. Appropriate transformations will be made to non-normally distributed variables. Subsequently, multiple linear regression will be performed to adjust for potential confounders (including age and sex). Adjusted means, mean differences, 95% confidence intervals (CIs) and p-values will be provided for comparisons across the two groups.

Based on the previous results showing significant differences between the groups for these novel biomarkers, Receiver Operating Characteristic (ROC) analyses will be performed in order to determine the optimal cut-off points for each biomarker for the requirement of PCI. That is, the cut-point which best provides the best balance between sensitivity (proportion of true positives) and specificity (proportion of true negatives) for the level of biomarker as a diagnostic tool. Sensitivities, specificities and area under the curves will be reported for these cut-offs.

In addition to the above analyses, correlations and comparisons will also be reported between these novel biomarkers and troponin. Clinical outcomes comparing those who do and do not require PCI will be analysed univariately and multivariately with logistic regression. All data will be analysed using the R environment for statistical computing (19).

Clinical implications
Diagnostic coronary angiography currently attracts a National Health Service (NHS) tariff of £831 to £2239, dependent on patient co-morbidity and case complexity (20). Close to 250,000 diagnostic coronary angiograms are performed every year in the UK, with approximately 100,000 of these procedures subsequently proceeding to PCI (21). Therefore, identifying patients with non-obstructive coronary disease who do not require angiography through an appropriately sensitive and specific biomarker panel could result in significant healthcare cost savings, and limit unnecessary patient exposure to procedural risks.

5. Study organisation, regulation and oversight

Clinical event reporting, data management and trial co-ordination will be performed as outlined in the RapidNSTEMI main study protocol (v4.0). Patients recruited to the main study at collaborating sites are eligible and will be approached to participate in the Biomarkers substudy if appropriate. Full written informed consent will be obtained.

Blood samples will be collected from 550 patients across selected RapidNSTEMI sites and stored by the local laboratory until the end of the study period. At the end of the study period, all samples will be dispatched to the Department of Cardiovascular Sciences, Glenfield Hospital for analysis.

A cost of £10 per patient will be allocated for local spinning, storage and shipment of samples.

6. RapidNSTEMI Biomarkers substudy flow chart

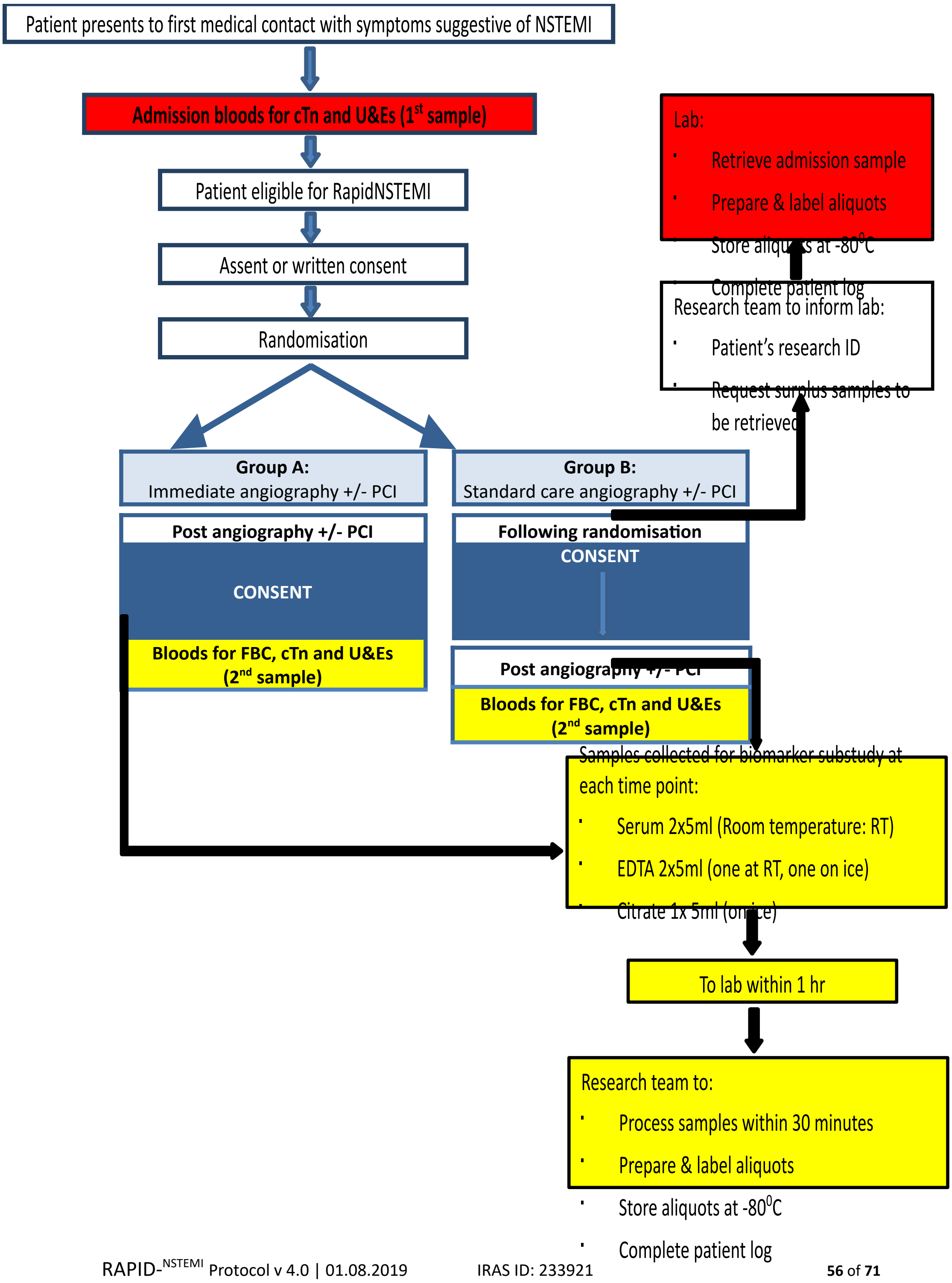

7. Sample collection

The schedule is summarised and highlighted in the flow chart (see Section 6). Members of the clinical or research team with the required expertise will collect samples. When taking blood from the patient, the ante-cubital fossa should be used where possible.

Samples **for all 3 novel biomarkers** will be drawn at the following time points for each group:

- **Group A (Immediate angiography +/- PCI)**
  1. Admission (surplus of sample collected for baseline cTn and U&Es measurement)
  2. Post-angiography +/- PCI

- **Group B (Standard care angiography +/- PCI)**
  1. Admission (surplus of sample collected for baseline cTn and U&Es measurement)
  2. Post-angiography +/- PCI

In selected centres, patients will be approached regarding the substudy as soon as they are randomised into the main trial. Admission samples will be retrieved by laboratory staff, once confirmation has been received from the research team that the patient has consented to participate in the substudy. These will then be collected by a member of the research team for processing and storage (see Section 8).

At time point 2 (post-angiography) the following samples should be collected:

- **Serum:** 2 x 5ml (room temperature)
- **EDTA:** 2 x 5ml (one room temperature, one on ice)
- **Citrate:** 1 x 5ml (on ice)

It is essential that accurate order of draw is followed, and that samples are stored at the correct temperature immediately after collection.

Label the samples with the following information:

- Patient's study ID number
- Date of collection
- Time of collection

If at any point an Adverse Event or Serious Adverse Event is suspected, this must be reported in line with the RapidNSTEMI protocol. If a Needle Stick Injury should occur, guidelines set out in the local Infection Prevention and Control Policy for Sharps Safety must be adhered to and the incident reported to a Line Manager.

- **No patient identifiable information** should be written on any of the sample labels.

- Clearly document in the patients' medical notes what samples were taken, problems encountered and actions taken. Clearly sign, date and time the entry in line with Good Clinical Practice.
- Samples should be **delivered to the local laboratory and separated within 1 hour of collection.**

8. **Sample processing and storage**

All research samples, other than the initial admission samples, are to be processed by the research team.

Admission samples

- Following confirmation of written consent, retrieve admission serum/plasma samples from laboratory
- Carefully remove the supernatant and transfer 2ml aliquots to cryovials
- Label as specified

EDTA and Citrate samples

- Invert the tube 10 times
- Centrifuge for 20 minutes 2500g at room temperature
- Label as specified below
- **For EDTA samples only,** carefully remove the buffy coat (see Section 9: Figure 2) using a pastuer pipette and transfer to cryovial
- Carefully remove the supernatant and transfer 2ml aliquots to cryovials
- Label as specified

Serum samples

- Stand the tubes upright for 30 minutes (for clot formation)
- Centrifuge for 20 minutes 2500g at room temperature
- Carefully remove the supernatant and transfer 2ml aliquots to cryovials
- Label as specified

Sample labelling

- Label each cryovial using indelible ink with the following information:
  - Study name (RapidNSTEMI)
  - Patient's study ID number
  - Sample type and processing (e.g. EDTA RT, EDTA ice, EDTA buffy coat, Citrate ice, Serum, Urine)
  - Date and time of collection
- Complete the sample log (see Section 9: Figure 1) confirming the number of cryovials frozen.
- Please email a copy of the completed sample log once a month to the lab administrator

Storage

- All cryovials need to be stored in storage boxes (see Section 9: Figure 3) at -80°C until the end of the recruitment period.

9. Appendices

Figure 1: Sample Log

Patient Study ID number:
Site ID number:

Time point	Sample type	Date taken	Time taken	Time separated	Time frozen	Volume and number of aliquots		Comments	Lab staff initials
(1,2)	e.g. EDTA ice			Use 24-hour clock		Vol (µl)	Number	e.g. haemolysed, cloudy	

Figure 2: Buffy layer

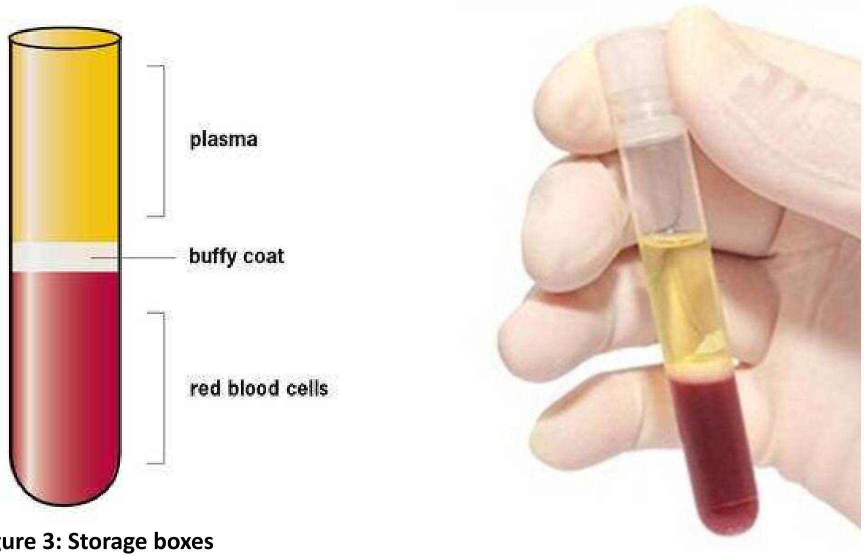

Figure 3: Storage boxes

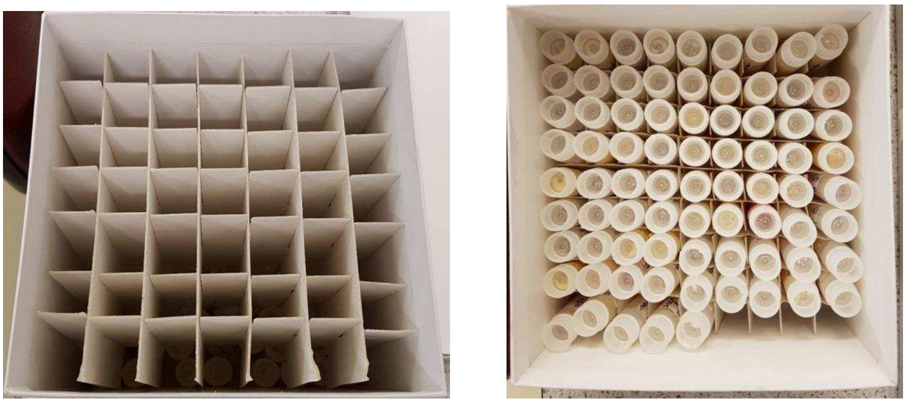

Kite TA, *et al. Heart* 2024; 110:500–507. doi: 10.1136/heartjnl-2023-323513

References

1. Thygesen K, Alpert JS, Jaffe AS, Chaitman BR, Bax JJ, Morrow DA, et al. Fourth universal definition of myocardial infarction (2018). Eur Heart J. 2018.
2. Apple FS, Collinson PO, Biomarkers ITFoCAoC. Analytical characteristics of high-sensitivity cardiac troponin assays. Clin Chem. 2012;58(1):54-61.
3. Agewall S, Giannitsis E, Jernberg T, Katus H. Troponin elevation in coronary vs. non-coronary disease. Eur Heart J. 2011;32(4):404-11.
4. Cannon CP, McCabe CH, Stone PH, Rogers WJ, Schactman M, Thompson BW, et al. The electrocardiogram predicts one-year outcome of patients with unstable angina and non-Q wave myocardial infarction: results of the TIMI III Registry ECG Ancillary Study. Thrombolysis in Myocardial Ischemia. J Am Coll Cardiol. 1997;30(1):133-40.
5. Wu J, Gale CP, Hall M, Dondo TB, Metcalfe E, Oliver G, et al. Editor's Choice - Impact of initial hospital diagnosis on mortality for acute myocardial infarction: A national cohort study. Eur Heart J Acute Cardiovasc Care. 2018;7(2):139-48.
6. Mariathas M AR, Ramamoorthy S, Olechowski B, Hinton J, Azor M, Nicholas Z, Calver A, Corbett S, Mahmoudi M, Rawlins J, Simpson I, Wilkinson J, Cook P, Mamas M, Curzen N. Is the Current Threshold for Diagnosis of “Abnormality”, including Non ST Elevation Myocardial Infarction, using Raised High Sensitivity Troponin Appropriate for a Hospital Population? The CHARIOT Study. British Medical Journal. 2019.
7. Jaffe AS, Wu AH. Troponin release--reversible or irreversible injury? Should we care? Clin Chem. 2012;58(1):148-50.
8. Lindholm D, James SK, Bertilsson M, Becker RC, Cannon CP, Giannitsis E, et al. Biomarkers and Coronary Lesions Predict Outcomes after Revascularization in Non-ST-Elevation Acute Coronary Syndrome. Clin Chem. 2017;63(2):573-84.
9. Kaier TE, Twerenbold R, Puelacher C, Marjot J, Imambaccus N, Boeddinghaus J, et al. Direct Comparison of Cardiac Myosin-Binding Protein C With Cardiac Troponins for the Early Diagnosis of Acute Myocardial Infarction. Circulation. 2017;136(16):1495-508.
10. Kempf T, Eden M, Strelau J, Naguib M, Willenbockel C, Tongers J, et al. The transforming growth factor-beta superfamily member growth-differentiation factor-15 protects the heart from ischemia/reperfusion injury. Circ Res. 2006;98(3):351-60.
11. Widera C, Pencina MJ, Meisner A, Kempf T, Bethmann K, Marquardt I, et al. Adjustment of the GRACE score by growth differentiation factor 15 enables a more accurate appreciation of risk in non-ST-elevation acute coronary syndrome. Eur Heart J. 2012;33(9):1095-104.
12. Hagstrom E, James SK, Bertilsson M, Becker RC, Himmelmann A, Husted S, et al. Growth differentiation factor-15 level predicts major bleeding and cardiovascular events in patients with acute coronary syndromes: results from the PLATO study. Eur Heart J. 2016;37(16):1325-33.
13. Weinberg EO, Shimpo M, De Keulenaer GW, MacGillivray C, Tominaga S, Solomon SD, et al. Expression and regulation of ST2, an interleukin-1 receptor family member, in cardiomyocytes and myocardial infarction. Circulation. 2002;106(23):2961-6.
14. Eggers KM, Armstrong PW, Califf RM, Simoons ML, Venge P, Wallentin L, et al. ST2 and mortality in non-ST-segment elevation acute coronary syndrome. Am Heart J. 2010;159(5):788-94.

15. Wan X, Wang W, Liu J, Tong T. Estimating the sample mean and standard deviation from the sample size, median, range and/or interquartile range. BMC Medical Research Methodology. 2014;14(1):135.
16. Marjot J, Liebetrau C, Goodson RJ, Kaier T, Weber E, Heseltine P, et al. The development and application of a high-sensitivity immunoassay for cardiac myosin-binding protein C. Transl Res. 2016;170:17-25 e5.
17. Wollert KC, Kempf T, Peter T, Olofsson S, James S, Johnston N, et al. Prognostic value of growth-differentiation factor-15 in patients with non-ST-elevation acute coronary syndrome. Circulation. 2007;115(8):962-71.
18. Dieplinger B, Januzzi JL, Jr., Steinmair M, Gabriel C, Poelz W, Haltmayer M, et al. Analytical and clinical evaluation of a novel high-sensitivity assay for measurement of soluble ST2 in human plasma--the Presage ST2 assay. Clin Chim Acta. 2009;409(1-2):33-40.
19. Team RC. R: A language and environment for statistical computing: R Foundation for Statistical Computing, Vienna, Austria; 2017. Available from: https://www.R-project.org/.
20. Imaging BSoC. The challange of national CT coronary angiography (CTCA) provision in response to NICE CG95 update. 2016.
21. Foundation BH. Heart and Circulatory Statistics 2018. 2018.

APPENDIX VII Risks to recruitment

Compliance and loss to follow-up.

The main risk to completing this trial and its robust interpretation will be inappropriately delayed PCI in the very early group and/or early over enthusiastic intervention in the standard arm group with "time contamination". Study discipline is essential.

Other risks include loss to follow-up and patient withdrawal from the study. We have accounted for a potential loss-to-follow-up rate of 5% in our power calculations. The sample also includes those who randomised to standard treatment require urgent intervention (ie crossovers). In addition, some patients may be referred for CABG instead of undergoing PCI if there is evidence of complex lesion anatomy or multi-vessel disease with high SYNTAX score. Again, we estimate from the ABOARD study that this should be ~8% of patients. Furthermore some (a few %) will have normal coronary arteries and others will need to be treated medically (diffuse disease). While all patients randomised to the study will be analysed with "intention to treat" analysis, there will also be a subsequent analysis according to the actual hypothesis being testing: comparing trial treatment received (very early intervention with delayed/standard intervention).

Regarding the recruitment risk to the project, there is always a potential risk of lack of up-take or slow recruitment rate from the participating sites. Thus, we will arrange for 3 regional study-related investigator meetings (Glasgow, Southampton and Oxford) to which 2 members of each centre (PI and research nurse) will be invited to go over trial logistics and recruitment. The clinical research fellow employed for the study will be the important co-ordinator and will work closely with the trial manager to provide regular updates on recruitment rates to participating sites to encourage recruitment. The use of a per-patient recruitment fee should also encourage recruitment to the study by participating sites. This is a portfolio study so will have NIHR funded nurse support.

We will be initiating recruitment in 5 large sites to give the trial a positive kick start.

Kite TA, *et al. Heart* 2024; 110:500–507. doi: 10.1136/heartjnl-2023-323513

University Hospitals of Leicester NHS Trust

APPENDIX VIII Tables of recent protocol changes

SA_02, 01 August 2019

Page	Section	Previous wording	New wording
7-8	2.0 Trial Summary – inclusion and exclusion criteria	Changes as described for sections 7.3.1 and 7.3.2 below	
8	2.0 Trial Summary – Substudies and follow up	Clinical follow-up at 30 days (telephone), 6 months (telephone) and 12 month (clinic visit)	Clinical follow-up at 30 days (telephone), 6 months (telephone) and 12 month (**telephone or** clinic visit)
8	2.0 Trial Summary – Substudies and follow up		Biomarkers sub-study (chosen sites)
10	3.0 Flow Diagram	Assent	Assent***** ***or written consent, depending on local regulatory guidelines (applicable to sites in Northern Ireland/Scotland)**
10	4.0 Scientific abstract	We will undertake one mechanistic CMR sub-study in the two groups	We will undertake a mechanistic CMR sub-study **and biomarkers sub-study in the two groups**
11	5.0 Lay abstract	We will also undertake a special cardiac heart scan called an MRI to see if the heart attack is smaller with earlier balloon and stent.	We will also undertake a special cardiac heart scan called an MRI to see if the heart attack is smaller with earlier balloon and stent, **and measure levels of circulating proteins in the blood called biomarkers**
16	7.1 Aims of the study		**Can novel biomarkers predict need for PCI and future clinical outcomes?**
17	7.2.2 Secondary outcome measures	Additional biomarkers outcomes are specified in the Biomarkers substudy protocol (appendix VI)	Sensitivity and specificity of novel biomarkers in predicting which patients do or do not require PCI following diagnostic angiography**
17	7.3.1 Inclusion Criteria	Elevated high sensitivity Troponin T or I at presentation (above the normal range for individual hospitals)	Elevated high sensitivity Troponin T or I (above the normal range for individual hospitals)
17	7.3.1 Inclusion Criteria	Transfer to the catheter laboratory <u>must</u> be achievable within 4 hours of hospital admission (for participants randomised to Group A)	Randomisation must be performed within 6 hours of hospital admission
17	7.3.1 Inclusion Criteria	Provision of informed assent, followed by formal consent	Provision of assent or written consent
17	7.3.2 Exclusion Criteria	Previous coronary artery bypass grafting	
17	7.3.2 Exclusion Criteria	Planned coronary artery bypass grafting	
17	7.3.2 Exclusion Criteria	Ventricular septal defect or moderate/severe mitral regurgitation	Known severe valvular heart disease
19	7.3 Patient Flow to Randomisation	Assent	Assent***** ***or written consent, depending on local regulatory guidelines (applicable to sites in Northern Ireland/Scotland only)**
20	7.4 Trial Interventions	A GRACE 2.0 score of ≥118, or ≥90 with additional high risk features - see inclusion criteria, will lead to patient and if confirmed then randomisation providing there are no exclusion criteria	A GRACE 2.0 score of ≥118, or ≥90 with additional high risk features - see inclusion criteria, will lead to patient ASSENT **(or written informed consent, depending on local regulatory guidelines. Further details are provided in section 7.6)** and if confirmed then randomisation providing there are no exclusion criteria
20	7.4 Trial interventions		If the initial Hs-Troponin is reported as normal, a second sample may be taken at 3 hours in line with current guidelines. If elevated, the participant may be randomised if all other criteria met.

Kite TA, *et al. Heart* 2024; 110:500–507. doi: 10.1136/heartjnl-2023-323513

Page	Section	Previous wording	New wording
20	7.4 Trial interventions	GROUP A: Immediate angiography with follow-on revascularisation if indicated	GROUP A: Immediate angiography with follow-on revascularisation if indicated - t**ransfer to the catheter laboratory should be performed as soon as possible, and is recommended but not mandated within 90 minutes of randomisation**
20	7.4 Trial Interventions		A biomarkers substudy will investigate if novel biomarkers can predict the need for PCI and future clinical outcomes (APPENDIX VI)
21	7.6 Recruitment	A pageable research nurse/enrolling physician will review all patients with presenting symptoms as they present, assess any ECG changes, and order immediate Hs-Troponin, calculate the GRACE score once the result is available and if all inclusion criteria are met and there are no exclusion criteria, then the authorised person will gain ASSENT.	A pageable research nurse/enrolling physician will review all patients with presenting symptoms as they present, assess any ECG changes, and order immediate Hs-Troponin, calculate the GRACE score once the result is available and if all inclusion criteria are met and there are no exclusion criteria, then the authorised person will gain ASSENT (English and Welsh sites) **OR WRITTEN CONSENT, depending on local regulatory guidelines (Sites in Northern Ireland/Scotland may be required to obtain full written consent prior to randomisation)**.
22	7.8 Intervention		A second sample may be taken at 3 hours in line with current European guidelines, and if elevated, the participant may be randomised
	7.8 Table 1: Troponin & EQ5D-5L	Assent	Assent***** ***or written consent, depending on local regulatory guidelines (applicable to sites in Northern Ireland/Scotland only)**
25	7.9 Logistics Table 2	Following randomisation column removed	
25	7.9 Logistics Table 2		Informed written consent moved to pre-discharge
25	7.9 Logistics Table 2		ECG added at 12 months clinic visit
25	7.9 Logistics Table 2	Removed: Written informed consent to be taken as soon as possible following randomisation	New row with clarification for Assent[2] [2]Or written consent, depending on local regulatory guidelines (applicable to sites in Northern Ireland/Scotland only)
			New row with clarification for Informed Written Consent[3] [3]Written informed consent to be taken as soon as possible following randomisation for English and Welsh sites. Sites in Northern Ireland/Scotland may be required to take written informed consent prior to randomisation
25	7.9 Logistics Table 2		New row with clarification for Biomarkers[5] [5]Selected sites participating, samples taken at specified time points
	7.9 Logistics Table 2		Inclusion of medication recording at Pre-Angiography stage.
	7.9 Logistics Table 2		Removal of medication recording at 24 hours post admission, within 24 hours post angio and Day 7 +/-3
28	7.13 Biomarkers Substudy (Appendix VI)		**A substudy testing selected novel biomarkers will be performed in accordance with the Biomarkers substudy protocol attached in Appendix VI. It is anticipated that the Biomarkers substudy will be performed in selected sites. Key outcome measures for the substudy include the predictive ability of the selected biomarkers for obstructive coronary artery disease and requirement of PCI, and main trial clinical outcome measures.**
29	7.15.5 Expected serious adverse events/clinical outcomes		**Contrast induced confusion post-angiogram**
30	7.15 SAE reporting	Sites will be required to report all SAEs to the Sponsor and the Coordinating Centre within 24 hours of identification of the event. Investigators will be required to identify if the event is related	Sites will be required to report all unexpected SAEs to the Coordinating Centre via email within 24 hours of identification of the event; a corresponding eCRF should be submitted to MACRO EDC at the earliest possibility. The

Kite TA, et al. Heart 2024; 110:500–507. doi: 10.1136/heartjnl-2023-323513

Page	Section	Previous wording	New wording
		to the trial. A summary of safety will be included in the annual progress report to the Ethics committee.	Coordinating Centre will provide a monthly SAE line listing to the Sponsor, and escalate any priority issues to the Sponsor as required. Investigators will be required to identify if the event is related to the trial. A summary of safety will be included in the annual progress report to the Ethics committee.<br><br>Expected SAEs (as listed in section 7.14.5) do not require reporting directly to the Coordinating Centre, but should be submitted via MACRO EDC at the earliest opportunity, within one week of awareness.
39	11 Trial Timetable	Methods paper to be submitted: February 2019	Methods paper to be submitted: October 2019
42	APPENDIX I: GRACE SCORE	Grace 2.0: Calculator www.gracescore.org/website/webversion.aspx	Grace 2.0: Calculator https://www.outcomes-umassmed.org/grace/acs_risk2/index.html
42	APPENDIX I: GRACE SCORE	Previous layout included two screenshots of GRACE 2.0 calculator	Inclusion of a third screenshot, with the text 'If Killip Class and serum creatinine are not available, please ensure diuretic usage (current or historical) and renal failure fields are completed, as this impacts the final calculation.'
52	APPENDIX VI: BIOMARKERS SUBSTUDY		Inclusion of appendix

Kite TA, *et al. Heart* 2024; 110:500–507. doi: 10.1136/heartjnl-2023-323513

REFERENCES

1. Mehta SR, Yusuf S, Peters RJ, Bertrand ME, Lewis BS, Natarajan MK, et al. Effects of pretreatment with clopidogrel and aspirin followed by long-term therapy in patients undergoing percutaneous coronary intervention: the PCI-CURE study. Lancet (London, England) [Internet]. 2001 Aug 18 [cited 2017 Jun 11];358(9281):527–33. Available from: http://www.ncbi.nlm.nih.gov/pubmed/11520521
2. Wiviott SD, Antman EM, Gibson CM, Montalescot G, Riesmeyer J, Weerakkody G, et al. Evaluation of prasugrel compared with clopidogrel in patients with acute coronary syndromes: design and rationale for the TRial to assess Improvement in Therapeutic Outcomes by optimizing platelet InhibitioN with prasugrel Thrombolysis In Myocardial Infarction 38 (TRITON-TIMI 38). Am Heart J [Internet]. 2006 Oct [cited 2017 Jun 11];152(4):627–35. Available from: http://www.ncbi.nlm.nih.gov/pubmed/16996826
3. Wallentin L, Becker RC, Budaj A, Cannon CP, Emanuelsson H, Held C, et al. Ticagrelor versus Clopidogrel in Patients with Acute Coronary Syndromes. N Engl J Med [Internet]. 2009 Sep 10 [cited 2017 Jun 11];361(11):1045–57. Available from: http://www.ncbi.nlm.nih.gov/pubmed/19717846
4. Steinhubl SR, Berger PB, Mann JT, Fry ETA, DeLago A, Wilmer C, et al. Early and sustained dual oral antiplatelet therapy following percutaneous coronary intervention: a randomized controlled trial. JAMA [Internet]. 2002 Nov 20 [cited 2017 Jun 11];288(19):2411–20. Available from: http://www.ncbi.nlm.nih.gov/pubmed/12435254
5. Montalescot G, Bolognese L, Dudek D, Goldstein P, Hamm C, Tanguay J-F, et al. Pretreatment with Prasugrel in Non?ST-Segment Elevation Acute Coronary Syndromes. N Engl J Med [Internet]. Massachusetts Medical Society ; 2013 Sep 12 [cited 2017 Jun 11];369(11):999–1010. Available from: http://www.nejm.org/doi/10.1056/NEJMoa1308075
6. Brener SJ, Murphy SA, Gibson CM, DiBattiste PM, Demopoulos LA, Cannon CP, et al. Efficacy and safety of multivessel percutaneous revascularization and tirofiban therapy in patients with acute coronary syndromes. Am J Cardiol [Internet]. 2002 Sep 15 [cited 2017 Jun 11];90(6):631–3. Available from: http://www.ncbi.nlm.nih.gov/pubmed/12231091
7. Wallentin L, Lagerqvist B, Husted S, Kontny F, Ståhle E, Swahn E. Outcome at 1 year after an invasive compared with a non-invasive strategy in unstable coronary-artery disease: the FRISC II invasive randomised trial. FRISC II Investigators. Fast Revascularisation during Instability in Coronary artery disease. Lancet (London, England) [Internet]. 2000 Jul 1 [cited 2017 Jun 11];356(9223):9–16. Available from: http://www.ncbi.nlm.nih.gov/pubmed/10892758
8. Fox KAA, Poole-Wilson PA, Henderson RA, Clayton TC, Chamberlain DA, Shaw TRD, et al. Interventional versus conservative treatment for patients with unstable angina or non-ST-elevation myocardial infarction: the British Heart Foundation RITA 3 randomised trial. Randomized Intervention Trial of unstable Angina. Lancet (London, England) [Internet]. 2002 Sep 7 [cited 2017 Jun 11];360(9335):743–51. Available from: http://www.ncbi.nlm.nih.gov/pubmed/12241831
9. Biondi-Zoccai GGL, Abbate A, Agostoni P, Testa L, Burzotta F, Lotrionte M, et al. Long-term benefits of an early invasive management in acute coronary syndromes depend on intracoronary stenting and aggressive antiplatelet treatment: A metaregression. Am Heart J [Internet]. 2005 Mar [cited 2017 Jun 11];149(3):504–11. Available from: http://www.ncbi.nlm.nih.gov/pubmed/15864240
10. Boden WE. "Routine invasive" versus "selective invasive" approaches to non-ST-segment elevation acute coronary syndromes management in the post-stent/platelet inhibition era. J Am Coll Cardiol [Internet]. 2003 Feb 19 [cited 2017 Jun 11];41(4 Suppl S):113S–122S. Available from: http://www.ncbi.nlm.nih.gov/pubmed/12644349
11. Mehta SR, Cannon CP, Fox KAA, Wallentin L, Boden WE, Spacek R, et al. Routine vs Selective Invasive Strategies in Patients With Acute Coronary Syndromes. JAMA [Internet]. 2005 Jun 15 [cited 2017 Jun 11];293(23):2908. Available from: http://www.ncbi.nlm.nih.gov/pubmed/15956636
12. Hamm CW, Bassand J-P, Agewall S, Bax J, Boersma E, Bueno H, et al. ESC Guidelines for the management of acute coronary syndromes in patients presenting without persistent ST-segment elevation: The Task Force for the management of acute coronary syndromes (ACS) in patients presenting without persistent ST-segment elevation of the European Society of Cardiology (ESC). Eur Heart J [Internet]. 2011 Dec 1 [cited 2017 Jun 11];32(23):2999–3054. Available from: http://www.ncbi.nlm.nih.gov/pubmed/21873419
13. de Winter RJ, Windhausen F, Cornel JH, Dunselman PHJM, Janus CL, Bendermacher PEF, et al. Early Invasive versus Selectively Invasive Management for Acute Coronary Syndromes. N Engl J Med [Internet]. Massachusetts Medical Society ; 2005 Sep 15 [cited 2017 Jun 11];353(11):1095–104. Available from: http://www.nejm.org/doi/abs/10.1056/NEJMoa044259
14. Hirsch A, Windhausen F, Tijssen JG, Verheugt FW, Cornel JH, de Winter RJ, et al. Long-term outcome

after an early invasive versus selective invasive treatment strategy in patients with non-ST-elevation acute coronary syndrome and elevated cardiac troponin T (the ICTUS trial): a follow-up study. Lancet [Internet]. 2007 Mar 10 [cited 2017 Jun 11];369(9564):827–35. Available from: http://www.ncbi.nlm.nih.gov/pubmed/17350451

15. Montalescot G, Cayla G, Collet J-P, Elhadad S, Beygui F, Le Breton H, et al. Immediate vs Delayed Intervention for Acute Coronary Syndromes. JAMA [Internet]. 2009 Sep 2 [cited 2017 Jun 11];302(9):947. Available from: http://www.ncbi.nlm.nih.gov/pubmed/19724041
16. Riezebos RK, Ronner E, ter Bals E, Slagboom T, Smits PC, ten Berg JM, et al. Immediate versus deferred coronary angioplasty in non-ST-segment elevation acute coronary syndromes. Heart [Internet]. 2009 May 1 [cited 2017 Jun 11];95(10):807–12. Available from: http://www.ncbi.nlm.nih.gov/pubmed/19098058
17. Thiele H, Rach J, Klein N, Pfeiffer D, Hartmann A, Hambrecht R, et al. Optimal timing of invasive angiography in stable non-ST-elevation myocardial infarction: the Leipzig Immediate versus early and late PercutaneouS coronary Intervention triAl in NSTEMI (LIPSIA-NSTEMI Trial). Eur Heart J [Internet]. 2012 Aug [cited 2017 Jun 11];33(16):2035–43. Available from: http://www.ncbi.nlm.nih.gov/pubmed/22108830
18. Milosevic A, Vasiljevic-Pokrajcic Z, Milasinovic D, Marinkovic J, Vukcevic V, Stefanovic B, et al. Immediate Versus Delayed Invasive Intervention for Non-STEMI Patients. JACC Cardiovasc Interv [Internet]. 2016 Mar 28 [cited 2017 Jun 11];9(6):541–9. Available from: http://www.ncbi.nlm.nih.gov/pubmed/26777321
19. Katritsis DG, Siontis GCM, Kastrati A, van't Hof AWJ, Neumann F-J, Siontis KCM, et al. Optimal timing of coronary angiography and potential intervention in non-ST-elevation acute coronary syndromes. Eur Heart J [Internet]. 2011 Jan [cited 2017 Jun 11];32(1):32–40. Available from: http://www.ncbi.nlm.nih.gov/pubmed/20709722
20. Navarese EP, Gurbel PA, Andreotti F, Tantry U, Jeong Y-H, Kozinski M, et al. Optimal Timing of Coronary Invasive Strategy in Non–ST-Segment Elevation Acute Coronary Syndromes. Ann Intern Med [Internet]. 2013 Feb 19 [cited 2017 Jun 11];158(4):261. Available from: http://www.ncbi.nlm.nih.gov/pubmed/23420234
21. Fox KAA, Clayton TC, Damman P, Pocock SJ, de Winter RJ, Tijssen JGP, et al. Long-Term Outcome of a Routine Versus Selective Invasive Strategy in Patients With Non?ST-Segment Elevation Acute Coronary Syndrome. J Am Coll Cardiol [Internet]. 2010 Jun 1 [cited 2017 Jun 11];55(22):2435–45. Available from: http://www.ncbi.nlm.nih.gov/pubmed/20359842
22. Mehta SR, Granger CB, Boden WE, Steg PG, Bassand J-P, Faxon DP, et al. Early versus Delayed Invasive Intervention in Acute Coronary Syndromes. N Engl J Med [Internet]. Massachusetts Medical Society ; 2009 May 21 [cited 2017 Jun 11];360(21):2165–75. Available from: http://www.nejm.org/doi/abs/10.1056/NEJMoa0807986
23. Roffi M, Patrono C, Collet J-P, Mueller C, Valgimigli M, Andreotti F, et al. 2015 ESC Guidelines for the management of acute coronary syndromes in patients presenting without persistent ST-segment elevation. Eur Heart J [Internet]. 2016 Jan 14 [cited 2017 Jun 11];37(3):267–315. Available from: http://www.ncbi.nlm.nih.gov/pubmed/26320110
24. Mueller C, Giannitsis E, M?ckel M, Huber K, Mair J, Plebani M, et al. Rapid rule out of acute myocardial infarction: novel biomarker-based strategies. Eur Hear J Acute Cardiovasc Care [Internet]. SAGE PublicationsSage UK: London, England; 2017 Apr [cited 2017 Jun 11];6(3):218–22. Available from: http://journals.sagepub.com/doi/10.1177/2048872616653229
25. Boeddinghaus J, Reichlin T, Cullen L, Greenslade JH, Parsonage WA, Hammett C, et al. Two-Hour Algorithm for Triage toward Rule-Out and Rule-In of Acute Myocardial Infarction by Use of High-Sensitivity Cardiac Troponin I. Clin Chem [Internet]. 2016 Mar 1 [cited 2017 Jun 11];62(3):494–504. Available from: http://www.ncbi.nlm.nih.gov/pubmed/26797687
26. Reichlin T, Cullen L, Parsonage WA, Greenslade J, Twerenbold R, Moehring B, et al. Two-hour Algorithm for Triage Toward Rule-out and Rule-in of Acute Myocardial Infarction Using High-sensitivity Cardiac Troponin T. Am J Med [Internet]. 2015 Apr [cited 2017 Jun 11];128(4):369–379.e4. Available from: http://www.ncbi.nlm.nih.gov/pubmed/25446294
27. N??ez-Gil IJ, Garc?a-Rubira JC, Luaces M, Vivas D, De Agust?n JA, Gonz?lez-Ferrer JJ, et al. Mild heart failure is a mortality marker after a non-ST-segment acute myocardial infarction. Eur J Intern Med [Internet]. 2010 Oct [cited 2017 Jun 11];21(5):439–43. Available from: http://www.ncbi.nlm.nih.gov/pubmed/20816601
28. Wylie J V, Murphy SA, Morrow DA, de Lemos JA, Antman EM, Cannon CP. Validated risk score predicts

the development of congestive heart failure after presentation with unstable angina or non-ST?elevation myocardial infarction: results from OPUS-TIMI 16 and TACTICS-TIMI 18. Am Heart J [Internet]. 2004 Jul [cited 2017 Jun 11];148(1):173–80. Available from: http://www.ncbi.nlm.nih.gov/pubmed/15215808

29. Franco E, N??ez-Gil IJ, Vivas D, Ruiz Mateos B, Iba?ez B, Gonzalo N, et al. Heart failure and non-ST-segment elevation myocardial infarction: A review for a widespread situation. Eur J Intern Med [Internet]. 2011 Dec [cited 2017 Jun 11];22(6):533–40. Available from: http://linkinghub.elsevier.com/retrieve/pii/S0953620511001543

30. Kaul P, Ezekowitz JA, Armstrong PW, Leung BK, Savu A, Welsh RC, et al. Incidence of heart failure and mortality after acute coronary syndromes. Am Heart J [Internet]. Elsevier; 2013 Mar [cited 2017 Jun 11];165(3):379–85.e2. Available from: http://www.ncbi.nlm.nih.gov/pubmed/23453107

31. NICE. Guide to the methods of technology appraisal 2013 | Guidance and guidelines | NICE. NICE; [cited 2017 Jun 11]; Available from: https://www.nice.org.uk/process/pmg9/chapter/foreword

32. Fenwick E, Claxton K, Sculpher M. Representing uncertainty: the role of cost-effectiveness acceptability curves. Health Econ [Internet]. 2001 Dec [cited 2017 Jun 11];10(8):779–87. Available from: http://www.ncbi.nlm.nih.gov/pubmed/11747057

33. Burns RJ, Gibbons RJ, Yi Q, Roberts RS, Miller TD, Schaer GL, et al. The relationships of left ventricular ejection fraction, end-systolic volume index and infarct size to six-month mortality after hospital discharge following myocardial infarction treated by thrombolysis. J Am Coll Cardiol [Internet]. 2002 [cited 2017 Jun 11];39(1):30–6. Available from: http://www.sciencedirect.com/science/article/pii/S0735109701017119

34. La Rovere MT, Bigger JT, Marcus FI, Mortara A, Schwartz PJ. Baroreflex sensitivity and heart-rate variability in prediction of total cardiac mortality after myocardial infarction. ATRAMI (Autonomic Tone and Reflexes After Myocardial Infarction) Investigators. Lancet (London, England) [Internet]. 1998 Feb 14 [cited 2017 Jun 11];351(9101):478–84. Available from: http://www.ncbi.nlm.nih.gov/pubmed/9482439

35. Regenfus M, Schlundt C, Kr?hner R, Sch?negger C, Adler W, Ludwig J, et al. Six-Year Prognostic Value of Microvascular Obstruction After Reperfused ST-Elevation Myocardial Infarction as?Assessed by Contrast-Enhanced Cardiovascular Magnetic?Resonance. Am J Cardiol [Internet]. 2015 Oct 1 [cited 2017 Jun 11];116(7):1022–7. Available from: http://www.ncbi.nlm.nih.gov/pubmed/26260397

36. Eitel I, de Waha S, Wöhrle J, Fuernau G, Lurz P, Pauschinger M, et al. Comprehensive Prognosis Assessment by CMR Imaging After ST-Segment Elevation Myocardial Infarction. J Am Coll Cardiol [Internet]. 2014 [cited 2017 Jun 11];64(12). Available from: http://www.onlinejacc.org/content/64/12/1217

37. M?ller JE, Egstrup K, K?ber L, Poulsen SH, Nyvad O, Torp-Pedersen C. Prognostic importance of systolic and diastolic function after acute myocardial infarction. Am Heart J [Internet]. 2003 Jan [cited 2017 Jun 11];145(1):147–53. Available from: http://www.ncbi.nlm.nih.gov/pubmed/12514667

38. Ng ACT, Tran DT, Allman C, Vidaic J, Leung DY. Prognostic implications of left ventricular dyssynchrony early after non-ST elevation myocardial infarction without congestive heart failure. Eur Heart J [Internet]. 2010 Feb 1 [cited 2017 Jun 11];31(3):298–308. Available from: http://www.ncbi.nlm.nih.gov/pubmed/19933227

39. Shiran A, Adawi S, Dobrecky-Mery I, Halon DA, Lewis BS. Echocardiographic predictors of late mortality in elderly patients with acute coronary syndromes. Isr Med Assoc J [Internet]. 2007 Apr [cited 2017 Jun 11];9(4):247–51. Available from: http://www.ncbi.nlm.nih.gov/pubmed/17491215

40. Raman S V., Simonetti OP, Winner MW, Dickerson JA, He X, Mazzaferri EL, et al. Cardiac Magnetic Resonance With Edema Imaging Identifies Myocardium at Risk and Predicts Worse Outcome in Patients With Non–ST-Segment Elevation Acute Coronary Syndrome. J Am Coll Cardiol [Internet]. 2010 Jun 1 [cited 2017 Jun 11];55(22):2480–8. Available from: http://www.ncbi.nlm.nih.gov/pubmed/20510215

41. Cochet A, Lalande A, Lorgis L, Zeller M, Beer J-C, Walker PM, et al. Prognostic Value of Microvascular Damage Determined by Cardiac Magnetic Resonance in Non ST-Segment Elevation Myocardial Infarction. Invest Radiol [Internet]. 2010 Nov [cited 2017 Jun 11];45(11):725–32. Available from: http://content.wkhealth.com/linkback/openurl?sid=WKPTLP:landingpage&an=00004424-201011000-00005

42. Sibley CT, Noureldin RA, Gai N, Nacif MS, Liu S, Turkbey EB, et al. T1 Mapping in Cardiomyopathy at Cardiac MR: Comparison with Endomyocardial Biopsy. Radiology [Internet]. 2012 Dec [cited 2017 Jun 11];265(3):724–32. Available from: http://www.ncbi.nlm.nih.gov/pubmed/23091172

43. Wong TC, Piehler K, Meier CG, Testa SM, Klock AM, Aneizi AA, et al. Association Between Extracellular Matrix Expansion Quantified by Cardiovascular Magnetic Resonance and Short-Term Mortality. Circulation [Internet]. 2012 Sep 4 [cited 2017 Jun 11];126(10):1206–16. Available from: http://www.ncbi.nlm.nih.gov/pubmed/22851543

44. Dall'Armellina E, Karia N, Lindsay AC, Karamitsos TD, Ferreira V, Robson MD, et al. Dynamic Changes of Edema and Late Gadolinium Enhancement After Acute Myocardial Infarction and Their Relationship to Functional Recovery and Salvage Index. Circ Cardiovasc Imaging [Internet]. 2011 May 1 [cited 2017 Jun 11];4(3):228–36. Available from: http://www.ncbi.nlm.nih.gov/pubmed/21447711

45. Kidambi A, Mather AN, Swoboda P, Motwani M, Fairbairn TA, Greenwood JP, et al. Relationship between Myocardial Edema and Regional Myocardial Function after Reperfused Acute Myocardial Infarction: An MR Imaging Study. Radiology [Internet]. Radiological Society of North America, Inc.; 2013 Jun [cited 2017 Jun 11];267(3):701–8. Available from: http://pubs.rsna.org/doi/10.1148/radiol.12121516

46. McCann GP, Khan JN, Greenwood JP, Nazir S, Dalby M, Curzen N, et al. Complete Versus Lesion-Only Primary PCI: The Randomized Cardiovascular MR CvLPRIT Substudy. J Am Coll Cardiol [Internet]. 2015 Dec 22 [cited 2016 May 2];66(24):2713–24. Available from: http://www.pubmedcentral.nih.gov/articlerender.fcgi?artid=4681843&tool=pmcentrez&rendertype=abstract
